# Diarylheptanoid Derivatives (Musellins A–F) and Dimeric Phenylphenalenones from Seed Coats of *Musella lasiocarpa*, the Chinese Dwarf Banana

**DOI:** 10.1021/acs.jnatprod.3c00273

**Published:** 2023-05-31

**Authors:** Hui Lyu, Yu Chen, Jonathan Gershenzon, Christian Paetz

**Affiliations:** † NMR/Biosynthesis Group, Max-Planck-Institute for Chemical Ecology, 07745 Jena, Germany; ‡ Jiangsu Key Laboratory for the Research and Utilization of Plant Resources, Institute of Botany, Jiangsu Province and Chinese Academy of Sciences (Nanjing Botanical Garden Mem. Sun Yat-Sen), 210014 Nanjing, China; § Department of Biochemistry, Max-Planck-Institute for Chemical Ecology, 07745 Jena, Germany

## Abstract

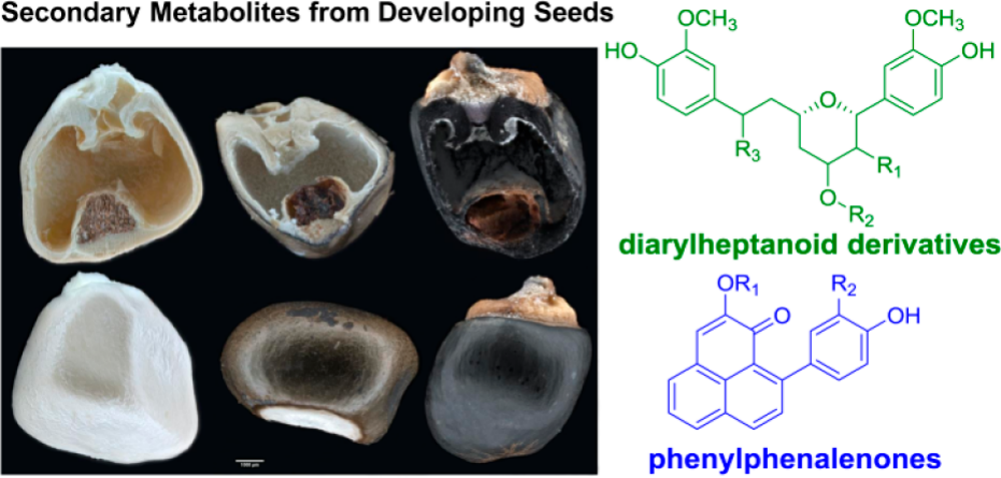

Phenylphenalenones (PPs) are phytoalexins protecting banana plants (Musaceae) against various pathogens. However, how plants synthesize PPs is still poorly understood. In this work, we investigated the major secondary metabolites of developing seed coats of *Musella lasiocarpa* to determine if this species might be a good model system to study the biosynthesis of PPs. We found that PPs are major components of *M. lasiocarpa* seed coats at middle and late developmental stages. Two previously undescribed PP dimers (**M-4** and **M-6**) and a group of unreported diarylheptanoid (DH) derivatives named musellins A–F (**B-7**, **B-9**, **B-10**, **B-12**, **B-14**, and **B-15**) were isolated along with 14 known compounds. Musellin D (**B-12**) and musellin F (**B-15**) contain the first reported furo[3,2-*c*]pyran ring and represent a previously undescribed carbon skeleton. The chemical structures of all new compounds were characterized by spectroscopic data, including NMR, HRESIMS, and ECD analysis. Plausible biosynthetic pathways for the formation of PPs and DHs are proposed.

Phenylphenalenones (PPs) are complex phenolic natural products with a tricyclic phenalene-1*H*-one nucleus and an attached phenyl group. They are reported from four monocotyledonous families: Haemodoraceae, Musaceae, Pontederiaceae, and Strelitziaceae.^1,2^ As phytoalexins, PPs play an important role in the natural defense system of banana species (Musaceae) against multiple pathogens.^2,3^ The suggested biosynthetic precursors of PPs are the diarylheptanoids (DHs), known from many plant families including Zingiberaceae, Betulaceae, Myricaceae, Aceraceae, and Juglandaceae.^4^ DHs have been shown to exhibit diverse pharmacological activities, including anti-inflammatory, pro-apoptotic, anti-influenza, antiemetic, anticancer, and estrogenic activities.^5^

Several lines of evidence indicate that DHs serve as precursors in the biosynthesis of PPs. In a chemical study, a linear DH (compound **2**) was cyclized by chemical oxidation to 6-hydroxy **1** (Scheme 1a).^6^ In addition, isotope labeling experiments with root cultures of *Anigozanthos preissii* (Haemodoraceae) suggested that a linear DH (compound **2**) is the direct precursor of a PP (compound **1**) (Scheme 1b).^7^ However, there are no reports of linear DHs in PP-containing Haemodoraceae. Moreover, although the biosynthesis of curcuminoids, well-known linear DHs found in *Curcuma longa* (Zingiberaceae),^8^ has been extensively studied, no evidence for the occurrence of PPs has yet been found in the Zingiberaceae. An ideal plant system to study the biosynthesis of PPs should contain both DHs and PPs in ample amounts.

**Scheme 1:**
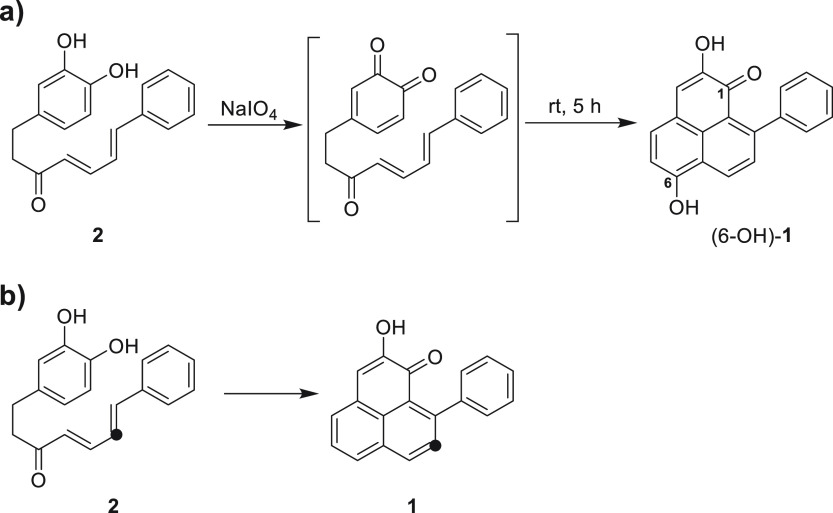
(a) A Linear DH (Compound **2**) Was Cyclized to PP (6-Hydroxy **1**) by Chemical Oxidation;^6^ (b) Biosynthesis of ^13^C-Linear DH (Compound **2**) to ^13^C-PP (Compound **1**) in a Root Culture of *A. preissii*(7)

*Musella lasiocarpa* (Musaceae), the Chinese dwarf banana, is a close relative to bananas (*Musa*) and native to southwestern China, growing at altitudes of 1500–2500 m.^9^ It is used by local people for various purposes, including fodder, erosion control, weaving material, an edible vegetable, and folk medicine.^9^ Interestingly, a previous phytochemical investigation of the aerial parts of *M. lasiocarpa* led to the isolation of PPs, linear DHs, and musellarins (bicyclic DHs), among others.^10^ In a preliminary study, we found PPs to be major components of mature seed coats. Because the seeds did not contain interfering metabolites such as chalcones, flavonoids, or stilbenes, we investigated *M. lasiocarpa* seeds further to see if they could serve as a model system to study PP biosynthesis.

In this study, the secondary metabolites of *M. lasiocarpa* seed coats were analyzed at three developmental stages, including yellow, brown, and mature black seeds. We describe the isolation and identification of two previously undescribed PP dimers (**M-4** and **M-6**) and a group of unreported DH derivatives named musellins A–F (**B-7**, **B-9**, **B-10**, **B-12**, **B-14**, and **B-15**) along with 14 known compounds from these seeds. Additionally, we propose a hypothetical scheme for the biosynthesis of PPs and DHs during seed maturation. The results of this research document the metabolic changes in the phenolic composition of *M. lasiocarpa* seeds and provide a basis for further investigation of PP biosynthesis.

## Results and Discussion

**Figure 1:**
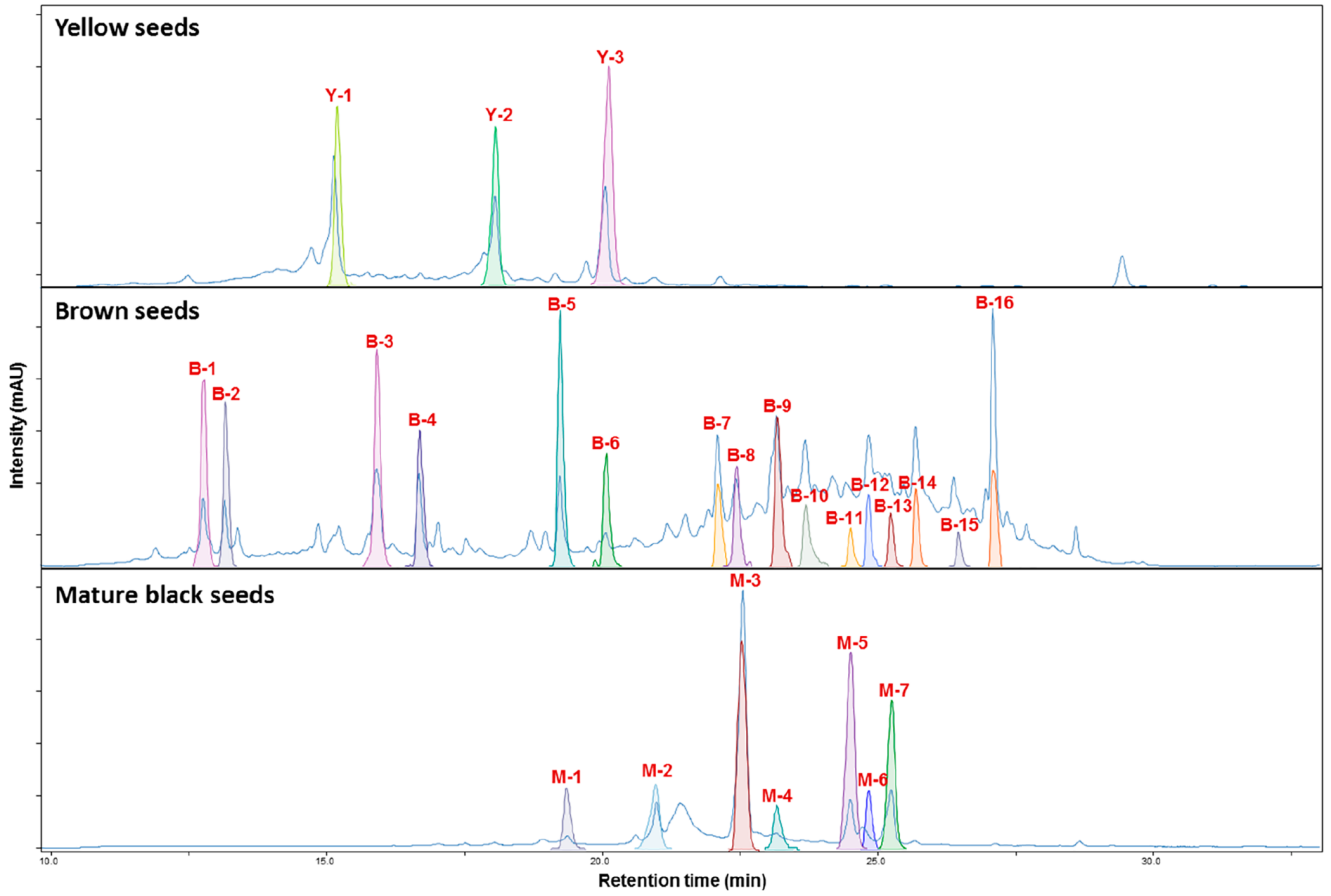
HPLC-UV profile (λ = 190–600 nm) of MeOH extracts of seed coats of *M. lasiocarpa* at different developmental stages (yellow, brown, and mature black).

The metabolic profiles of *M. lasiocarpa* seeds showed distinct changes at different stages of development (Figure 1). Phytochemical analysis of yellow seed coats (the youngest stage) revealed the presence of several common compounds including tryptophan (**Y-1**), (+)-catechin (**Y-2**), and (+)-afzelechin (**Y-3**). On the other hand, the brown seed coats (an intermediate stage) contained the known compound (+)-afzelechin (**Y-3**/**B-6**) (shared with the yellow seed coats) and three known PPs shared with black seed coats, 2-methoxy-9-(3′,4′-dihydroxyphenyl)-1*H*-phenalen-1-one (**B-8**/**M-3**),^10^ 2-methoxy-9-(4′-hydroxyphenyl)-1*H*-phenalen-1-one (**B-11**/**M-5**),^10^ and 2-hydroxy-9-(4′-hydroxyphenyl)-1*H*-phenalen-1-one (hydroxyanigorufone, **B-13**/**M-7**).^10^ Brown seed coats also contained vanillic acid glucosyl ester (**B-1**),^11^ syringic acid glucosyl ester (**B-2**),^12^ vanillic acid (**B-3**), coumaric acid (**B-4**), and cinnamic acid (**B-5**). In addition, five structurally related DHs (musellins A, B, C, and E: **B-7**, **B-9**, **B-10**, and **B-14**, musaitinerin A: **B-16**) with a pyran ring (a 1,5-oxo bridge) in their seven-membered carbon chain, the DH derivative musellin D (**B-12**) with a furo[3,2-*c*]pyran ring, and the complex musellin F (**B-15**) containing both pyran and furo[3,2-*c*]pyran rings were also isolated from brown seed coats. Finally, mature black seed coats (the oldest stage) were found to contain two previously undescribed PP dimers (**M-4** and **M-6**), a linear DH (putative precursor of PPs), identified as (4*E*,6*E*)-1-(3′,4′-dihydroxyphenyl)-7-(4″-hydroxyphenyl)hepta-4,6-dien-3-one (**M-1**),^10^ and four known PPs, identified as 2-(4′-hydroxyphenyl)-1,8-naphthalic anhydride (**M-2**),^13^ 2-methoxy-9-(3′,4′-dihydroxyphenyl)-1*H*-phenalen-1-one (**M-3/B-8**),^10^ 2-methoxy-9-(4′-hydroxyphenyl)-1*H*-phenalen-1-one (**M-5/B-11**),^10^ and 2-hydroxy-9-(4′-hydroxyphenyl)-1*H*-phenalen-1-one (hydroxyanigorufone, **M-7/B-13**).^10^ All previously undescribed structures, musellins A–F (**B-7**, **B-9**, **B-10**, **B-12**, **B-14**, and **B-15**) and PP dimers (**M-4** and **M-6**), were elucidated by analysis of their spectroscopic data. Known compounds were identified either by comparing their spectroscopic data to previously reported data or by co-injection experiments with commercially available references.

**Figure 2:**
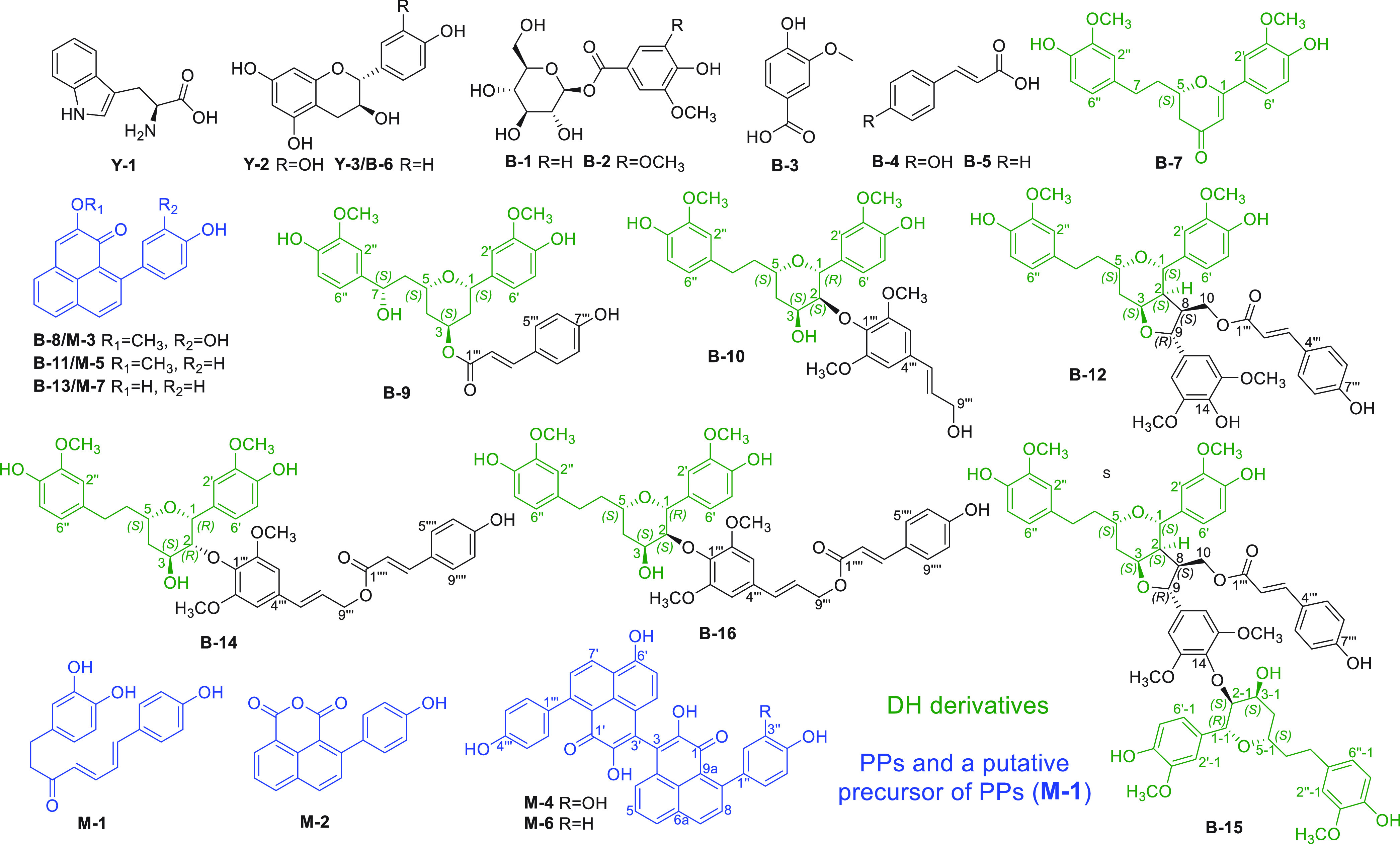
Structures of compounds **Y-1** to **Y-3**, **B-1** to **B-16**, and **M-1** to **M-7** isolated from yellow, brown, and mature black seed coats of *M. lasiocarpa*, respectively. Green structures represent DH derivatives; blue structures represent PPs and a putative precursor; black structures are other structure classes.

**Figure 3:**
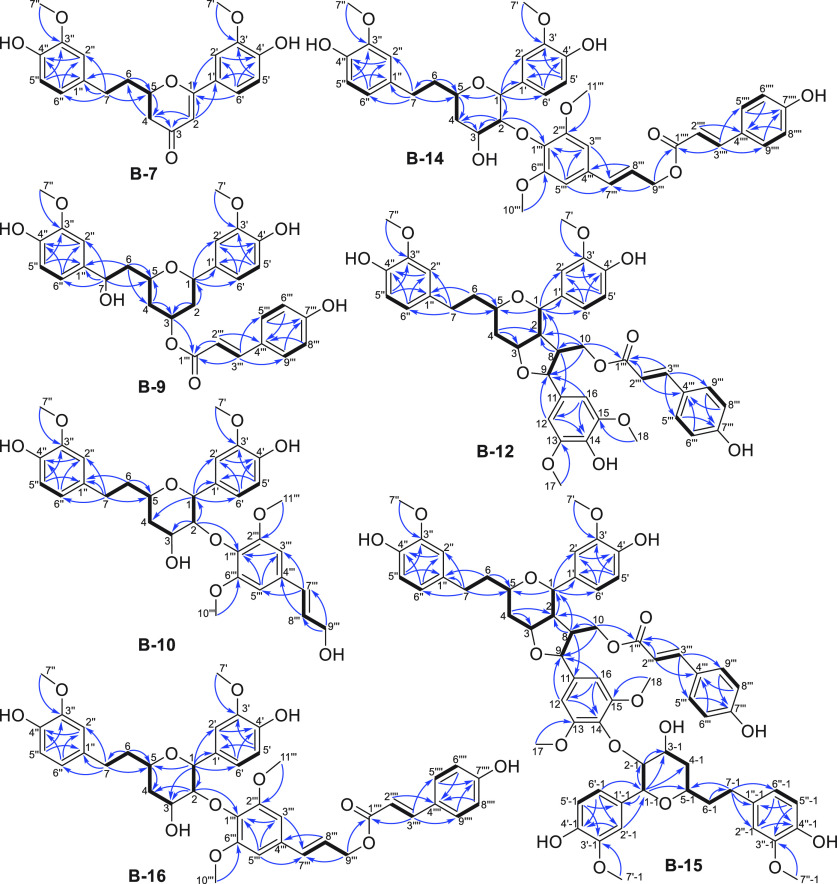
Key HMBC (blue arrows) and COSY (bold lines) correlations of compounds **B-7**, **B-9**, **B-10**, **B-12**, **B-14**, **B-15**, and **B-16**.

Musellin A (**B-7**) has a molecular formula of C_21_H_22_O_6_, with 11 degrees of unsaturation, as determined by (−)-HRESIMS analysis. The ^1^H NMR spectrum combined with HSQC correlations (Table 1) indicated the presence of two methoxy groups, an olefinic proton, an oxygenated methine proton, and three pairs of methylene protons. Six aromatic protons represent two 1,3,4-trisubstituted benzene rings. The resulting spin systems H-5′/H-6′, H-5″/H-6″, and H_2_-4/H-5/H_2_-6/H_2_-7 were suggested by COSY correlations as shown in Figure 3. Analysis of the NMR spectra revealed 21 carbon resonances (Table 2): one carbonyl carbon, five oxygenated nonprotonated sp^2^ carbons, two nonprotonated sp^2^ carbons, seven sp^2^ methines, one sp^3^ oxymethine, two methoxy carbons, and three sp^3^ methylenes. The HMBC spectrum showing the correlations of H_2_-7 with C-1″/C-2″/C-6″/C-5, H_2_-6 with C-1″/C-5, H_2_-4 with C-3/C-6, H-2 with C-3/C-4/C-1/C-1′, H-2′/H-6′ with C-1, H-2′ with C-4′, H-5′ with C-1′/C-3′, H-6′ with C-2′/C-4′, H-2″ with C-4″, H-5″ with C-1″/C-3″, and H-6″ with C-2″/C-4″ proved that **B-7** contained a DH skeleton (Figure 3).^14^ C-5 and C-1 were both oxygenated, while one oxygen and one degree of unsaturation remained, indicating the presence of a dihydropyranyl ring as part of the seven-membered carbon chain. The linkages of OCH_3_-7′ to C-3′ and OCH_3_-7″ to C-3″ were proposed by HMBC correlations (Figure 3). The planar structure of **B-7** was thus established (Figure 2). The calculated electronic circular dichroism (ECD) and UV data were in good agreement with the experimental data (Figure 5), leading to the assignment of the absolute configuration of **B-7** as 5*S*.

**Figure 4:**
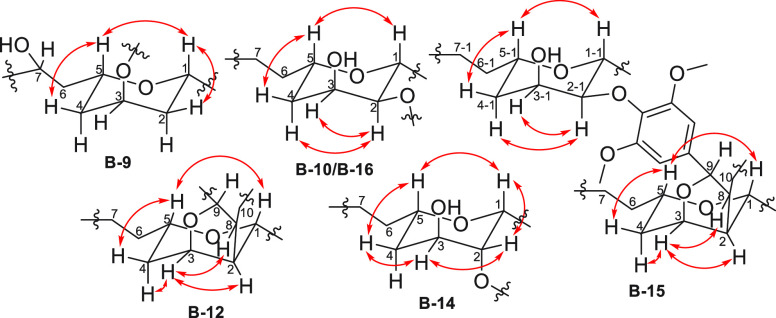
Key ROESY correlations of compounds **B-9**, **B-10**, **B-12**, **B-14**, **B-15**, and **B-16**.

**Figure 5:**
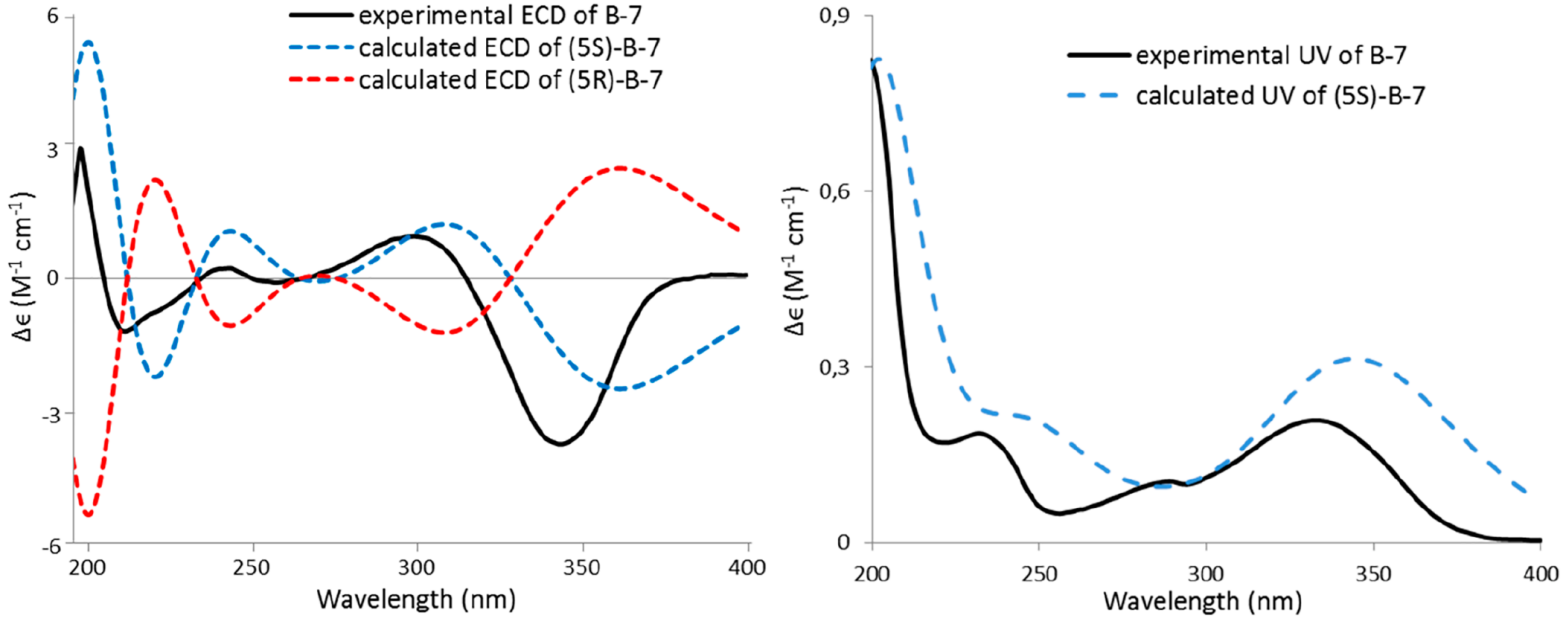
Comparison of experimental and calculated ECD (left) and UV (right) spectra of **B-7**.

**Table 1:** ^1^H (700 MHz) NMR Data for Compounds **B-7**, **B-9**, **B-10**, and **B-16** in CD_3_OH and **B-14** in CD_3_CN

	B-7	B-9	B-10	B-14	B-16
no.	δ_H_, mult. (*J* in Hz)	δ_H_, mult. (*J* in Hz)	δ_H_, mult. (*J* in Hz)	δ_H_, mult. (*J* in Hz)	δ_H_, mult. (*J* in Hz)
1		4.62, dd (11.7,1.9)	4.67, d (9.6)	4.81, d (1.2)	4.67, d (9.5)
2	5.97, d (0.8)	α 1.87, ddd (14.0,11.7,2.3)	4.21, dd (9.6,1.6)	4.21, dd (2.9,1.2)	4.19, dd (9.5,2.0)
		β 1.98, bd (14.0)			
3		5.30, dd (2.3,2.3)	4.02, m	3.84, ddd (2.9, 2.6, 2.6)	3.97, br s
4α	2.60, dd (17.0, 13.6)	1.70, ddd (14.0, 11.9, 2.3)	1.61, m	2.25, ddd (13.8, 11.9, 2.6)	1.56, ddd (13.9, 11.9, 2.0)
4β	2.47, ddd (17.0,3.3,0.8)	1.84, bd (14.0)	1.92, m	1.52, ddd (13.8,2.6,2.3)	1.89, ddd (13.9.2.0,2.0)
5	4.52, m	3.83, m	3.90, m	3.78, m	3.89, m
6a	2.25, m	2.11, ddd (13.7, 9.0, 6.7)	1.76, m	1.90, m	1.73, m
6b	2.05, m	1.76, ddd (13.7, 7.8, 4.4)	1.67, m	1.76, m	1.64, m
7a	2.82, m	4.83, dd (7.8, 6.7)	2.60, m	2.76, ddd (13.9, 9.1, 4.9)	2.57, m
7b	2.88, m		2.60, m	2.69, ddd (13.9, 8.9, 7.6)	2.57, m
2′	7.35, d (1.8)	6.97, d (1.7)	7.04, d (1.8)	7.11, d (1.5)	7.03, d (1.7)
5′	6.87, d (8.6)	6.78, d (8.1)	6.77, d (8.0)	6.79, d (8.2)	6.78, d (8.2)
6′	7.35, dd (8.6, 1.8)	6.81, dd (8.1, 1.7)	6.94, dd (8.0, 1.8)	6.94, dd (8.2, 1.5)	6.94, dd (8.2, 1.7)
7′	3.90, s	3.87, s	3.85, s	3.83, s	3.81, s
2″	6.83, d (2.1)	6.92, d (1.6)	6.73, d (1.8)	6.83, d (1.9)	6.70, d (1.4)
5″	6.71, d (8.1)	6.78, d (8.2)	6.67, d (8.2)	6.71, d (8.0)	6.67, d (8.2)
6″	6.69, dd (8.1,2.1)	6.79, dd (8.2,1.6)	6.59, dd (8.2,1.8)	6.68, dd (8.0,1.9)	6.57, dd (8.2,1.4)
7″	3.78, s	3.78, s	3.79, s	3.77, s	3.76, s
2‴		6.33, d (15.8)			
3‴		7.56, d (15.8)	6.64, s	6.64, s	6.62, s
5‴		7.44, d (8.8)	6.64, s	6.64, s	6.62, s
6‴		6.82, d (8.8)			
7‴			6.49, d (16.0)	6.59, d (15.9)	6.56, d (15.1)
8‴		6.82, d (8.8)	6.27, ddd (16.0,5.7,5.7)	6.31, ddd (15.9,6.3,6.2)	6.29, dt (15.1,6.3)
9‴		7.44, d (8.8)	4.20, dd (5.7,1.5)	4.78, dd (6.3,6.2)	4.77, d (6.3)
10‴/11‴			3.67, s	3.65, s	3.63, s
2⁗				6.37, d (15.8)	6.32, d (16.0)
3⁗				7.64, d (15.8)	7.61, d (16.0)
5⁗/9⁗				7.50, d (8.4)	7.41, d (8.2)
6⁗/8⁗				6.84, d (8.4)	6.78, d (8.2)

**Table 2:** ^13^C (175 MHz) NMR Data for Compounds **B-7**, **B-9**, **B-10**, and **B-16** in CD_3_OH and **B-14** in CD_3_CN^a^

	B-7	B-9	B-10	B-14	B-16
no.	δ_C_, type	δ_C_, type	δ_C_, type	δ_C_, type	δ_C_, type
1	173.0, C	75.6, CH	77.6, CH	76.4, CH	78.1, CH
2	100.1, CH	38.2, CH_2_	82.4, CH	80.1, CH	82.8, CH
3	196.1, C	68.9, CH	66.3, CH	65.7, CH	66.7, CH
4	41.5, CH_2_	36.4, CH_2_	37.9, CH_2_	34.6, CH_2_	38.3, CH_2_
5	79.8, CH	72.1, CH	71.7, CH	72.2, CH	72.2, CH
6	37.0, CH_2_	45.9, CH_2_	38.3, CH_2_	38.9, CH_2_	38.7, CH_2_
7	31.8, CH_2_	72.5, CH	32.0, CH_2_	32.0, CH_2_	32.4, CH_2_
1′	124.6, C	134.8, C	132.5, C	133.2, C	133.0, C
2′	110.5, CH	110.6, CH	112.7, CH	111.3, CH	113.3, CH
3′	148.9, C	148.6, C	147.8, C	147.2, C	148.2, C
4′	151.8, C	146.8, C	147.1, C	145.8, C	147.2, C
5′	116.2, CH	115.7, CH	115.0, CH	114.5, CH	115.5, CH
6′	121.7, CH	119.5, CH	122.1, CH	120.2, CH	122.6, CH
7′	56.1, CH_3_	56.0, CH_3_	56.0, CH_3_	56.6, CH_3_	56.5, CH_3_
1″	133.5, C	136.6, C	134.5, C	135.0, C	135.1, C
2″	112.8, CH	110.6, CH	112.9, CH	113.0, CH	113.2, CH
3″	148.6, C	148.6, C	148.4, C	147.8, C	148.8, C
4″	145.6, C	146.8, C	145.2, C	144.9, C	145.5, C
5″	116.0, CH	115.7, CH	115.7, CH	115.3, CH	116.2, CH
6″	121.5, CH	119.5, CH	121.4, CH	121.6, CH	121.9, CH
7″	55.9, CH_3_	55.9, CH_3_	55.8, CH_3_	56.3, CH_3_	56.4, CH_3_
1‴		167.9, C	130.8, C	136.3, C	135.3, C
2‴		115.0, CH	154.0, C	154.3, C	154.6, C
3‴		146.2, CH	104.5, CH	104.6, CH	105.1, CH
4‴		126.6, C	134.5, C	132.7, C	134.2, C
5‴		131.0, CH	104.5, CH	104.6, CH	105.1, CH
6‴		116.6, CH	154.0, C	154.3, C	154.6, C
7‴		161.0, C	130.8, CH	134.1, CH	134.7, CH
8‴		116.6, CH	129.5, CH	124.1, CH	124.6, CH
9‴		131.0, CH	63.3, CH_2_	65.3, CH_2_	66.0, CH_2_
10‴/11‴			56.2, CH_3_	56.2, CH_3_	56.7, CH_3_
1⁗				167.5, C	169.0, C
2⁗				115.6, CH	115.0, CH
3⁗				145.5, CH	146.9, CH
4⁗				126.9, C	127.0, C
5⁗/9⁗				131.0, CH	131.3, CH
6⁗/8⁗				116.6, CH	116.9, CH
7⁗				159.9, C	161.3, C

a The ^13^C chemical shifts were determined by a combination of HSQC and HMBC analysis.

**Figure 6:**
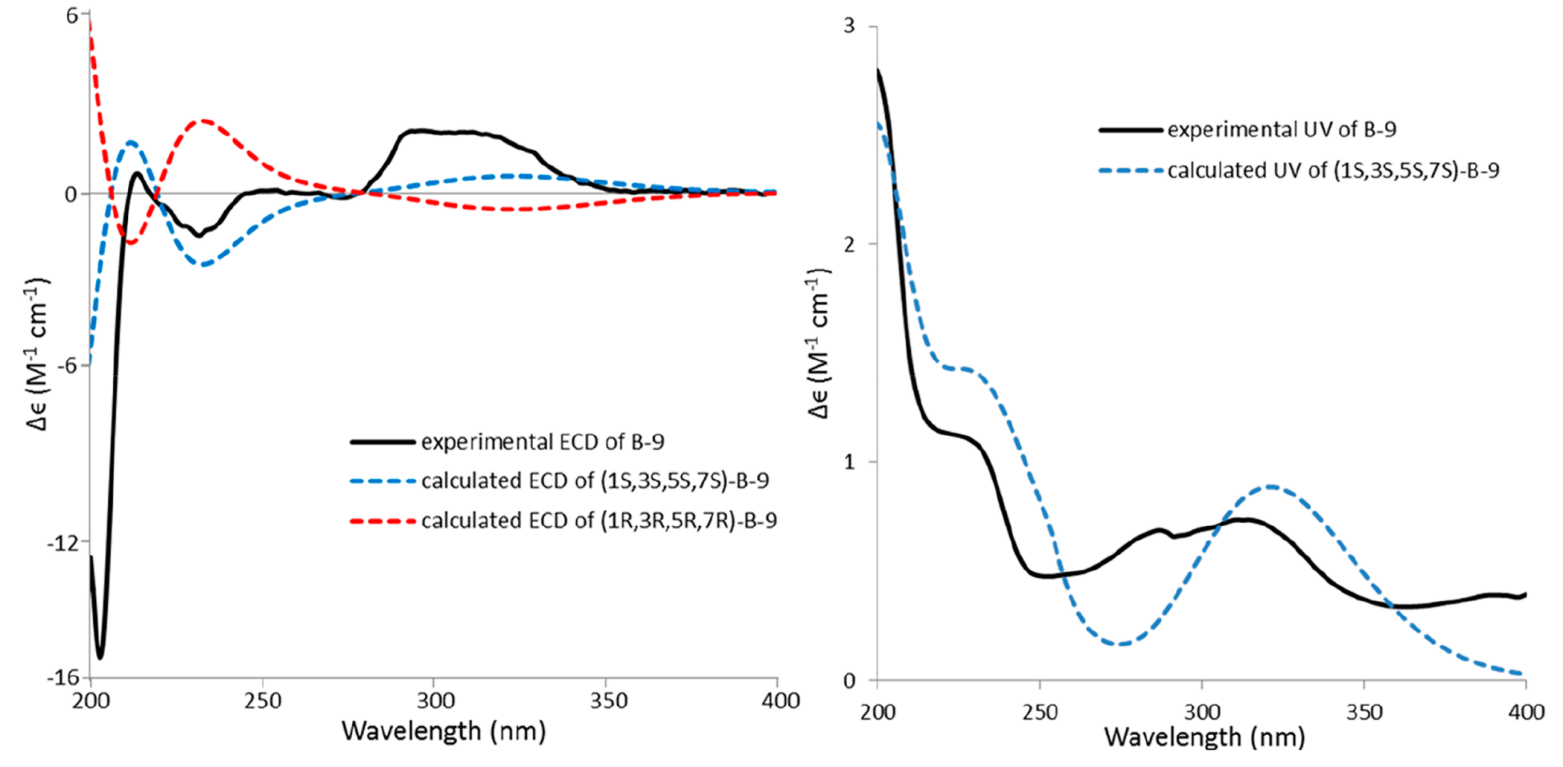
Comparison of experimental and calculated ECD (left) and UV (right) spectra of **B-9**.

Musellin B (**B-9**) has a chemical formula of C_30_H_32_O_9_ based on (−)-HRESIMS data, suggesting 15 degrees of unsaturation. A comparison of the NMR data (Tables 1 and 2) of **B-9** with those of **B-7** revealed that they shared the same DH carbon skeleton, but with a tetrahydropyran moiety. The differences between them were three oxygenated methines at CH-1 (δ_H_ 4.62, δ_C_ 75.6), CH-3 (δ_H_ 5.30, δ_C_ 68.9), and CH-7 (δ_H_ 4.83, δ_C_ 72.5) and one methylene at CH_2_-2 (δ_H_ 1.87/1.98, δ_C_ 38.2) in **B-9**. In addition, a series of resonances (C-1‴–9‴) were assigned to a *p*-coumaroyl substituent by the HMBC correlations of H-2‴ with C-1‴/C-4‴, H-3‴ with C-1‴/C-5‴/C-9‴, H-6‴/H-8‴ with C-4‴, and H-5‴/H-9‴ with C-7‴ (Figure 3). The substituent was located at C-3, as shown by the HMBC correlation of H-3 with C-1‴ (Figure 3). Thus, the planar structure of **B-9** was proposed (Figure 2). The relative configuration of **B-9** was assigned based on coupling constants and ROESY data. The ROESY spectrum showed that H-1, H-2β, H-4β, and H-5 were on the same side of the molecule, tentatively assigned as β (Figure 4). In addition, the small values of the coupling constants *J*_3,2α_ (2.3 Hz) and *J*_3,4α_ (2.3 Hz) were used to assign the α-orientation of H-3. The calculated ECD and UV data were in good agreement with the experimental data (Figure 6). We also calculated the ECD spectrum of the compound isomeric at position 7 (Figure S15), which led to the definition of the absolute configuration of **B-9** as 1*S*, 3*S*, 5*S*, 7*S*.

Musellin C (**B-10**) was assigned the molecular formula C_32_H_38_O_10_ on the basis of (−)-HRESIMS data. Analysis of its 1D and 2D NMR data (Tables 1 and 2) revealed the presence of the same DH backbone as found in **B-9**. In addition, signals typical of a sinapyl alcohol (C-1‴–11‴) were present. The HMBC correlation from H-2 to C-1‴ suggested that the sinapyl alcohol was located at C-2, connected to the phenolic oxygen via an ether bond (Figure 3). Analysis of the HSQC and HMBC spectra revealed additional differences between **B-9** and **B-10**. An oxygenated methine at C-7, a *p*-coumaroyl group located at C-3, and a methylene at C-2 in **B-9** were replaced by a methylene at CH_2_-7 (δ_H_ 2.60, δ_C_ 32.0), an oxygenated methine at C-3 (δ_H_ 4.02, δ_C_ 66.3), and a sinapyl alcohol located at C-2. In addition, the relative configuration was assigned from the ROESY data. The ROESY correlations between H-1, H-5, and H-4β indicated a cofacial arrangement, and these substituents were tentatively assigned as β-oriented, while H-2, H-3, and H-4α were assigned as α-oriented (Figure 4). The ECD spectrum (Figure S23) of **B-10** showed a negative Cotton effect (CE) at 236 nm, allowing assignment to an 1*R* absolute configuration based on comparison to ECD spectra for related DHs.^15^ Consequently, the absolute configuration of **B-10** was assigned as 1*R*, 2*S*, 3*S*, 5*S* based on the ROESY correlations.

**Figure 7:**
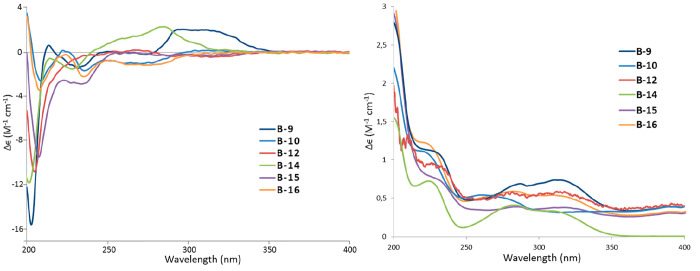
Experimental ECD (left) and UV (right) spectra of compounds **B-9**, **B-10**, **B-12**, **B-14**, **B-15**, and **B-16**.

The molecular formula of **B-16** was deduced from (−)-HRESIMS data as C_41_H_44_O_12_, indicating 20 degrees of unsaturation. The 1D and 2D NMR data (Tables 1 and 2) of this compound were very similar to those of **B-10**, except for the presence of an additional coumaroyl group (C-1⁗–9⁗). This was located at C-9‴, as shown by the HMBC correlation of H-9‴/C-1⁗ (Figure 3). The ROESY correlations showed that the relative configuration of the **B-16** tetrahydropyran ring was identical to that of **B-10** (Figure 4). Liu et al. named it musaitinerin A and reported only its planar structure and relative configuration.^16^ Because **B-16** had identical ECD data to **B-10** (Figure 7), we were able to assign the absolute configuration of **B-16** as 1*R*, 2*S*, 3*S*, 5*S*.

Musellin E (**B-14**) had a molecular formula of C_41_H_44_O_12_ as determined by (−)-HRESIMS data, which is the same as **B-16**. The NMR spectra (Tables 1 and 2) of this compound were similar to those of **B-16**, except for the proton coupling constant *J*_1,2_ (1.2 Hz). The ROESY correlations of H-1 to H-5/H-2 and H-4β to H-5 indicated that H-1, H-2, H-4β, and H-5 were cofacial and thus tentatively assigned as β-oriented (Figure 4). In addition, the small values of the coupling constants *J*_3,2_ (2.9 Hz), *J*_3,4β_ (2.6 Hz), and *J*_3,4α_ (2.6 Hz) were used to assign the α-orientation of H-3. We concluded that **B-14** and **B-16** are a pair of stereoisomers with different configuration at C-2. The ECD spectrum (Figure 7 and Figure S42) of **B-14** showed a negative CE at 228 nm, which allowed the assignment of the absolute configuration as 1*R*.^15^ Consequently, the absolute configuration of **B-14** was assigned as 1*R*, 2*R*, 3*S*, 5*S*.

Musellin D (**B-12**) was found to have the same molecular formula as **B-14** and **B-16**, C_41_H_44_O_12_, based on the analysis of its (−)-HRESIMS data. The NMR data (Table 3) of **B-12** revealed the presence of two substituents on the tetrahydropyranyl DH skeleton, a modified sinapyl moiety and a *p*-coumarate. The tetrahydropyranyl DH skeleton (C-1–7, C-1′–7′, and C-1″–7″) was constructed from the COSY spectrum and contained three spin systems: H_2_-7/H_2_-6/H-5/H_2_-4/H-3/H-2/H-1, H-5′/H-6′, and H-5″/H-6″ (Figure 3). The HMBC correlations of H_2_-7 with C-1″/C-2″/C-6″/C-5, H_2_-6 with C-1″/C-5, H_2_-4 with C-3/C-2, H-1 with C-5/C-1′/C-2′/C-6′, H-2′ with C-4′, H-5′ with C-1′/C-3′, H-6′ with C-2′/C-4′, OCH_3_-7′ with C-3′, H-2″ with C-4″, H-5″ with C-1″/C-3″, H-6″ with C-2″/C-4″, and OCH_3_-7″ with C-3″ characterized the DH scaffold (Figure 3). The modified sinapyl structure (C-8–18) attached to the DH was characterized by COSY correlations of the spin system (H-9/H-8/H_2_-10) (Figure 3) and the HMBC correlations of H-8 with C-1/C-2/C-11, H_2_-10 with C-2/C-8/C-9/C-1‴, H-12/H-16 with C-9/C-14, H-16 with C-12, and OCH_3_-17/18 with C-13/15 (Figure 3). The *p*-coumaroyl substituent (C-1‴–7‴) was attached to the sinapyl structure as shown by the HMBC correlation of H_2_-10 with C-1‴ (Figure 3). Furthermore, C-3 of the tetrahydropyranyl DH and C-9 of the modified sinapyl moiety were both oxygenated methines, while only oxygen and one degree of unsaturation remained, indicating the presence of a furan ring. Consequently, **B-12** was elucidated to contain an unprecedented furo[3,2-*c*]pyran ring system (Figure 2). The relative configuration of **B-12** was elucidated from the coupling constants and the ROESY spectrum. First, the coupling constant of *J*_H-9/H-8_ (10.2 Hz) indicated that H-9 and H-8 were in a *trans* arrangement. The ROESY correlations of H-5 with H-4β and H-1 indicated their cofacial β-orientation. The ROESY correlations of H-3 with H-4α, H-2, and H-8 assigned these groups as α-oriented (Figure 4). The experimental ECD data showed a negative CE around 205 nm, which was present for all DH structures described in this work (Figure 7). Therefore, we calculated the ECD of the structure assuming an *S* configuration at C-1 (note that the stereodescriptor changed in this structure due to the different substitution pattern compared to compounds **B-10**, **B-14**, and **B-16**). The remaining stereogenic centers were defined as 2*S*, 3*S*, 3*S*, 5*S*, 8*S*, 9*R* based on the ROESY data. Although the shorter wavelength part of the calculated ECD and UV data was in good agreement with the experimental spectra, an overpronounced contribution around 290 nm was observed, probably due to the *p*-coumaroyl substituent (Figure S53). To address this, we calculated the ECD spectrum of a modified structure, **B-12a**, in which the *p*-coumaroyl substituent was replaced by an acetyl substituent (Figure S53). The resulting calculated UV and ECD data of **B-12a** were in good agreement with the experimental data for **B-12** (Figure S53), supporting our assignment of the absolute configuration as 1*S*, 2*S*, 3*S*, 5*S*, 8*S*, 9*R*. It is possible that the conformational space used in our calculations was too limited, preventing the correct calculation of ECD data at higher wavelengths.

**Table 3:** ^1^H (700 MHz) and ^13^C (175 MHz) NMR Data for Compounds **B-12** and **B-15** (CD_3_OH)^a^

	B-12		B-15	
no.	δ_**C**_, type	δ_H_, mult. (*J* in Hz)	δ_C_, type	δ_H_, mult. (*J* in Hz)
1	78.8, CH	4.51, d (10.9)	78.8, CH	4.48, d (11.3)
2	45.1, CH	2.89, ddd (10.9,5.5,4.1)	45.3, CH	2.87, ddd (11.3,5.9,4.1)
3	79.1, CH	4.77, ddd (4.1,2.8,2.4)	78.8, CH	4.72, ddd (5.9,2.8,0.8)
4α	35.1, CH_2_	1.73, ddd (14.2,11.7,2.8)	35.0, CH_2_	1.71, m
4β		2.03, ddd (14.2,2.4,1.9)		2.00, ddd (14.5,2.8,2.0)
5	72.6, CH	3.70, m	72.7, CH	3.67, m
6a	38.5, CH_2_	1.76, m	38.5, CH_2_	1.72, m
6b		1.70, m		1.72, m
7a	31.9, CH_2_	2.62, m	32.0, CH_2_	2.59, m
7b		2.57, m		2.59, m
8	51.2, CH	2.85, m	51.3, CH	2.79, m
9	84.3, CH	4.92, d (10.2)	83.6, CH	4.92, d (9.9)
10a	63.1, CH_2_	3.82, dd (11.6,7.2)	62.9, CH_2_	3.86, dd (11.2,7.2)
10b		3.79, dd (11.6,7.8)		3.75, dd (11.2,7.5)
11	134.4, C		140.1, C	
12/16	104.8, CH	6.69, s	104.8, CH	6.63, s
13/15	148.5, C		154.1, C	
14	136.6, C		134.7, C	
17/18	56.3, CH_3_	3.82, s	56.3, CH_3_	3.65, s
1′	133.7, C		133.6, C	
2′	112.9, CH	7.08, d (1.9)	112.9, CH	7.07, d (1.8)
3′	148.5, C		148.4, C	
4′	147.5, C		147.6, C	
5′	115.7, CH	6.77, d (8.1)	115.9, CH	6.77, d (8.1)
6′	122.0, CH	6.98, dd (8.1,1.9)	122.2, CH	6.97, dd (8.1,1.8)
7′	56.0, CH_3_	3.86, s	56.1, CH_3_	3.86, s
1″	134.4, C		134.6, C	
2″	112.9, CH	6.74, d (1.8)	112.6, CH	6.73, d (1.8)
3″	148.5, C		148.4, C	
4″	145.0, C		145.3, C	
5″	115.7, CH	6.68, d (8.0)	115.7, CH	6.67, d (8.1)
6″	121.5, CH	6.60, dd (8.0,1.8)	121.5, CH	6.59, dd (8.1,1.8)
7″	56.0, CH_3_	3.80, s	55.9, CH_3_	3.79, s
1‴	167.7, C		167.7, C	
2‴	114.3, CH	5.81, d (16.0)	114.2, CH	5.81, d (15.9)
3‴	145.8, CH	7.01, d (16.0)	145.8, CH	7.14, d (15.9)
4‴	126.7, C		126.5, C	
5‴/9‴	130.7, CH	7.30, d (8.7)	130.8, CH	7.32, d (8.8)
6‴/8‴	116.3, CH	6.77, d (8.7)	116.6, CH	6.78, d (8.8)
7‴	160.8, C		161.1, C	
1-1			77.7, CH	4.62, d (9.6)
2-1			82.4, CH	4.10, dd (9.6,2.4)
3-1			66.3, CH	3.88, (ovlp.)
4-1α			37.9, CH_2_	1.38, dd (13.8,11.6)
4-1β				1.74, m
5-1			71.7, CH	3.84, m
6-1a			38.5, CH_2_	1.72, m
6-1b				1.72, m
7-1a			31.9, CH_2_	2.59, m
7-1b				2.59, m
1′-1			132.9, C	
2′-1			112.6, CH	7.01, d (1.8)
3′-1			148.0, C	
4′-1			146.9, C	
5′-1			115.1, CH	6.75, d (8.1)
6′-1			122.3, CH	6.89, dd (8.1,1.8)
7′-1			56.1, CH_3_	3.82, s
1″-1			134.5, C	
2″-1			112.7, CH	6.72, d (1.8)
3″-1			148.4, C	
4″-1			145.3, C	
5″-1			115.7, CH	6.67, d (8.1)
6″-1			121.5, CH	6.59, dd (8.1,1.8)
7″-1			55.9, CH_3_	3.79, s

a The ^13^C chemical shifts were determined by a combination of HSQC and HMBC analysis.

Musellin F (**B-15**) has a molecular formula determined by (−)-HRESIMS as C_62_H_68_O_18_, which requires 29 degrees of unsaturation. The NMR data of **B-15**, as shown in Table 3, suggest that it is closely related to **B-12** except for the presence of another tetrahydropyranyl DH unit (C-1–1–7–1, C-1′-1–7′-1, and C-1″-1–7″-1). This conclusion is supported by the COSY correlations of H-2-1/H-1-1, H_2_-7-1/H_2_-6-1/H-5-1/H_2_-4-1/H-3-1, H-5′-1/H-6′-1, and H-5″-1/H-6″-1 (Figure 3). The HMBC signals of H_2_-7-1 with C-1″-1/C-2″-1/C-6″-1/C-5-1, H-1-1 with C-5-1/C-2-1/C-3-1/C-4-1/C-1′-1, H-2-1 with C-3-1, H-2′-1/H-6′-1 with C-1-1/C-4′-1, H-5′-1 with C-1′-1/C-3′-1, H-6′-1 with C-2′-1, OCH_3_-7′-1 with C-3′-1, H-2″-1 with C-4″-1, H-5″-1 with C-1″-1/C-3″-1, H-6″-1 with C-2″-1/C-4″-1, and OCH_3_-7″-1 with C-3″-1 provided additional evidence for the presence of this moiety (Figure 3). As no HMBC correlation was observed to directly show the connection between this additional tetrahydropyran DH unit and the dimethoxyphenyl substituent, its linkage was inferred from the comparison of the chemical shift data of **B-12** and **B-15**. The observed deshielded shifts for C-11 (from δ_C_ 134.4 in **B-12** to δ_C_ 140.1 in **B-15**) and C-13/C-15 (from δ_C_ 148.5 in **B-12** to δ_C_ 154.1 in **B-15**) and the shielded shift for C-14 (from δ_C_ 136.6 in **B-12** to δ_C_ 134.7 in **B-15**) narrowed the possible connection site to the hydroxy group at C-14. Accordingly, the attachment of the additional tetrahydropyranyl DH to C-14 was established as an ether bond. The ROESY correlations and the coupling constant (*J*_H-9/H-8_ 9.9 Hz) showed that the relative configuration of this part of **B-16** was identical to that of **B-12** (Figure 4). The relative configuration of the additional tetrahydropyranyl DH (C-1-1 to 7-1, C-1′-1 to C6′-1, and C-1″-1 to C6″-1) was deduced from the analysis of its ROESY data: H-1-1, H-5-1, and H-4β-1 were β-oriented, and H-2-1, H-3-1, and H-4α-1 were α-oriented (Figure 4). The experimental ECD spectrum of **B-15** showed two negative CEs at 207 and 234 nm (Figure 7). Compared to the ECD spectrum of **B-12** (Figure 7, the negative CE at 234 nm was contributed by the *R* absolute configuration at C-1-1 in the additional tetrahydropyranyl DH moiety of **B-15**.^15^ Consequently, the absolute configuration was determined to be 1*S*, 2*S*, 3*S*, 5*S*, 8*S*, 9*R*, 1-1*R*, 2-1*S*, 3-1*S*, 5-1*S*.

**Figure 8:**
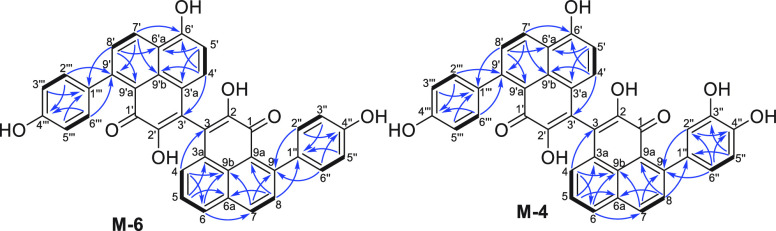
Key HMBC (blue arrows) and COSY (bold lines) correlations of compounds **M-4** and **M-6**.

**M-6** has the molecular formula of C_38_H_22_O_7_ (28 degrees of unsaturation) based on its (+)-HRESIMS peak. The ^1^H NMR spectroscopic data and HSQC correlations revealed characteristic resonances for 17 aromatic protons (Table 4). Eight independent spin systems as shown in Figure 8 of H-4/H-5/H-6, H-7/H-8, H-4′/H-5′, H-7′/H-8′, H-2″(H-6″)/H-3″(H-5″), and H-2‴(H-6‴)/H-3‴(H-5‴), were suggested by the COSY correlations. Analysis of the DEPTQ NMR data and the HSQC spectrum revealed 38 carbon resonances (Table 4), including two carbonyl carbons, five oxygenated nonprotonated sp^2^ carbons, 14 nonprotonated sp^2^ carbons, and 17 sp^2^ methines. The HMBC spectrum showing the correlations of H-4 with C-9b/C-3/C-6, H-5 with C-3a/C-6a, H-6 with C-9b/C-7, H-7 with C-9b/C-9, H-8 with C-6a/C-9a/C-1″, H-2″/H-6″ with C-9/C-4″, H-3″/H-5″with C-1″, H-4′ with C-9′b/C-3′/C-6′, H-5′ with C-3′a/C-6′a, H-7′ with C-6′/C-9′b/C-9′, H-8′ with C-6′a/C-9′a/C-1‴, H-2‴/H-6‴ with C-9′/C-4‴, and H-3‴/H-5‴ with C-1‴ revealed that **M-6** is a dimeric PP (Figure 8).^17^ To identify the positions of C-1, 2, 1′, and 2′, a long-range HMBC experiment was used to detect very (^4–6^*J*_CH_ detection with ^n^*J*_CH_=1 Hz) (Figure S83). Correlations of H-8 with C-1 and H-8′ with C-1′ were observed, providing the positions of C-1 and 1′. No correlation of any proton with C-2 and C-2′ was observed, so their chemical shifts (δ_C_ 148.5 and 146.7) are interchangeable. Thus, the structure of **M-6** was determined as shown in Figure 2.

**Table 4:** ^1^H (700 MHz) and ^13^C (175 MHz) NMR Data for Compounds **M-6** in Acetone-*d*_6_ and **M-4** in CD_3_OH

	M-6		M-4	
no.	δ_C_, type	δ_H_, mult. (J in Hz)	δ_C_, type	δ_H_, mult. (J in Hz)
1	179.8, C		180.8, C	
2	148.5, C^a^		149.2, C^b^	
3	118.1, C		119.3, C	
3a	129.2, C		129.5, C	
4	130.9, CH	7.65, d (7.9)	130.4, CH	7.50, d (7.5)
5	127.4, CH	7.55, dd (7.9,7.9)	127.5, CH	7.41, dd (7.5,7.5)
6	130.2, CH	8.07, d (7.9)	130.8, CH	7.89, d (7.5)
6a	132.2, C		132.6, C	
7	136.2, CH	8.42, d (8.7)	136.3, CH	8.22, d (7.8)
8	132.2, CH	7.68, d (8.7)	132.4, CH	7.58, d (7.8)
9	149.6, C		150.3, C	
9a	124.2, C		124.8, C	
9b	125.9, C		126.3, C	
1″	134.1, C		135.5, C	
2″	130.4, CH	7.34, d (8.7)	116.8, CH	6.90, s
3″	115.5, CH	6.94, d (8.7)	146.0, C	
4″	157.7, C		146.1, C	
5″	115.5, CH	6.94, d (8.7)	116.2, CH	6.86, d (7.5)
6″	130.4, CH	7.34, d (8.7)	120.9, CH	6.77, d (7.5)
1′	179.4, C		180.8, C	
2′	146.7, C^a^		147.3, C^b^	
3′	118.4, C		120.3, C	
3′a	120.4, C		120.4, C	
4′	132.2, CH	7.49, d (8.5)	133.0, CH	7.36, d (8.7)
5′	109.9, CH	6.94, d (8.5)	110.1, CH	6.76, d (8.7)
6′	157.2, C		158.5, C	
6′a	124.3, C		124.8, C	
7′	130.0, CH	8.77, d (8.4)	131.0, CH	8.68, d (7.8)
8′	130.9, CH	7.65, d (8.4)	131.2, CH	7.54, d (7.8)
9′	150.0, C		150.7, C	
9′a	124.1, C		124.8, C	
9′b	126.8, C		127.2, C	
1‴	134.4, C		135.2, C	
2‴/6‴	130.4, CH	7.34, d (8.7)	130.7, CH	7.28, d (8.1)
3‴/5‴	115.4, CH	6.94, d (8.7)	115.8, CH	6.87, d (8.1)
4‴	157.8, C		157.9, C	

a,b Signals may be interchangeable.

**M-4** gave the molecular formula C_38_H_22_O_8_ as determined from (+)-HRESIMS data. It contained one more oxygen than compound **M-6**. The chemical shift of C-3″ was deshielded from δ_C_ 115.5 (**M-6**) to δ_C_ 146.0 (**M-4**), indicating the presence of an additional hydroxy group at C-3″ (Table 4). From the 1D and 2D NMR data, the structure of **M-4** was established as shown in Figure 2.

**Scheme 2:**
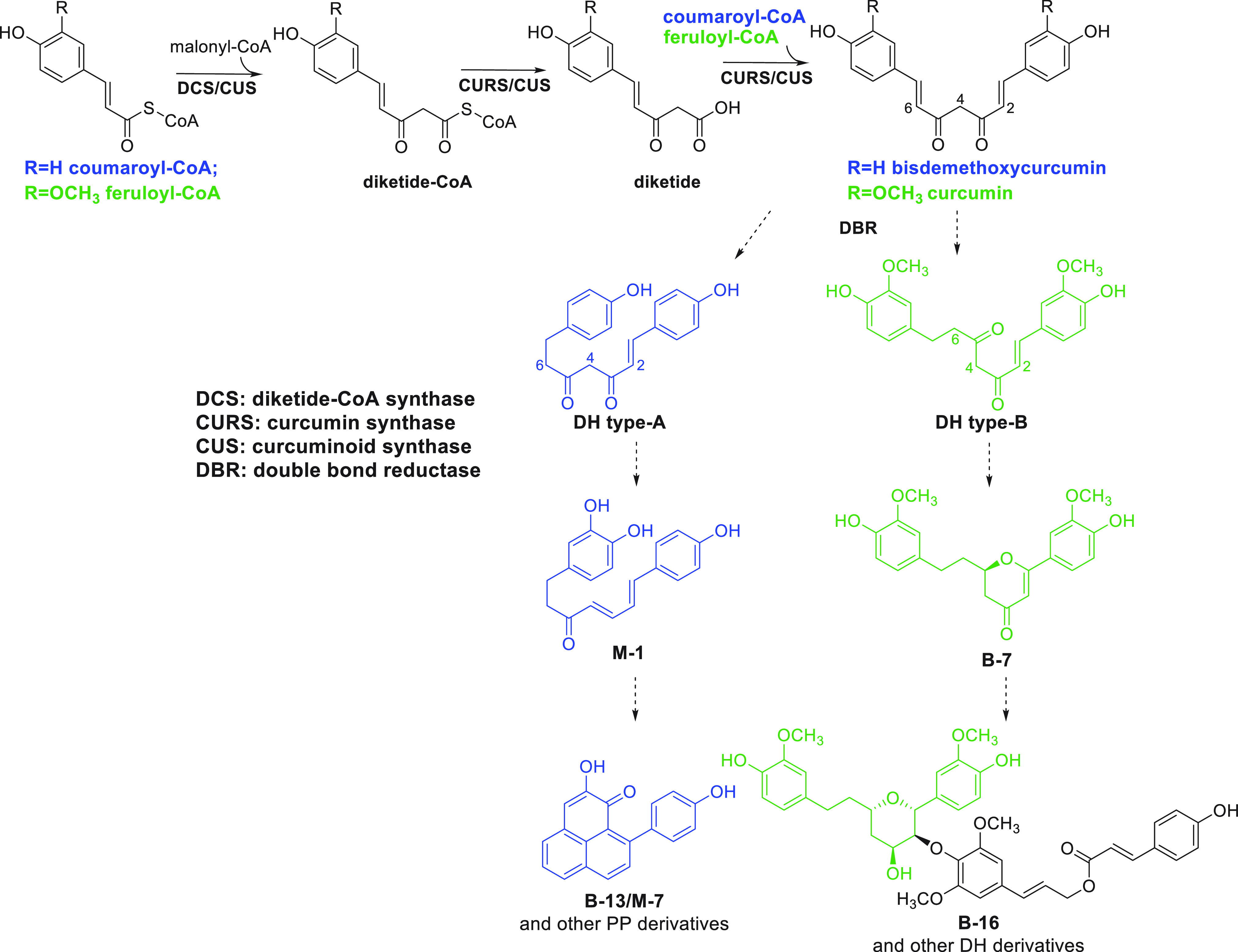
Proposed Biosynthetic Pathway for Phenylphenalenone and Diarylheptanoid Derivatives in *M. lasiocarpa* Seeds

In light of the structures of the DHs and PPs isolated here and in prior work, plus previous biosynthetic studies on a class of DHs, the curcuminoids,^8,18^ a plausible outline for the biosynthesis of DHs and PPs can be proposed (Scheme 2). Based on past work on turmeric (*Curcuma longa*), curcumin biosynthesis involves a two-step process starting with an enzyme called diketide-CoA synthase condensing feruloyl-CoA and malonyl-CoA to form feruloyldiketide-CoA.^8^ Then, curcumin synthase hydrolyzes feruloyldiketide-CoA to feruloyldiketide and catalyzes its coupling with another molecule of feruloyl-CoA to form curcumin.^8^ In contrast, in rice (*Oryza sativa*) curcuminoid formation involves a single enzyme, an unusual type III PKS that produces bisdemethoxycurcumin using two molecules of coumaroyl-CoA and one molecule of malonyl-CoA as substrates.^18^

The formation of the compounds **B-7** to **B-16** and **M-1** to **M-7** isolated in this study seems likely to proceed via the same initial steps proposed for curcumin biosynthesis to form a DH skeleton. Judging from the isotope labeling experiments for PP biosynthesis^19^ and the structures of all isolated DH derivatives, coumaroyl-CoA and feruloyl-CoA appear to be the preferred substrates for the biosynthesis of PP and DH derivatives, respectively. Subsequently, the double bond (Δ^6^) of the bisdemethoxycurcumin or curcumin intermediate must be reduced to form DHs of type A and B, which can then be further modified to produce simple and then more complex DH and PP products. Given that DHs and PPs accumulate in *M. lasiocarpa* seed coats only at middle (brown) and late (black) stages of development, the relevant biosynthetic steps appear to be carried out in seed tissues at middle and late stages. Thus, *M. lasiocarpa* seeds may have considerable potential as a model system for the study of PP biosynthesis.

The major DH derivatives in the brown seeds were curiously undetectable in the MeOH extract of black seeds. These compounds may participate in the polymerization process that creates the exceptionally hard seed coat of mature black seeds. This suggestion is supported by previous studies showing that curcuminoids formed by heterologous expression of the *C. longa* enzymes diketide-CoA synthase and curcumin synthase in *Arabidopsis* and poplar can be integrated into lignin.^20,21^ On the other hand, the PPs present in brown seeds remained as soluble, detectable metabolites in the coats of mature black seeds. These metabolites could serve as chemical defenses against pathogens before and during germination, protecting the developing seedling from harmful microorganisms.

## Experimental Section

### General Experimental Procedures

ECD spectra were measured with a JASCO J-810 spectrometer using a 1 mm path length sample cell. NMR spectra (^1^H NMR with water suppression, DEPTQ, COSY, selective TOCSY, HMBC, HSQC, and ROESY spectra) were measured at 298 K on a Bruker Advance III HD 700 NMR spectrometer, equipped with a 1.7 mm TCI microcryoprobe (Bruker Biospin GmbH). Spectrometer control, data acquisition, and processing were performed using Bruker TopSpin version 3.6.1. Chemical shifts are expressed in δ (ppm) relative to the residual solvent signals of methanol-*d*_3_ (δ_H/C_ 3.31/49.15), acetonitrile-*d*_3_ (δ_H/C_ 1.94/1.39), or acetone-*d*_6_ (δ_H/C_ 2.05/29.92). For HPLC-UV-HRESIMS measurements, an Agilent Infinity 1260 HPLC system consisting of a quaternary pump (G1311B), autosampler (G1367E), column oven (G1316A), and diode array detector (G1315D) was coupled to a Bruker Compact OTOF mass spectrometer. For preparative HPLC, a Shimadzu Prominence HPLC system consisting of a degasser (DGU-20A5), gradient pump (LC-20AT), autosampler (SIL-20AC), column oven (CTO-20A), UV detector (SPD-20A), fraction collector (FRC-10A), and system controller (CBM-20A) was used. All solvents used for extractions and preparative HPLC were HPLC grade, while solvents used for HPLC/MS analysis were of MS grade. All solvents were purchased from VWR. (+)-Afzelechin was purchased from MedChem Express. Tryptophan, (+)-catechin, vanillic acid, coumaric acid, and cinnamic acid were purchased from Fisher Scientific.

### Plant Material

Seeds of *Musella lasiocarpa* were collected at three different developmental stages (yellow, brown, and mature black seeds, respectively) from Chuxiong City, Yunnan Province, China (September 2019). A voucher specimen (No. 20190920) was authenticated by Professor Changqi Yuan (Institute of Botany, Jiangsu Province and Chinese Academy of Sciences) and deposited at the Max Planck Institute for Chemical Ecology. The yellow (outer and inner integument both yellow), brown (brown outer integument, yellow inner integument), and mature black seeds (outer and inner integument both black) were collected, snap-frozen in liquid nitrogen, and then freeze-dried.

### HPLC-UV-HRESIMS Analysis

One freeze-dried seed of each developmental stage was cut in half, and the starch (brown and black stages) inside the seed coats was removed. The samples were transferred to 2 mL Precellys homogenizer tubes containing 1 mL of MeOH and 1.4 mm zirconium dioxide beads, sealed with a screw cap, and homogenized for 3 × 30 s at 5500 rpm in a Bertin Minilys cell disruptor. The homogenates were then centrifuged at 13 000 rpm for 10 min at 25 °C. The supernatants were collected, and the remaining pellet was extracted twice using the same procedure. The combined extracts were diluted with 30 mL (10× volume) of distilled H_2_O and passed through a preconditioned HR-X solid phase extraction (SPE) cartridge (30 mg, 1 mL, Macherey-Nagel). The loaded cartridges were eluted with 2 mL of MeOH, and the eluate was passed through a preconditioned Discovery DPA-6S SPE (50 mg, 1 mL, Supelco) to remove polar macromolecular compounds. The pooled eluates were then dried with N_2_ gas, reconstituted with 150 μL of MeOH, and filtered through a 0.45 μm disk filter. A 10 μL amount of the filtrate was subjected to HPLC-UV-HRESIMS analysis. HPLC conditions were as follows: An Agilent Zorbax C18 column (3.5 μm; 150 × 4.6 mm) was used with the binary gradient conditions (A: H_2_O, B: MeCN, both containing 0.1% formic acid) 0–5 min 5% B, 5–30 min 5–100% B, and 30–35 min 100% B, with a constant flow rate of 500 μL min^–1^. The ESI source parameters were as follows: spray capillary voltage 4.5 kV, dry gas temperature 220 °C, dry gas flow 9.0 L min^–1^, settings used for the analysis of small molecules (full scan range of *m*/*z* 50–1300 with a resolution of 30 000 *m*/Δ*m*).

### Extraction and Isolation

The freeze-dried and starch-free seed coats of *M. lasiocarpa* were separated into yellow, brown, and mature black stages and separately ground to powder using a mill (IKA M20 universal mill). Each material (yellow: 3 g, brown: 10 g, and black: 5 g) was suspended in 50 mL of MeOH and then shaken on a rotary shaker (180 rpm) for 4 h at room temperature. After filtration, the remaining residue was extracted twice more with MeOH (50 mL). The combined MeOH extract was filtered and diluted with 1500 mL (10× volume) of distilled H_2_O. This solution was passed through a preconditioned HRX SPE cartridge (1000 mg, 15 mL, Macherey-Nagel) using an MN PTFE tube adapter. The loaded cartridge was eluted with 10 mL of MeOH, and the eluate was passed through a preconditioned Discovery DPA-6S SPE (500 mg, 6 mL, Supelco). The three MeOH eluates of yellow, brown, and mature black seed coats were evaporated with N_2_ gas to yield residues of 358, 1576, and 707 mg, respectively, which were then reconstituted with MeOH and subjected to separation by HPLC. The HPLC was equipped with a Nucleodur C-18 HTec column (5 μm; 250 × 4.6 mm; Macherey-Nagel) with a constant flow rate of 800 μL min^–1^ at 35 °C and a binary solvent system of H_2_O (solvent A) and MeCN (solvent B), both containing 0.1% (v/v) formic acid. For yellow seed coats, the following gradient was used: 0–5 min 5% MeCN, 5–21 min 5–66% MeCN, and 21–26 min 66–100% MeCN to yield compounds **Y-1** (2.3 mg), **Y-2** (3.1 mg), and **Y-3** (3.5 mg). For brown seed coats, the following gradient was used: 0–5 min 5% MeCN, 5–30 min 5–70% MeCN, 30–50 min 70–100% MeCN, and 50–55 min 100% MeCN to yield compounds **B-1** (0.3 mg), **B-2** (0.4 mg), **B-3** (0.4 mg), **B-4** (0.3 mg), **B-5** (0.5 mg), **B-6** (0.2 mg), **B-7** (0.2 mg), **B-8** (0.3 mg), **B-9** (0.1 mg), **B-10** (0.2 mg), **B-11** (0.2 mg), **B-12** (0.1 mg), **B-13** (0.2 mg), **B-14** (0.1 mg), **B-15** (0.2 mg), and **B-16** (0.2 mg). For mature black seed coats, the following gradient was used: 0–5 min 5% MeCN, 5–15 min 5–50% MeCN, 15–40 min 50–100% MeCN, and 40–45 min 100% MeCN to yield compounds **M-1** (0.3 mg), **M-2** (0.4 mg), **M-3** (1.6 mg), **M-4** (0.5 mg), **M-5** (1.4 mg), **M-6** (0.5 mg), and **M-7** (1.2 mg).

### ECD Computational Methods

Calculations of ECD spectra of **B-7**, **B-9**, and **B-12** were performed using the Gaussian 16 software.^22^ The structures were initially optimized at the semiempirical PM6 level. Conformers were calculated using the grid search algorithm in GMMX, which was embedded in GaussView 6.1.1. The derived structures were then optimized in a DFT calculation using the B3LYP functional at the 6-311+g(d,p) level. We calculated the ECD frequencies of structures within a cutoff level of 2.5 kcal mol^–1^ from the lowest energy structure. Fifty ECD frequencies were calculated using the TD-SCF method with the same functional as used for the geometry optimization described above. ECD curves were calculated in GaussView 6.1.1 after applying Boltzmann weighting to the contributing structures.

#### Musellin A (**B-7**):

white amorphous solid; UV/vis (MeCN–H_2_O) λ_max_ 222, 334 nm; ECD (0.97 mM, MeOH), λ_max_ (Δε) 344 (−3.77), 299 (+0.96), 242 (+0.23), and 211 (−1.20) nm; ^1^H NMR (CD_3_OH, 700 MHz), Table 1; ^13^C NMR (CD_3_OH, 175 MHz), Table 2; (−)-HRESIMS *m*/*z* 369.1343 [M – H]^−^ (calcd for C_21_H_21_O_6_, 369.1344).

#### Musellin B (**B-9**):

white amorphous solid; UV/vis (MeCN–H_2_O) λ_max_ 200, 226, 288, 312 nm; ECD (0.45 mM, MeOH), λ_max_ (Δε) 308 (+1.99), 232 (−1.40), 214 (+0.63), and 203 (−15.25) nm; ^1^H NMR (CD_3_OH, 700 MHz), Table 1; ^13^C NMR (CD_3_OH, 175 MHz), Table 2; (−)-HRESIMS *m*/*z* 535.1978 [M – H]^−^ (calcd for C_30_H_31_O_9_, 535.1974).

#### Musellin C (**B-10**):

white amorphous solid; UV/vis (MeCN–H_2_O) λ_max_ 202, 222, 274 nm; ECD (0.55 mM, MeOH), λ_max_ (Δε) 277 (−0.94), 236 (−1.61), and 209 (−2.54) nm; ^1^H NMR (CD_3_OH, 700 MHz), Table 1; ^13^C NMR (CD_3_OH, 175 MHz), Table 2; (−)-HRESIMS *m*/*z* 581.2399 [M – H]^−^ (calcd C_32_H_37_O_10_, 581.2392) and *m*/*z* 627.2454 [M + HCOO]^−^ (calcd C_33_H_39_O_12_, 627.2447).

#### Musellin D (**B-12**):

white amorphous solid; UV/vis (MeCN–H_2_O) λ_max_ 204, 227, 286, 312 nm; ECD (0.30 mM, MeOH), λ_max_ (Δε) 205 (−10.59) nm; ^1^H NMR (CD_3_OH, 700 MHz), Table 3; ^13^C NMR (CD_3_OH, 175 MHz), Table 3; (−)-HRESIMS *m*/*z* 727.2757 [M – H]^−^ (calcd for C_41_H_43_O_12_, 727.2760).

#### Musellin E (**B-14**):

white amorphous solid; UV/vis (MeCN-H_2_O) λ_max_ 203, 224, 286, 309 nm; ECD (0.22 mM, MeOH), λ_max_ (Δε) 285 (+2.28), 228 (−1.47), and 201 (−11.50) nm; ^1^H NMR (CD_3_CN, 700 MHz), Table 1; ^13^C NMR (CD_3_CN, 175 MHz), Table 2; (−)-HRESIMS *m*/*z* 727.2760 [M – H]^−^ (calcd for C_41_H_43_O_12_, 727.2760).

#### Musellin F (**B-15**)

white amorphous solid; UV/vis (MeCN–H_2_O) λ_max_ 202, 227, 284, 312 nm; ECD (0.25 mM, MeOH), λ_max_ (Δε) 234 (−2.79) and 207 (−9.27) nm; ^1^H NMR (CD_3_OH, 700 MHz), Table 3; ^13^C NMR (CD_3_OH, 175 MHz), Table 3; (−)-HRESIMS *m*/*z* 1099.4347 [M – H]^−^ (calcd for C_62_H_67_O_18_, 1099.4333).

#### Musaitinerin A (**B-16**)

white amorphous solid; UV/vis (MeCN–H_2_O) λ_max_ 203, 224, 286, 309 nm; ECD (0.49 mM, MeOH), λ_max_ (Δε) 276 (−1.12), 235 (−2.14), and 208 (−3.34) nm; ^1^H NMR (CD_3_OH, 700 MHz), Table 1; ^13^C NMR (CD_3_OH, 175 MHz), Table 2; (−)-HRESIMS *m*/*z* 727.2767 [M – H]^−^ (calcd for C_41_H_43_O_12_, 727.2760).

#### Dimeric phenylphenalenone (**M-4**):

orange-red solid; UV/vis (MeCN–H_2_O) λ_max_ 206, 274, 322, 352, 432 nm; ^1^H NMR (CD_3_OH, 700 MHz), Table 4; ^13^C NMR (CD_3_OH, 175 MHz), Table 4; (+)-HRESIMS *m*/*z* 607.1417 [M + H]^+^ (calcd for C_38_H_23_O_8_, 607.1387).

#### Dimeric phenylphenalenone (**M-6**):

orange-red solid; UV/vis (MeCN–H_2_O) λ_max_ 204, 266, 322, 354, 430 nm; ^1^H NMR (acetone-d_6_, 700 MHz), Table 4; ^13^C NMR (acetone-*d*_6_, 175 MHz), Table 4; (+)-HRESIMS *m*/*z* 591.1468 [M + H]^+^ (calcd for C_38_H_23_O_7_, 591.1438).

## Supporting Information Available

HRESIMS, UV, 1D/2D NMR, and ECD spectra for new compounds; HRESIMS, UV, ^1^H/^13^C NMR data for known compounds (PDF)
