# Supplementary material for: Diarylheptanoid Derivatives (Musellins A–F) and Dimeric Phenylphenalenones from Seed Coats of Musella lasiocarpa, the Chinese Dwarf Banana
Source: J Nat Prod. 2023 May 31;86(6):1571–83. doi: 10.1021/acs.jnatprod.3c00273 (PMC10294256; doi:10.1021/acs.jnatprod.3c00273)
Supplement: Supplementary file 1 — np3c00273_si_001.pdf [file np3c00273_si_001.pdf]

Supporting Information

Diarylheptanoid Derivatives (Musellins A–F) and Dimeric Phenylphenalenones from Seed Coats of *Musella lasiocarpa*, the Chinese Dwarf Banana

Hui Lyu[1], Yu Chen[2], Jonathan Gershenzon[3], Christian Paetz[1]*

[1] NMR/Biosynthesis Group, Max-Planck-Institute for Chemical Ecology, 07745 Jena, Germany

[2] Jiangsu Key Laboratory for the Research and Utilization of Plant Resources, Institute of Botany, Jiangsu Province and Chinese Academy of Sciences (Nanjing Botanical Garden Mem. Sun Yat-Sen), 210014 Nanjing, China

[3] Department of Biochemistry, Max-Planck-Institute for Chemical Ecology, 07745 Jena, Germany

Corresponding Author:

* Phone: +49 (0)3641 57-1600; Email: cpaetz@ice.mpg.de

Table of Contents

Musellin A (B-7): **page 10–18**

Figure S1. Chemical shifts of compound **B-7**.

Figure S2. HR-ESI-MS spectrum of compound **B-7**.

Figure S3. UV/Vis spectrum (MeCN-H_2O) of compound **B-7**.

Figure S4. Experimental ECD (upper) and UV (lower) spectra (MeOH) of compound **B-7**.

Figure S5. ^1H NMR spectrum with water suppression (700 MHz, CD_3OH) of compound **B-7**.

Figure S6. ^1H NMR spectrum (above) and sel-TOCSY spectrum of the impurities (below, transmitter frequency at 4.07 ppm) of compound **B-7**.

Figure S7. ^1H–^1H COSY spectrum of compound **B-7** in CD_3OH.

Figure S8. Superimposed HSQC and HMBC spectra of compound **B-7** in CD_3OH (part-1).

Figure S9. Superimposed HSQC and HMBC spectra of compound **B-7** in CD_3OH (part-2).

Figure S10. ROESY spectrum of compound **B-7** in CD_3OH.

Musellin B (B-9): **page 19–26**

Figure S11. Chemical shifts of compound **B-9**.

Figure S12. HR-ESI-MS spectrum of compound **B-9**.

Figure S13. UV/Vis spectrum (MeCN-H_2O) of compound **B-9**.

Figure S14. Experimental ECD (upper) and UV (lower) spectra (MeOH) of compound **B-9**.

Figure S15. Comparison of calculated ECD (left) and UV (right) spectra of (1S,3S,5S,7S)-**B-9** and (1S,3S,5S,7R)-**B-9**.

Figure S16. ^1H NMR spectrum with water suppression (700 MHz, CD_3OH) of compound **B-9**.

Figure S17. ^1H–^1H COSY spectrum of compound **B-9** in CD_3OH.

Figure S18. Superimposed HSQC and HMBC spectra of compound **B-9** in CD_3OH (part-1).

Figure S19. Superimposed HSQC and HMBC spectra of compound **B-9** in CD_3OH (part-2).

Figure S20. ROESY spectrum of compound **B-9** in CD_3OH.

Musellin C (B-10): **page 27–34**

Figure S21. Chemical shifts of compound **B-10**.

Figure S22. HR-ESI-MS spectrum of compound **B-10**.

Figure S23. UV/Vis spectrum (MeCN-H_2O) of compound **B-10**.

Figure S24. Experimental ECD (upper) and UV (lower) spectra (MeOH) of compound **B-10**.

Figure S25. ^{1}H NMR spectrum with water suppression (700 MHz, CD_3OH) of compound **B-10**.

Figure S26. ^{1}H–^{1}H COSY spectrum of compound **B-10** in CD_3OH.

Figure S27. Superimposed HSQC and HMBC spectra of compound **B-10** in CD_3OH (part-1).

Figure S28. Superimposed HSQC and HMBC spectra of compound **B-10** in CD_3OH (part-2).

Figure S29. ROESY spectrum of compound **B-10** in CD_3OH.

Musaitinerin A (B-16): **page 35–43**

Figure S30. Chemical shifts of compound **B-16**.

Figure S31. HR-ESI-MS spectrum of compound **B-16**.

Figure S32. UV/Vis spectrum (MeCN-H_2O) of compound **B-16**.

Figure S33. Experimental ECD (upper) and UV (lower) spectra (MeOH) of compound **B-16**.

Figure S34. ^{1}H NMR spectrum (700 MHz, CD_3OH) of compound **B-16**.

Figure S35. ^{1}H–^{1}H COSY spectrum of compound **B-16** in CD_3OH.

Figure S36. Superimposed HSQC and HMBC spectra of compound **B-16** in CD_3OH (part-1).

Figure S37. Superimposed HSQC and HMBC spectra of compound **B-16** in CD_3OH (part-2).

Figure S38. Superimposed HSQC and HMBC spectra of compound **B-16** in CD_3OH (part-3).

Figure S39. ROESY spectrum of compound **B-16** in CD_3OH.

Musellin E (B-14): **page 44–52**

Figure S40. Chemical shifts of compound **B-14**.

Figure S41. HR-ESI-MS spectrum of compound **B-14**.

Figure S42. UV/Vis spectrum (MeCN-H_2O) of compound **B-14**.

Figure S43. Experimental ECD (upper) and UV (lower) spectra (MeOH) of compound **B-14**.

Figure S44. ^1H NMR spectrum (700 MHz, CD_3CN) of compound **B-14**.

Figure S45. ^1H–^1H COSY spectrum of compound **B-14** in CD_3CN.

Figure S46. Superimposed HSQC and HMBC spectra of compound **B-14** in CD_3CN (part-1).

Figure S47. Superimposed HSQC and HMBC spectra of compound **B-14** in CD_3CN (part-2).

Figure S48. Superimposed HSQC and HMBC spectra of compound **B-14** in CD_3CN (part-3).

Figure S49. ROESY spectrum of compound **B-14** in CD_3CN.

Musellin D (B-12): **page 53–61**

Figure S50. Chemical shifts of compound **B-12**.

Figure S51. HR-ESI-MS spectrum of compound **B-12**.

Figure S52. UV/Vis spectrum (MeCN-H_2O) of compound **B-12**.

Figure S53. Experimental ECD (upper) and UV (lower) spectra (MeOH) of compound **B-12**.

Figure S54. Comparison of experimental and calculated ECD (left) and UV (right) spectra of compounds **B-12** and **B-12a**.

Figure S55. ^1H NMR spectrum with water suppression (700 MHz, CD_3OH) of compound **B-12**.

Figure S56. ^1H–^1H COSY spectrum of compound **B-12** in CD_3OH.

Figure S57. Superimposed HSQC and HMBC spectra of compound **B-12** in CD_3OH (part-1).

Figure S58. Superimposed HSQC and HMBC spectra of compound **B-12** in CD_3OH (part-2).

Figure S59. Superimposed HSQC and HMBC spectra of compound **B-12** in CD_3OH (part-3).

Figure S60. ROESY spectrum of compound **B-12** in CD_3OH.

Musellin F (B-15): **page 62–75**

Figure S61. Chemical shifts of compound **B-15**.

Figure S62. HR-ESI-MS spectrum of compound **B-15**.

Figure S63. UV/Vis spectrum (MeCN-H_2O) of compound **B-15**.

Figure S64. Experimental ECD (upper) and UV (lower) spectra (MeOH) of compound **B-15**.

Figure S65. Experimental ECD (left) and UV (right) spectra of compounds **B-12** and **B-15**.

Figure S66. Detail of the ^1H NMR spectra (700 MHz, CD_3OH) of compound **B-12** (above) and compound **B-15** (below) (part-1).

Figure S67. Detail of the ^1H NMR spectra (700 MHz, CD_3OH) of compound **B-12** (above) and compound **B-15** (below) (part-2).

Figure S68. Detail of the ^1H NMR spectra (700 MHz, CD_3OH) of compound **B-12** (above) and compound **B-15** (below) (part-3).

Figure S69. ^1H–^1H COSY spectrum of compound **B-15** in CD_3OH (part-1).

Figure S70. ^1H–^1H COSY spectrum of compound **B-15** in CD_3OH (part-2).

Figure S71. ^1H NMR spectrum (above) and sel-TOCSY spectrum (below, transmitter frequency at 2.90 ppm) of compound **B-15**.

Figure S72. ^1H NMR spectrum (above) and sel-TOCSY spectrum (below, transmitter frequency at 1.42 ppm) of compound **B-15**.

Figure S73. Superimposed HSQC and HMBC spectra of partial compound **B-15** in CD_3OH (part-1).

Figure S74. Superimposed HSQC and HMBC spectra of partial compound **B-15** in CD_3OH (part-2).

Figure S75. Superimposed HSQC and HMBC spectra of partial compound **B-15** in CD_3OH. (part-3).

Figure S76. ROESY spectrum of compound **B-15** in CD_3OH.

Dimeric phenylphenalenone (M-6): **page 76–83**

Figure S77. Chemical shifts of compound **M-6**.

Figure S78. HR-ESI-MS spectrum of compound **M-6**.

Figure S79. UV/Vis spectrum (MeCN-H_2O) of compound **M-6**.

Figure S80. ^{1}H NMR spectrum (700 MHz, acetone-d_6) of compound **M-6**.

Figure S81. ^{1}H–^{1}H COSY spectrum of compound **M-6** in acetone-d_6.

Figure S82. Superimposed HSQC and HMBC spectra of compound **M-6** in acetone-d_6 (part-1).

Figure S83. Superimposed HSQC and HMBC spectra of compound **M-6** in acetone-d_6 (part-2).

Figure S84. HMBC spectrum (J_{CH} = 1 Hz, acetone-d_6) and DEPTQ spectrum (175 MHz, acetone-d_6, part-1) of compound **M-6**.

Figure S85. DEPTQ spectrum (175 MHz, acetone-d_6) of compound **M-6** (part-2).

Dimeric phenylphenalenone (M-4): **page 84–91**

Figure S86. Chemical shifts of compound **M-4**.

Figure S87. HR-ESI-MS spectrum of compound **M-4**.

Figure S88. UV/Vis spectrum (MeCN-H_2O) of compound **M-4**.

Figure S89. ^{1}H NMR spectrum (700 MHz, CD_3OH) of compound **M-4**.

Figure S90. ^{1}H–^{1}H COSY spectrum of compound **M-4** in CD_3OH.

Figure S91. Superimposed HSQC and HMBC spectra of compound **M-4** in CD_3OH (part-1).

Figure S92. Superimposed HSQC and HMBC spectra of compound **M-4** in CD_3OH (part-2).

Figure S93. DEPTQ spectrum (175 MHz, CD_3OH) of compound **M-4** (part-1).

Figure S94. DEPTQ spectrum (175 MHz, CD_3OH) of compound **M-4** (part-2).

Known compounds

Vanillic acid glucosyl ester (**B-1**):

UV/Vis (MeCN-H_2O) λ_{max} 204, 222, 266, 296 nm; ^1H NMR (CD_3OH, 700 MHz), δ 7.64 (IH, d, J = 1.5 Hz, H-2), 6.88 (1H, d, J = 8.3 Hz, H-5), 7.66 (1H, dd, J = 8.3, 1.5 Hz, H-6), 3.93 (3H, s, OCH_3), and 5.71 (1H, d, J = 7.6 Hz, glucose anomeric proton); ^{13}C NMR (CD_3OH, 175 MHz), δ 121.8 (C-1), 114.0 (C-2), 148.9 (C-3), 153.6 (C-4), 116.2 (C-5), 125.8 (C-6), 166.9 (C-7), 56.6 (OCH_3), 96.2 (Glc-1), 74.4 (Glc-2), 79.1 (Glc-3), 71.4 (Glc-4), 78.5 (Glc-5), and 62.7 (Glc-6); (-)-HRESIMS *m/z* 329.0874 $[M – H]^-$ (calcd for $C_{14}H_{17}O_9$, 329.0878).

Syringic acid glucosyl ester (**B-2**):

UV/Vis (MeCN-H_2O) λ_{max} 218, 284 nm; ^1H NMR (CD_3CN, 700 MHz), δ 7.39 (2H, s, H-2/H-6), 3.89 (6H, s, 2x OCH_3), and 5.69 (1H, d, J = 7.7 Hz, glucose anomeric proton); ^{13}C NMR (CD_3CN, 175 MHz), δ 120.2 (C-1), 108.2 (C-2/C-6), 148.5 (C-3/C-5), 142.3 (C-4), 166.3 (C-7), 56.4 (2x OCH_3), 95.9 (Glc-1), 73.8 (Glc-2), 78.5 (Glc-3), 70.8 (Glc-4), 77.9 (Glc-5), and 62.0 (Glc-6); (-)-HRESIMS *m/z* 359.0991 $[M – H]^-$ (calcd for $C_{15}H_{19}O_{10}$, 359.0984).

2-methoxy-9-(3',4'-dihydroxyphenyl)-1*H*-phenalen-1-one (**B-8**/**M-3**):

UV/Vis (MeCN-H_2O) λ_{max} 218, 269, 366, 413 nm; ^1H NMR (CD_3OH, 700 MHz), δ 7.17 (1H, s, H-3), 7.85 (1H, d, J = 7.3 Hz, H-4), 7.63 (1H, dd, J = 8.4, 7.3 Hz, H-5), 8.00 (1H, d, J = 8.4 Hz, H-6), 8.26 (1H, d, J = 8.3 Hz, H-7), 7.60 (1H, d, J = 8.3 Hz, H-8), 6.80 (1H, d, J = 2.1 Hz, H-2'), 6.83 (1H, d, J = 8.0 Hz, H-5'), 6.69 (1H, dd, J = 8.0, 2.1, H-6'), and 3.89 (3H, s, OCH_3); ^{13}C NMR

(CD_3OH, 175 MHz), δ 181.4 (C-1), 154.2 (C-2), 113.2 (C-3), 126.2 (C-3a), 131.1 (C-4), 127.5 (C-5), 130.4 (C-6), 132.2 (C-6a), 135.6 (C-7), 132.6 (C-8), 150.2 (C-9), 126.2 (C-9a), 129.3 (C-9b), 135.8 (C-1'), 116.4 (C-2'), 146.0 (C-3'), 146.0 (C-4'), 116.1 (C-5'), 120.5 (C-6'), and 55.8 (C-OCH_3); (-)-HRESIMS *m/z* 317.0829 $[M – H]^-$ (calcd for $C_{20}H_{13}O_4$, 317.0819).

2-methoxy-9-(4'-hydroxyphenyl)-1*H*-phenalen-1-one (**B-11**/**M-5**):

UV/Vis ($MeCN-H_2O$) λ_{max} 218, 269, 365, 413 nm; ^1H NMR (CD_3OH, 700 MHz), δ 7.17 (H, s, H-3), 7.85 (1H, d, J = 7.1 Hz, H-4), 7.63 (1H, dd, J = 8.1, 7.1 Hz, H-5), 8.01 (1H, d, J = 8.1 Hz, H-6), 8.28 (1H, d, J = 8.3 Hz, H-7), 7.60 (1H, d, J = 8.3 Hz, H-8), 7.20 (2H, d, J = 8.5 Hz, H-2'/H-6'), 6.85 (2H, d, J = 8.5 Hz, H-3'/H-5'), and 3.89 (3H, s, OCH_3); ^{13}C NMR (CD_3OH, 175 MHz), δ 181.4 (C-1), 154.2 (C-2), 113.2 (C-3), 126.1 (C-3a), 131.1 (C-4), 127.5 (C-5), 130.4 (C-6), 132.2 (C-6a), 135.6 (C-7), 132.6 (C-8), 150.0 (C-9), 126.2 (C-9a), 129.1 (C-9b), 134.7 (C-1'), 130.3 (C-2'/C-6'), 115.9 (C-3'/C-5'), 157.9 (C-4'), and 55.8 (C-OCH_3); (-)-HRESIMS *m/z* 301.0881 $[M – H]^-$ (calcd for $C_{20}H_{13}O_3$, 301.0870).

2-hydroxy-9-(4'-hydroxyphenyl)-1*H*-phenalen-1-one (hydroxyanigorufone**, B-13**/**M-7**):

UV/Vis ($MeCN-H_2O$) λ_{max} 221, 271, 365, 413 nm; ^1H NMR (acetone-d_6, 700 MHz), δ 7.15 (H, s, H-3), 7.84 (1H, d, J = 7.1 Hz, H-4), 7.64 (1H, dd, J = 8.1, 7.1 Hz, H-5), 8.03 (1H, d, J = 8.1 Hz, H-6), 8.32 (1H, d, J = 8.3 Hz, H-7), 7.60 (1H, d, J = 8.3 Hz, H-8), 7.23 (2H, d, J = 8.5 Hz, H-2'/H-6'), and 6.90 (2H, d, J = 8.5 Hz, H-3'/H-5'); ^{13}C NMR (acetone-d_6, 175 MHz), δ 180.7 (C-1), 151.4 (C-2), 112.8 (C-3), 124.6 (C-3a), 131.0 (C-4), 127.5 (C-5), 130.2 (C-6), 132.2 (C-6a), 135.9 (C-7), 132.6 (C-8), 150.0 (C-9), 126.0 (C-9a), 126.0 (C-9b), 134.7 (C-1'), 130.5 (C-2'/C-6'), 115.9 (C-3'/C-5'), and 157.9 (C-4'); (-)-HRESIMS *m/z* 287.0718 $[M – H]^-$ (calcd for $C_{19}H_{11}O_3$, 287.0714).

(4*E*,6*E*)-1-(3',4'-dihydroxyphenyl)-7-(4''-hydroxyphenyl)-hepta-4,6-dien-3-one (**M-1**):

UV/Vis ($MeCN-H_2O$) λ_{max} 194, 224, 364 nm; ^1H NMR (acetone-d_6, 700 MHz), δ 7.01 (1H, d, J = 15.5 Hz, H-1), 6.89 (1H, dd, J = 15.5, 10.5 Hz, H-2), 7.38 (1H, dd, J = 15.5, 10.5 Hz, H-3), 6.25

(1H, d, J = 15.5 Hz, H-4), 2.86 (2H, t, J = 7.9 Hz, H-6), 2.76 (2H, t, J = 7.9 Hz, H-7), 6.73 (1H, d, J = 2.1 Hz, H-2’), 6.72 (1H, d, J = 8.0 Hz, H-5’), 6.57 (1H, dd, J = 8.0, 2.1 Hz, H-6’), 7.44 (2H, d, J = 8.8 Hz, H-2’’/H-6’’), and 6.86 (2H, d, J = 8.8 Hz, H-3’’/H-5’’); ^{13}C NMR (acetone-d_6, 175 MHz), δ 142.2 (C-1), 125.0 (C-2), 144.1 (C-3), 129.3 (C-4), 199.4 (C-5), 42.8 (C-6), 30.3 (C-7), 134.2(C-1’), 116.3 (C-2’), 145.8 (C-3’), 143.7 (C-4’), 115.9 (C-5’), 120.4 (C-6’), 129.0 (C-1’’), 129.8 (C-2’’/C-6’’), 116.6 (C-3’’/C-5’’), and 159.5 (C-4’’); (+)-HRESIMS *m/z* 311.1264 $[M + H]^+$ (calcd for $C_{19}H_{19}O_4$, 311.1278).

2-(4’-hydroxyphenyl)-1,8-naphthalic anhydride (**M-2**):

UV/Vis (MeCN-H_2O) λ_{max} 238, 341, 375 nm; ^{1}H NMR (acetone-d_6, 700 MHz), δ 7.74 (1H, d, J = 8.6 Hz, H-3), 8.48 (1H, d, J = 8.6 Hz, H-4), 8.54 (1H, dd, J = 8.1, 1.2 Hz, H-5), 7.93 (1H, dd, J = 8.1, 7.3 Hz, H-6), 8.62 (1H, dd, J = 7.3, 1.2 Hz, H-7), 7.39 (2H, d, J = 8.7 Hz, H-2’/H-6’), and 6.95 (2H, d, J = 8.7 Hz, H-3’/H-5’); ^{13}C NMR (acetone-d_6, 175 MHz), δ 116.5 (C-1), 150.8 (C-2), 133.0(C-3), 135.3 (C-4), 132.2 (C-4a), 136.5 (C-5), 127.9 (C-6), 133.9 (C-7), 120.5 (C-8), 132.4 (C-8a), 160.0 (C-9), 162.0 (C-10), 132.9 (C-1’), 131.4 (C-2’/C-6’), 116.3 (C-3’/C-5’), 158.7 (C-4’); (+)-HRESIMS *m/z* 291.0679 $[M + H]^+$ (calcd for $C_{18}H_{11}O_4$, 291.0652).

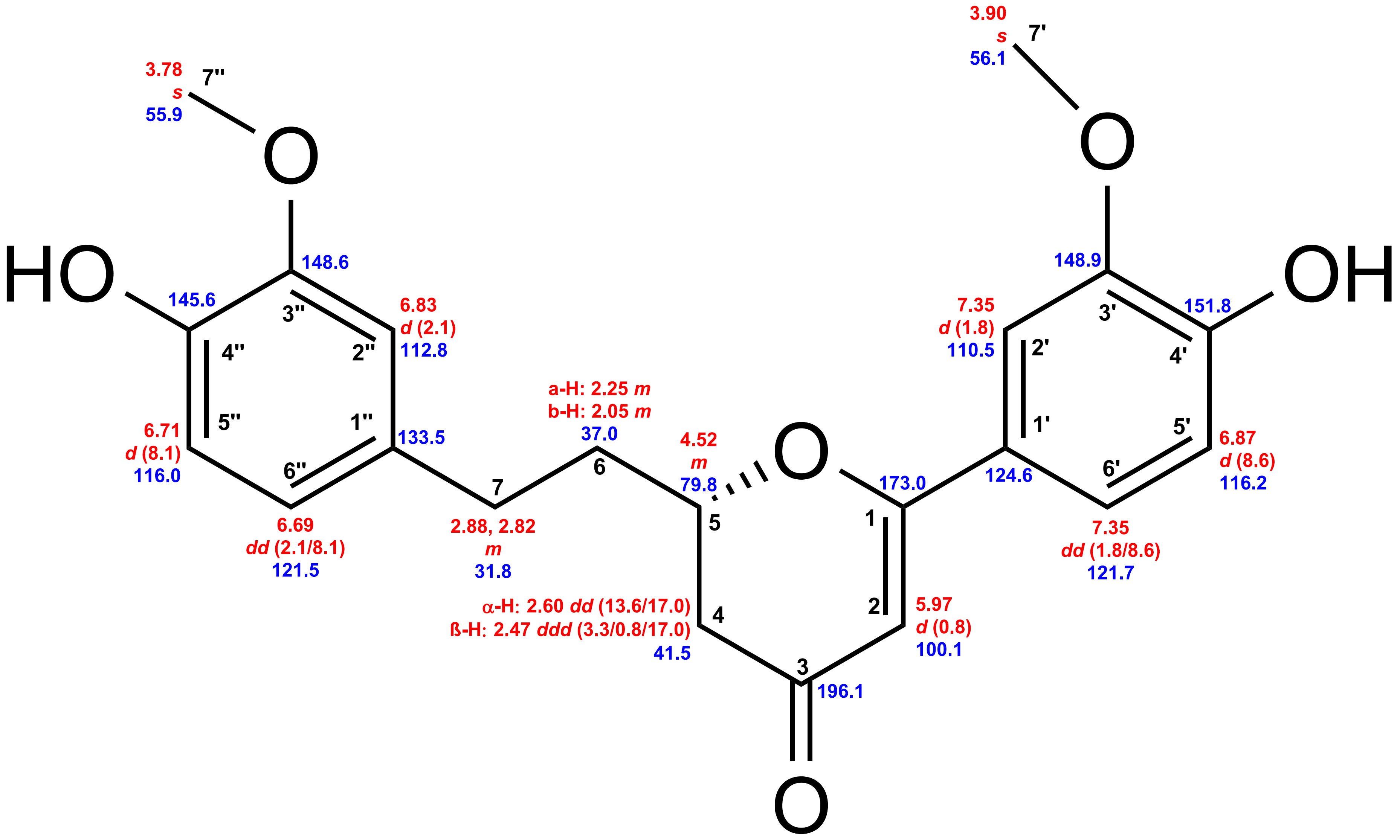

Figure S1. Chemical shifts of compound **B-7**. Red: ^{1}H chemical shifts (δ ppm, *mult*., $^{3}J_{HH}$ in Hz). Blue: ^{13}C chemical shifts (δ ppm).

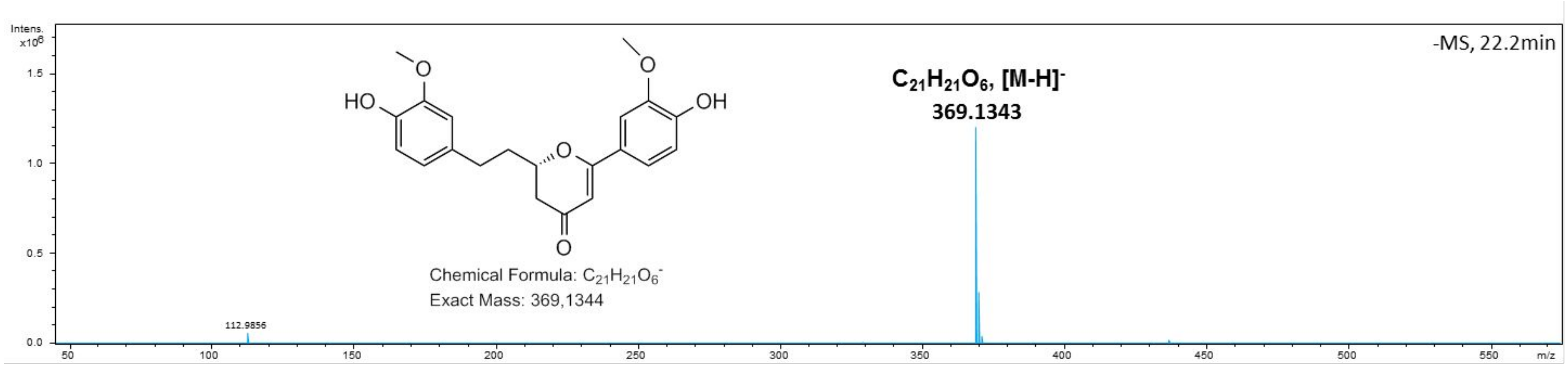

Figure S2. HR-ESI-MS spectrum of compound **B-7**.

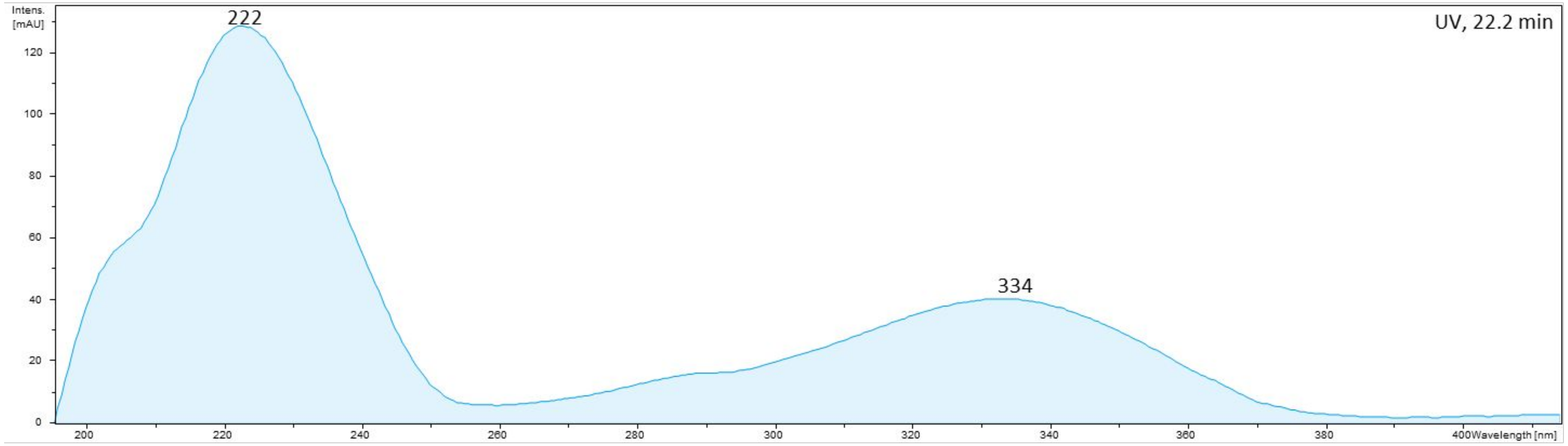

Figure S3. UV/Vis spectrum (MeCN-H_2O) of compound **B-7**.

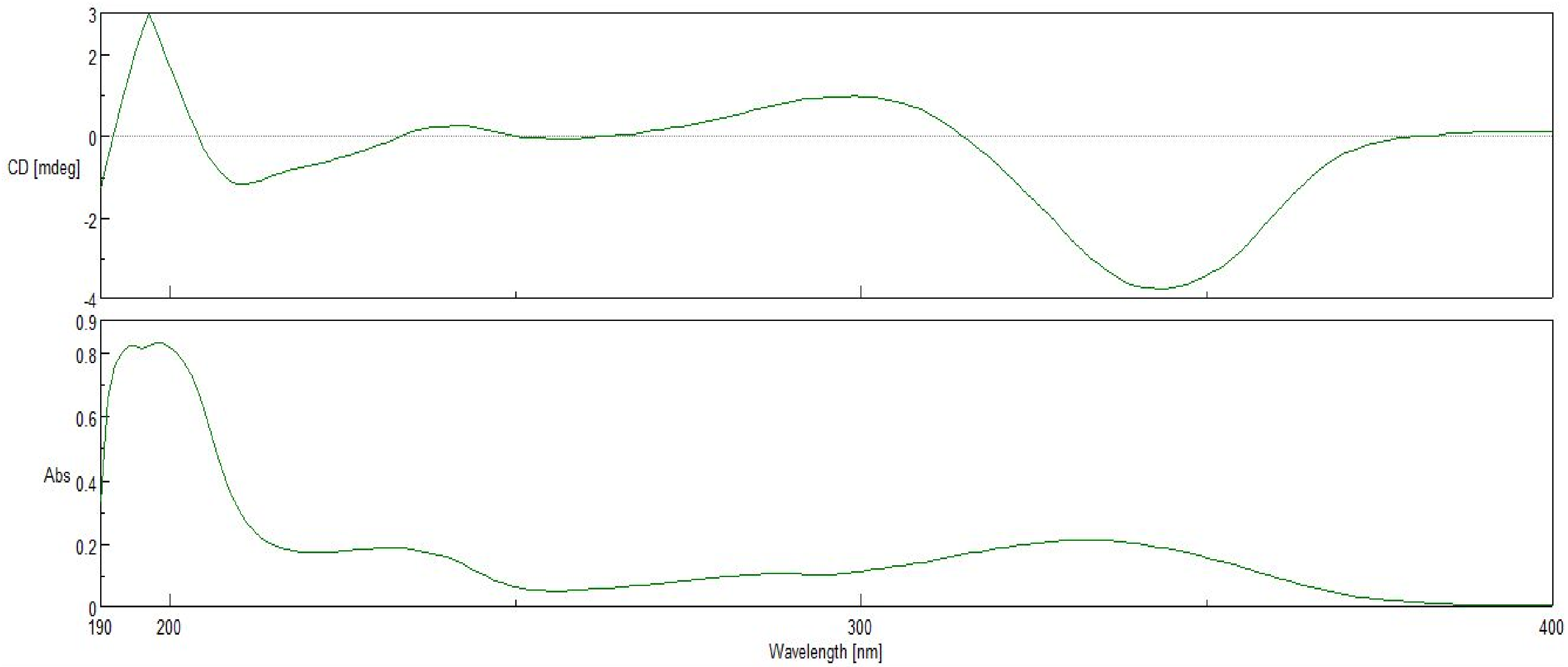

Figure S4. Experimental ECD (upper) and UV (lower) spectra (MeOH) of compound **B-7**.

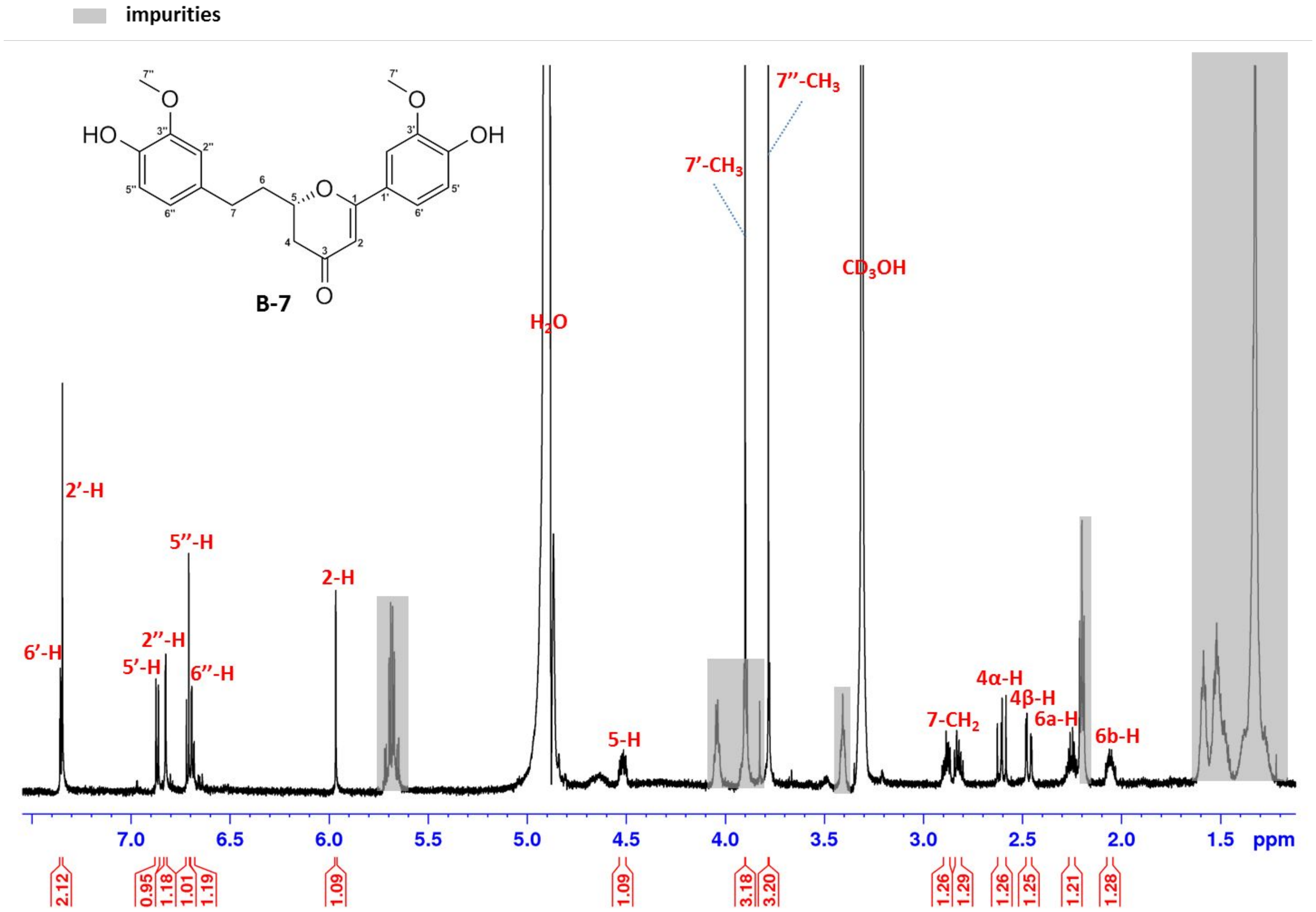

Figure S5. ^{1}H NMR spectrum with water suppression (700 MHz, CD_3OH) of compound **B-7**.

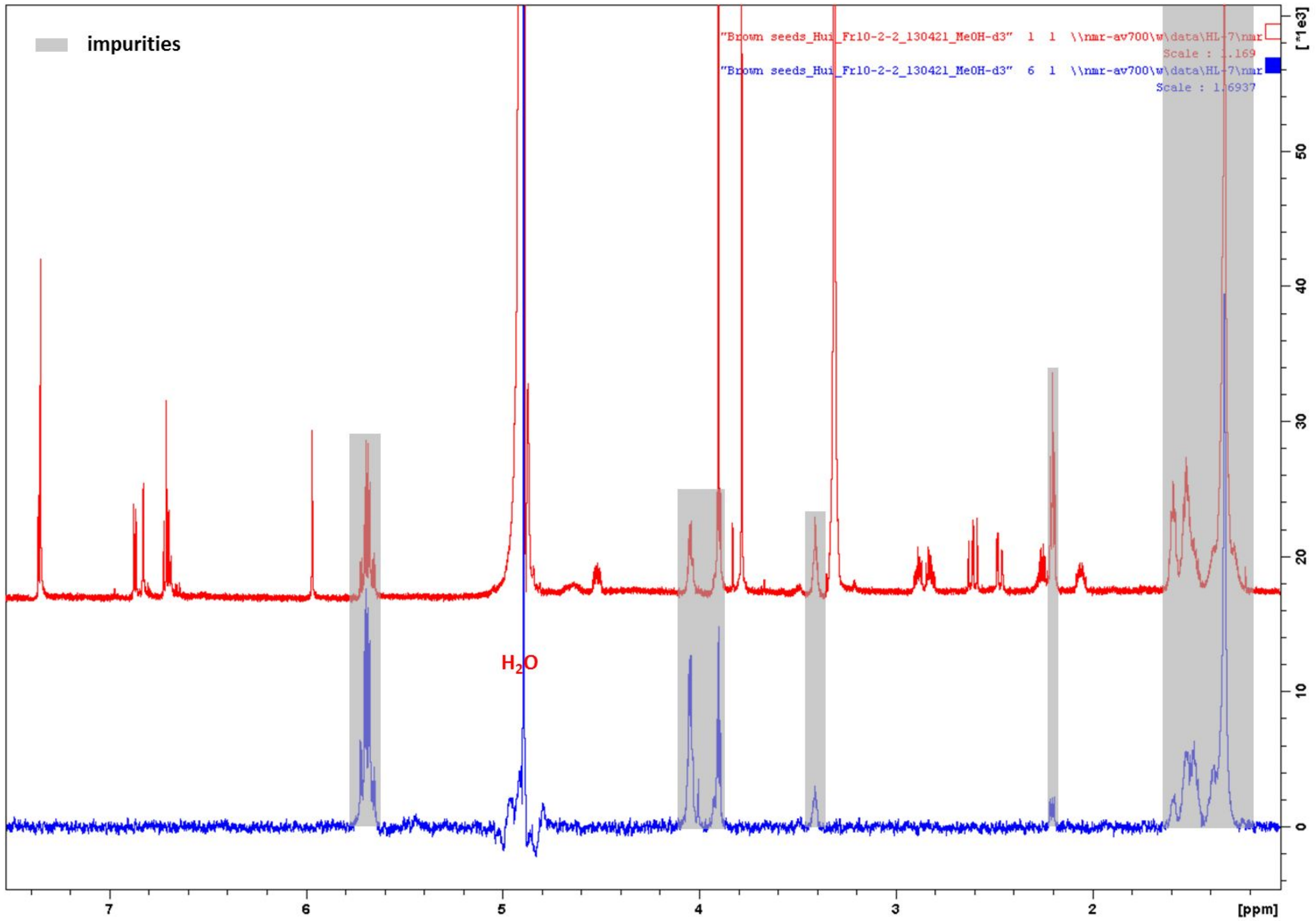

Figure S6. ^{1}H NMR spectrum (above) and sel-TOCSY spectrum of the impurities (below, transmitter frequency at 4.04 ppm) of compound **B-7**.

(in CD_3OH)

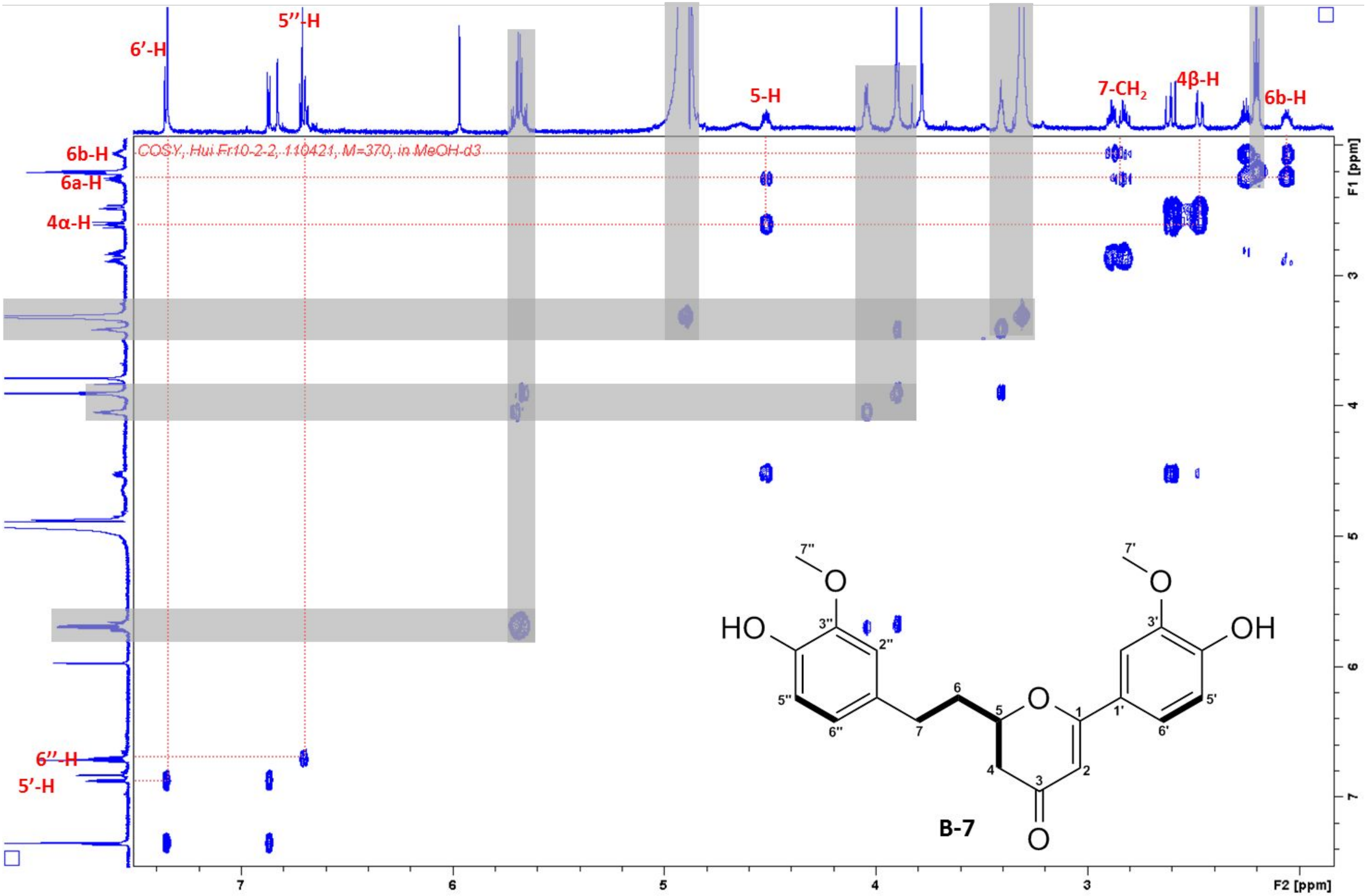

Figure S7. $^{1}H–^{1}H$ COSY spectrum of compound **B-7** in CD_3OH.

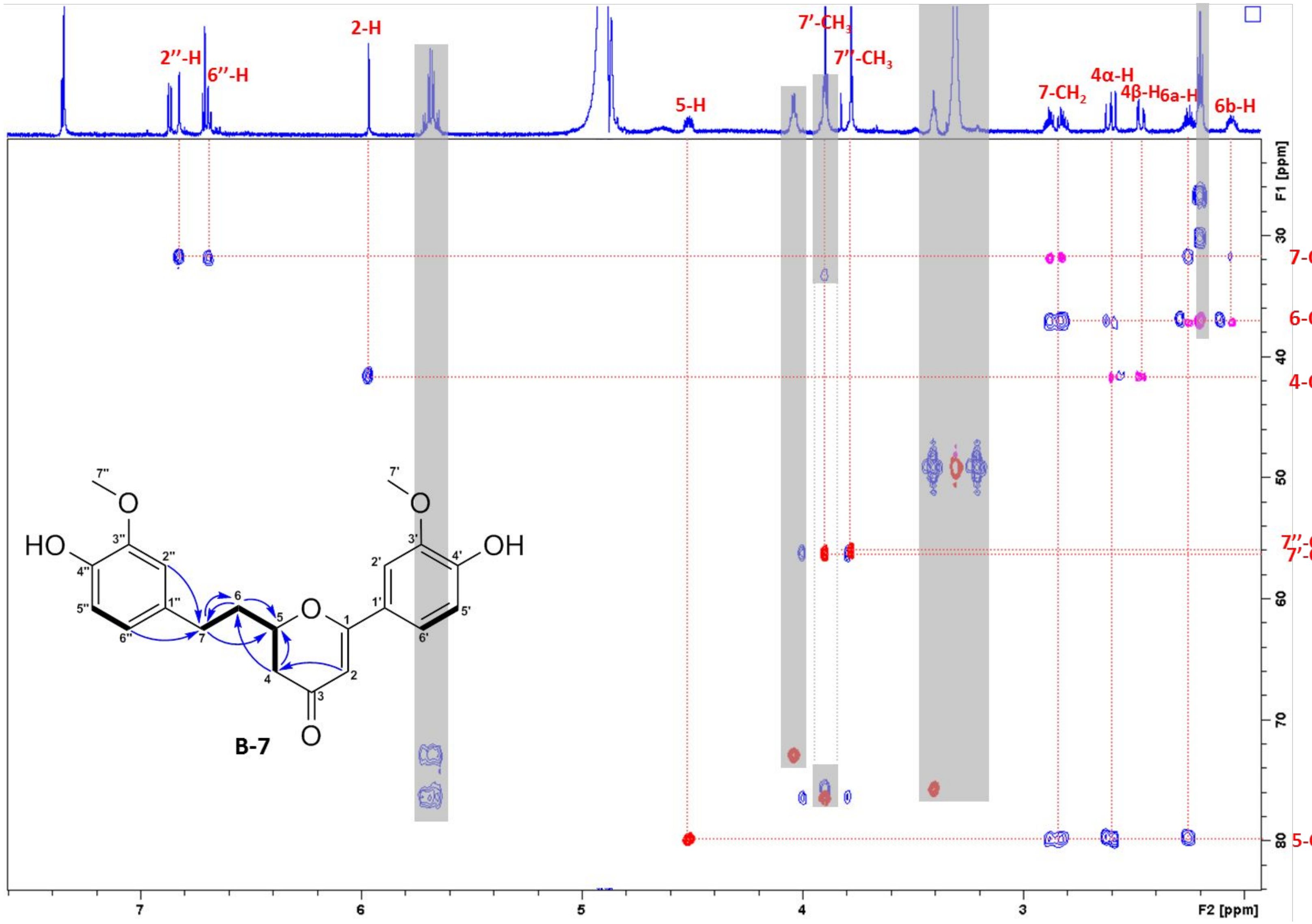

Figure S8. Superimposed HSQC and HMBC spectra of compound **B-7** in CD_3OH (part-1).

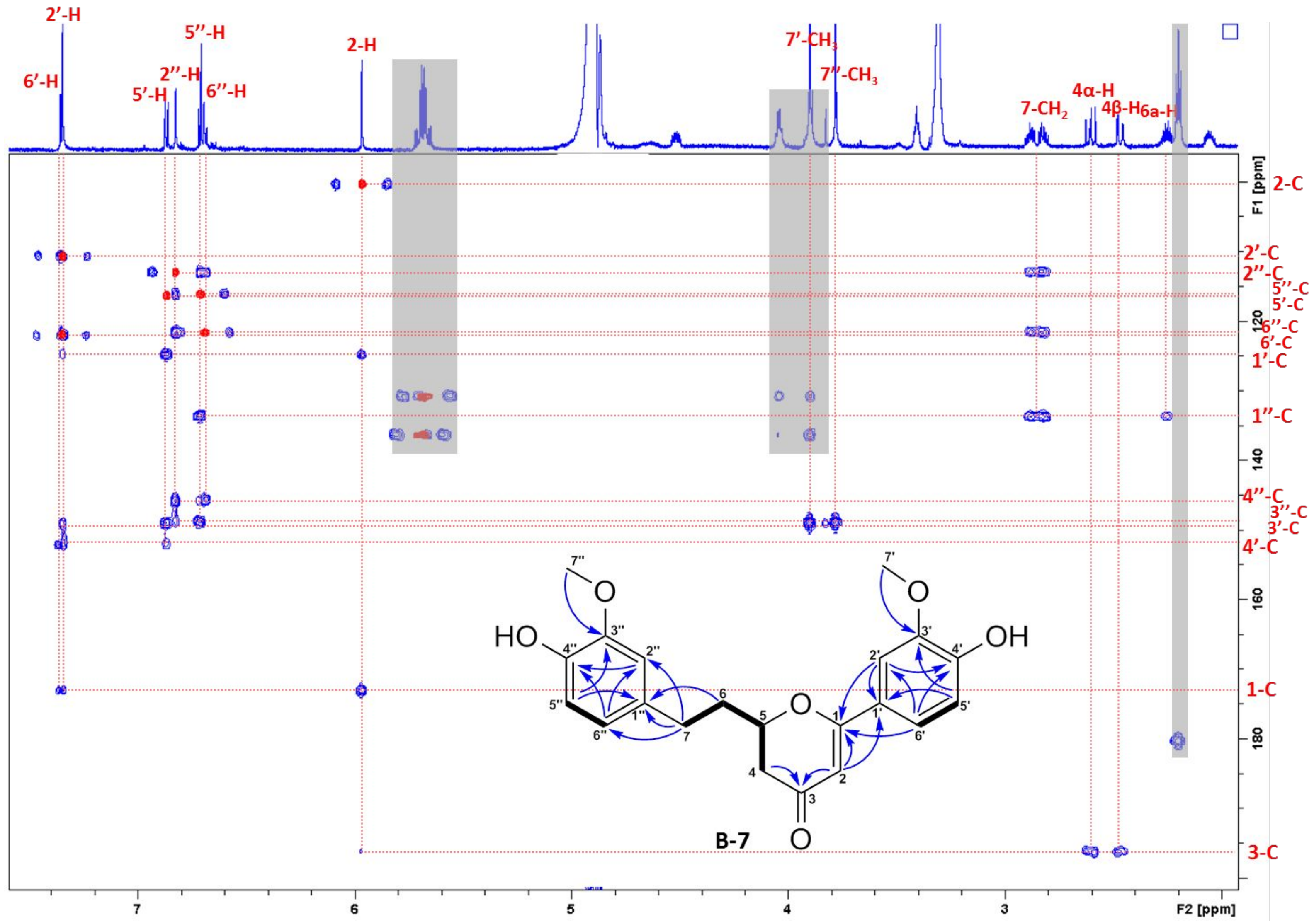

Figure S9. Superimposed HSQC and HMBC spectra of compound **B-7** in CD_3OH (part-2).

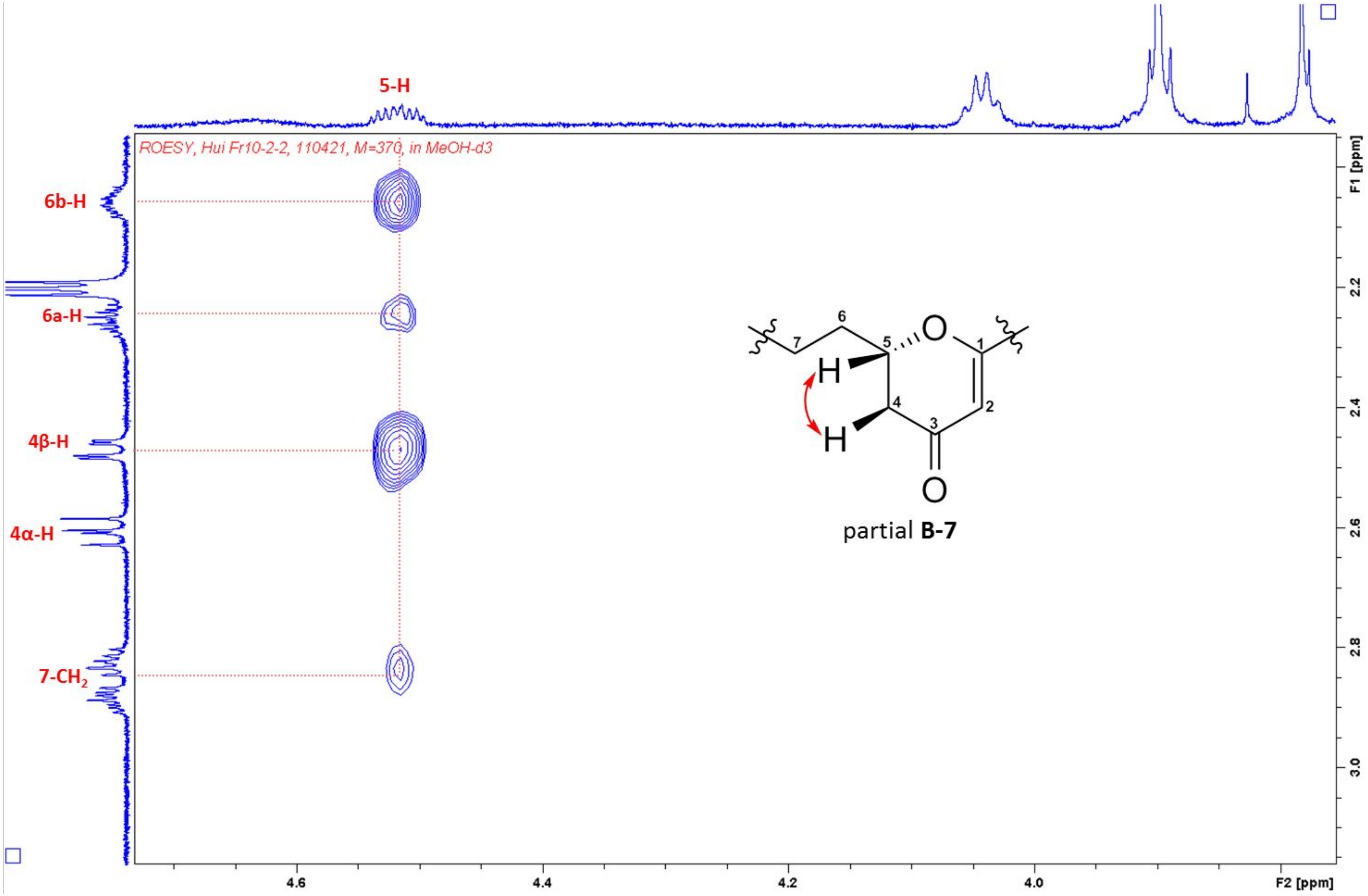

Figure S10. ROESY spectrum of compound **B-7** in CD_3OH.

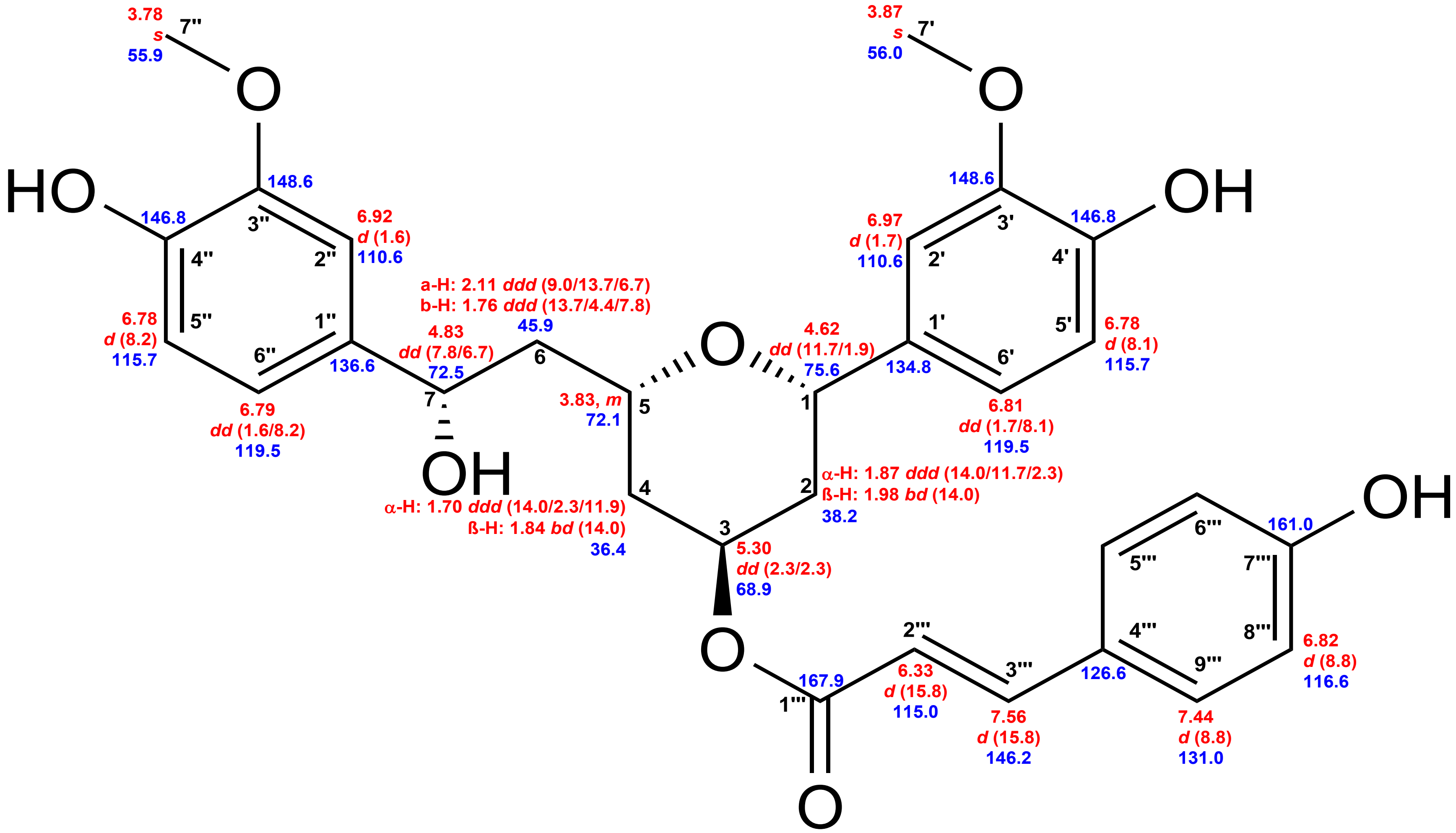

Figure S11. Chemical shifts of compound **B-9**. Red: [1]H chemical shifts (δ ppm, *mult*., $^{3}J_{HH}$ in Hz). Blue: [13]C chemical shifts (δ ppm).

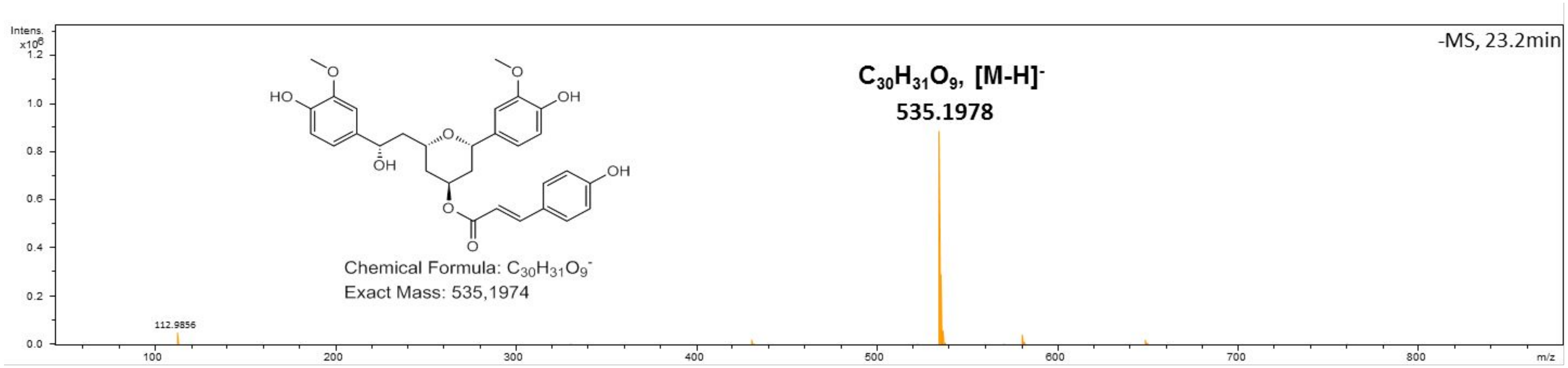

Figure S12. HR-ESI-MS spectrum of compound **B-9**.

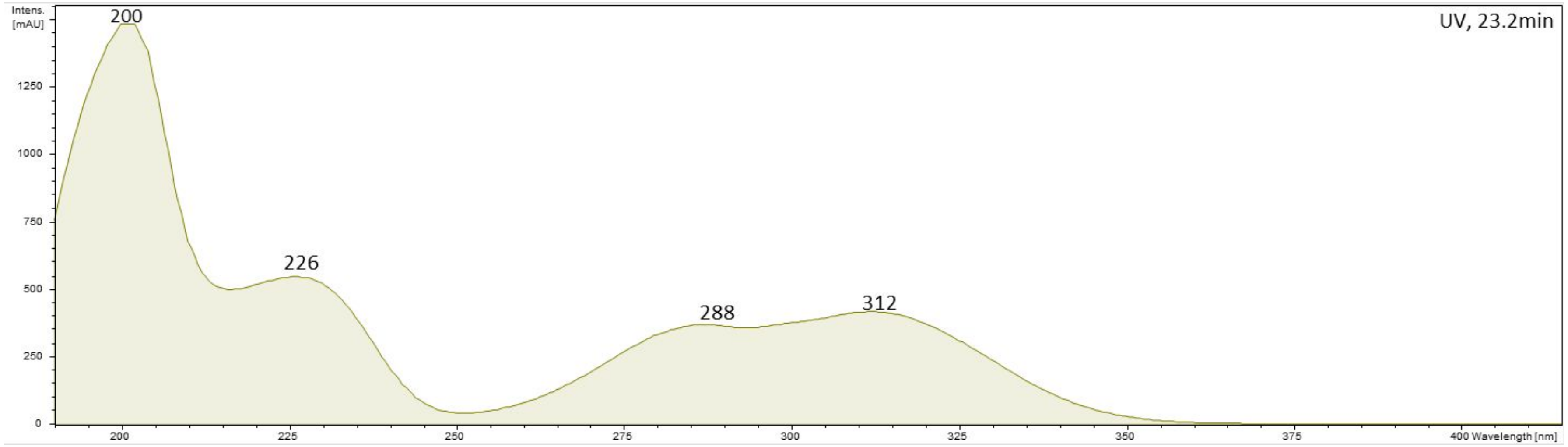

Figure S13. UV/Vis spectrum (MeCN-H_2O) of compound **B-9**.

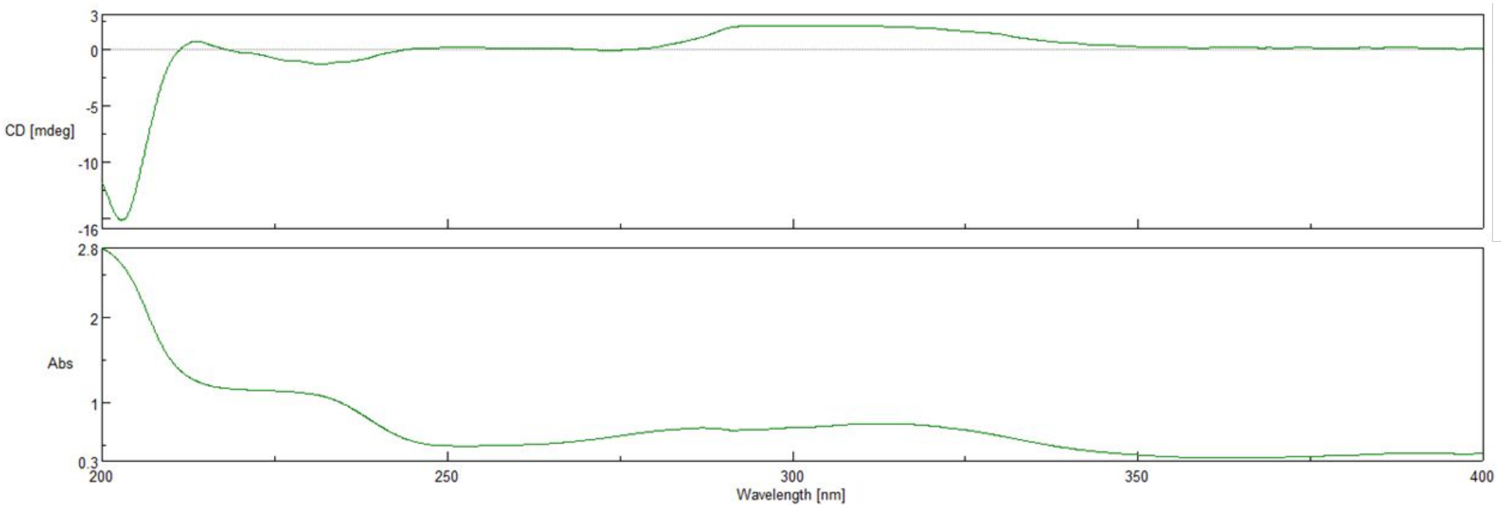

Figure S14. Experimental ECD (upper) and UV (lower) spectra (MeOH) of compound **B-9**.

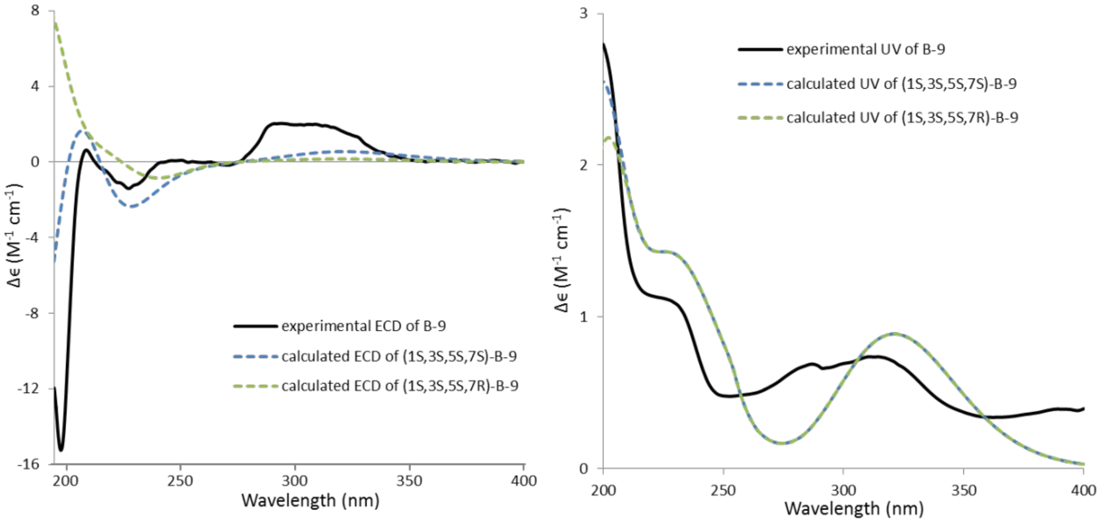

Figure S15. Comparison of calculated ECD (left) and UV (right) spectra of (1S,3S,5S,7S)-**B-9** and (1S,3S,5S,7R)-**B-9**.

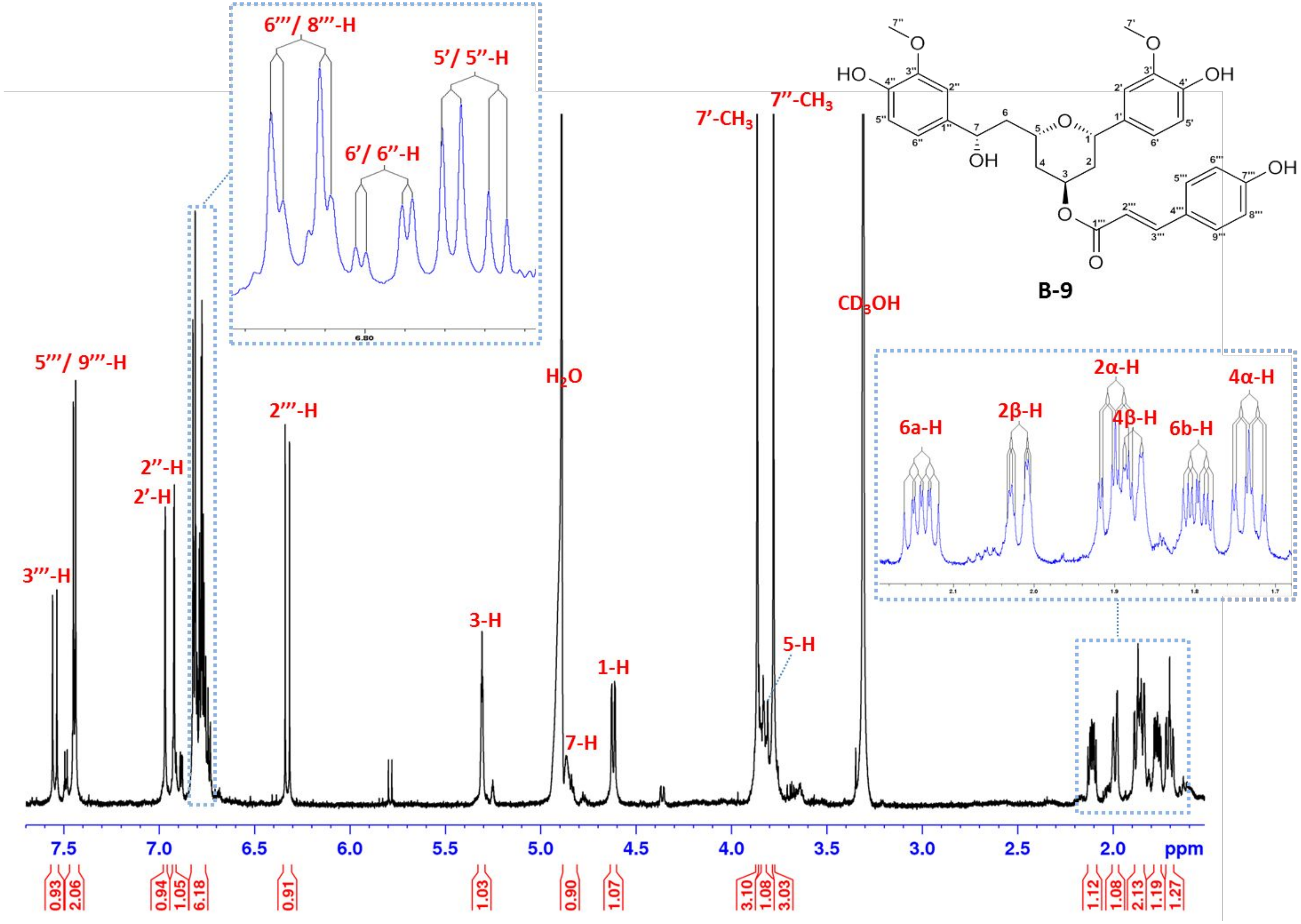

Figure S16. ^{1}H NMR spectrum with water suppression (700 MHz, CD_3OH) of compound **B-9**.

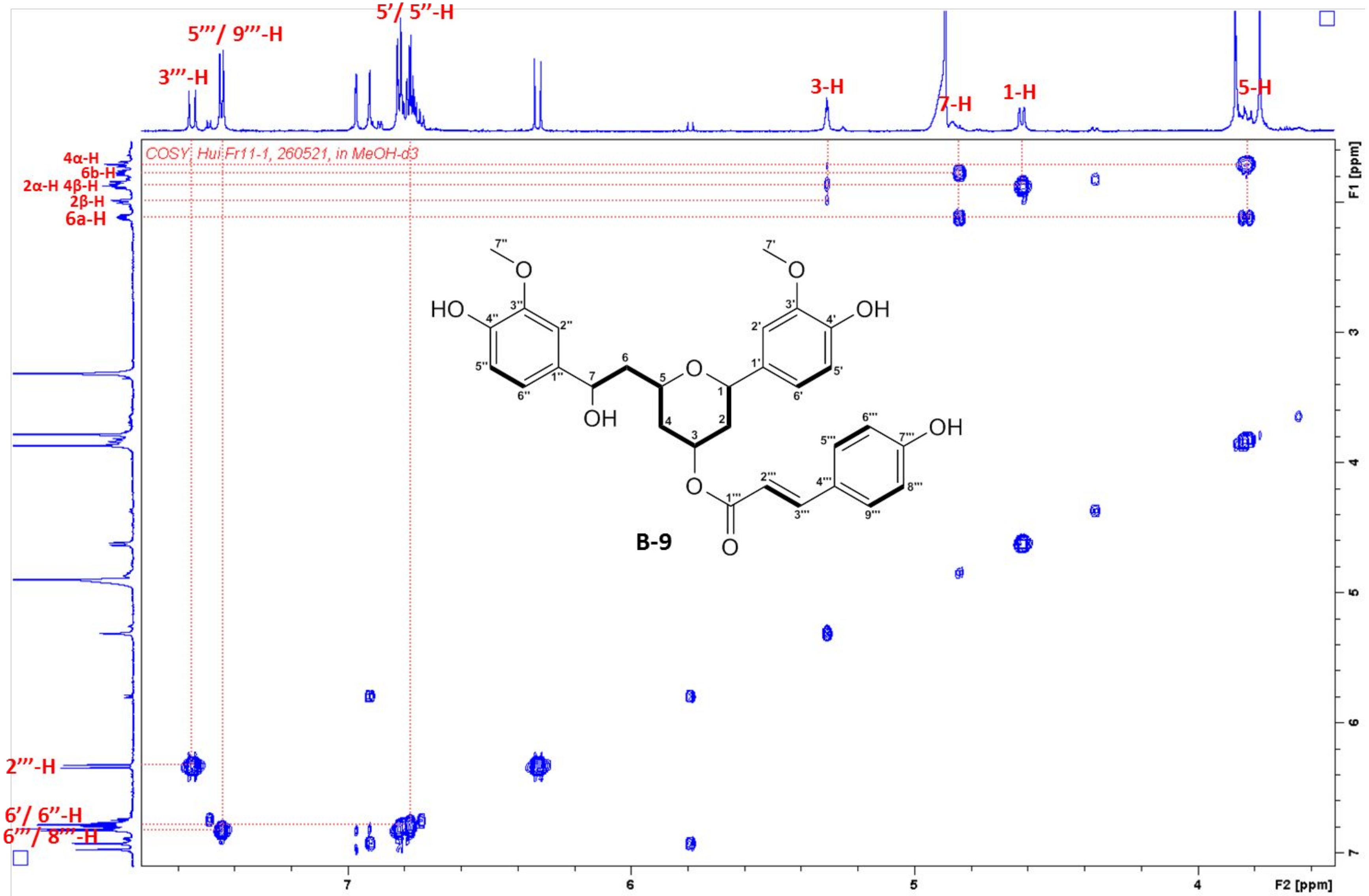

Figure S17. ^{1}H–^{1}H COSY spectrum of compound **B-9** in CD_3OH.

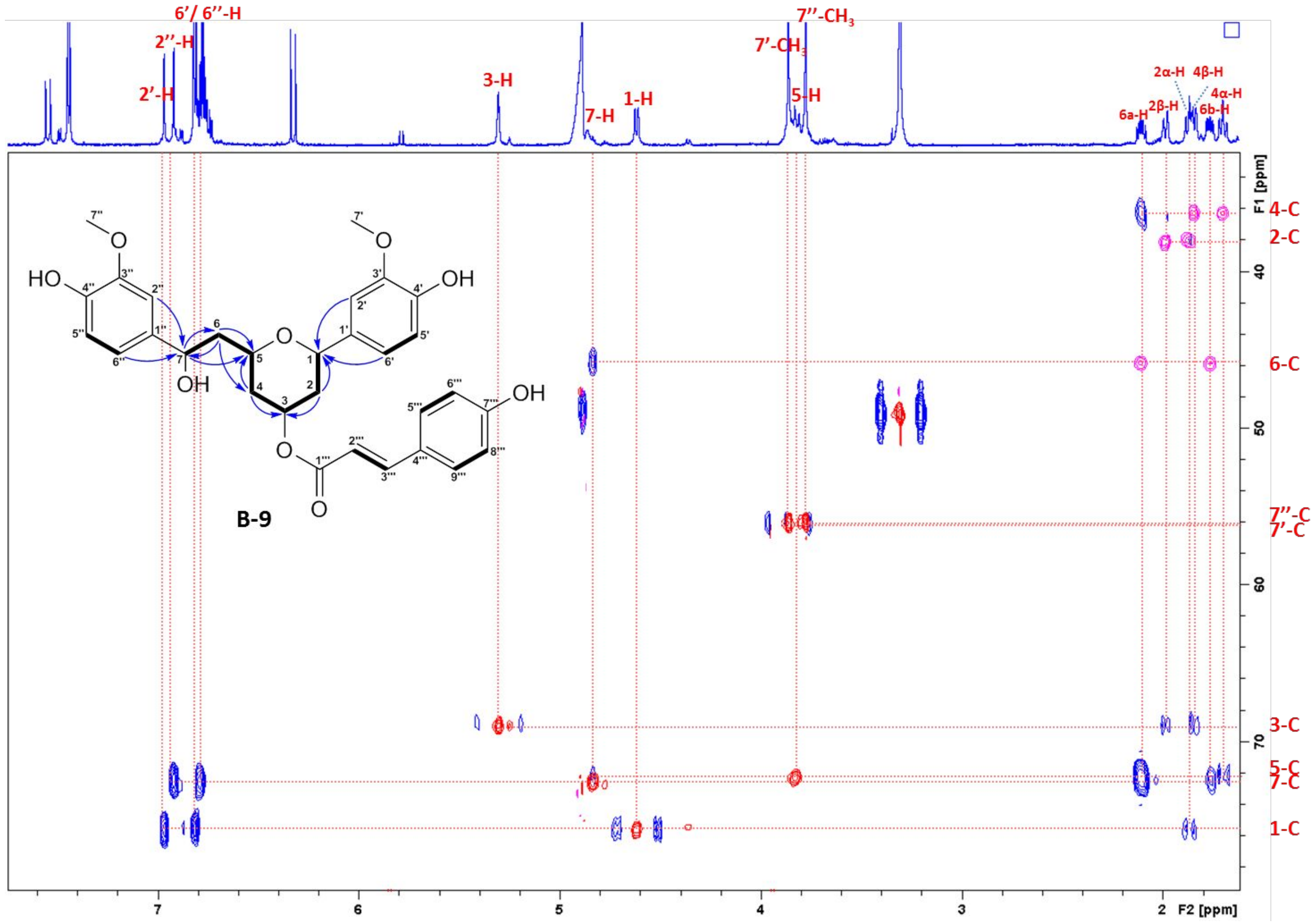

Figure S18. Superimposed HSQC and HMBC spectra of compound **B-9** in CD_3OH (part-1).

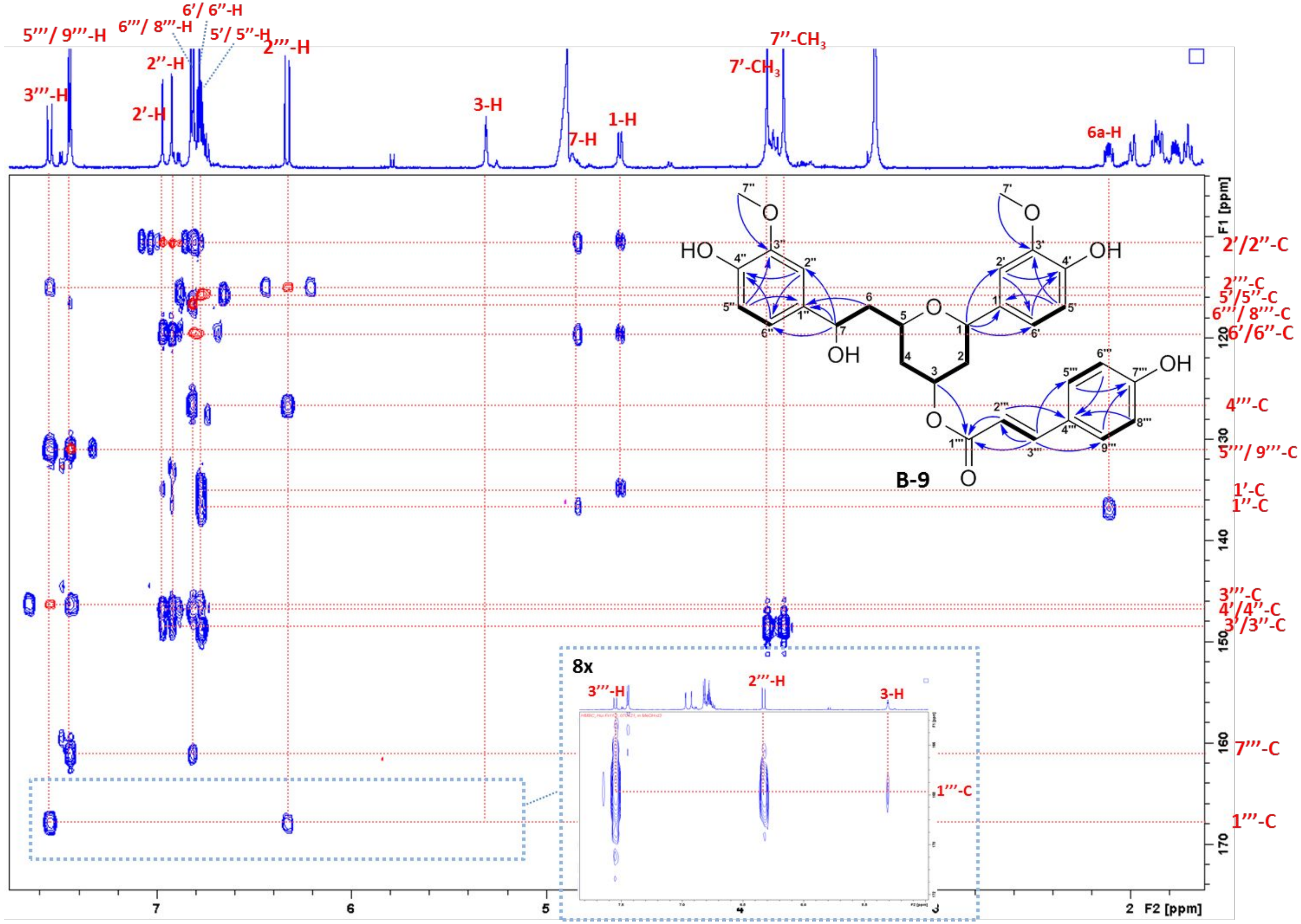

Figure S19. Superimposed HSQC and HMBC spectra of compound **B-9** in CD_3OH (part-2).

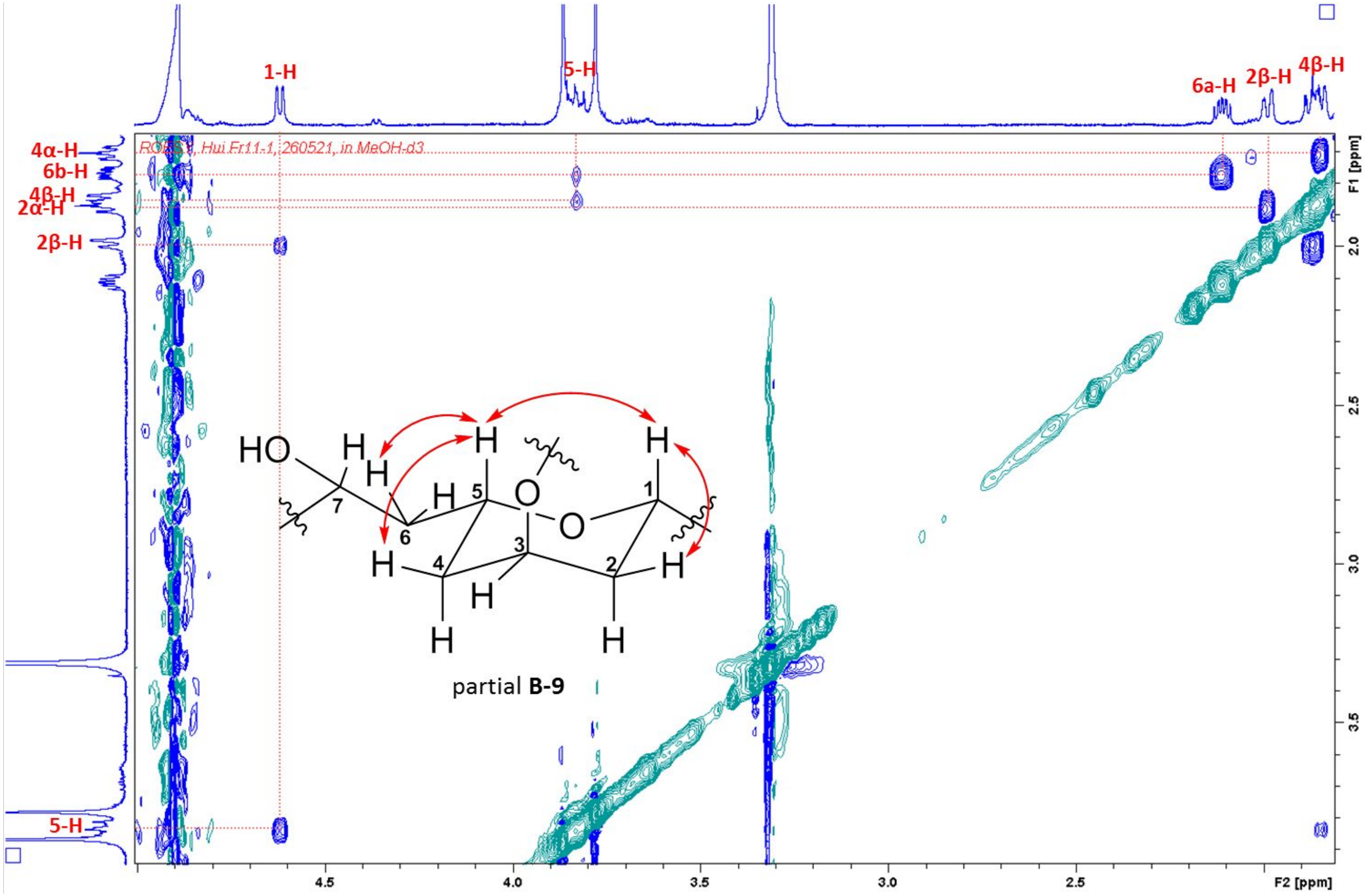

Figure S20. ROESY spectrum of compound **B-9** in CD_3OH.

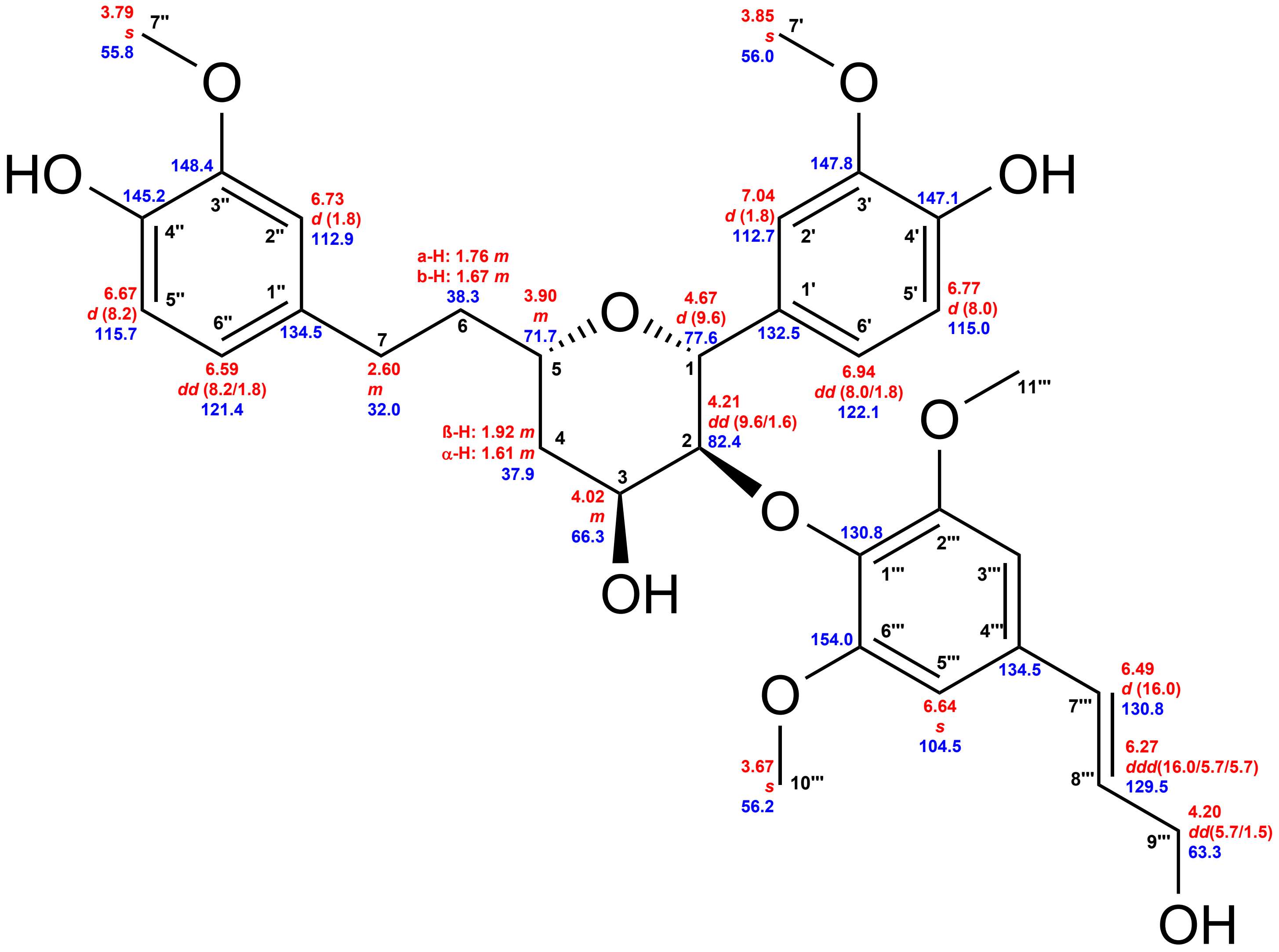

Figure S21. Chemical shifts of compound **B-10**.

Red: [1]H chemical shifts (δ ppm, *mult*., $^{3}J_{HH}$ in Hz). Blue: [13]C chemical shifts (δ ppm).

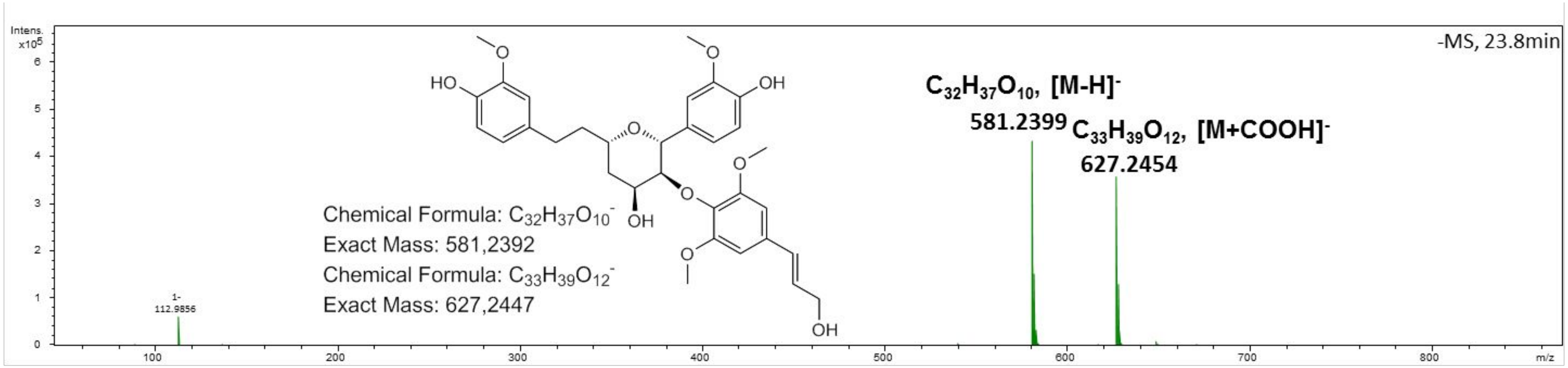

Figure S22. HR-ESI-MS spectrum of compound **B-10**.

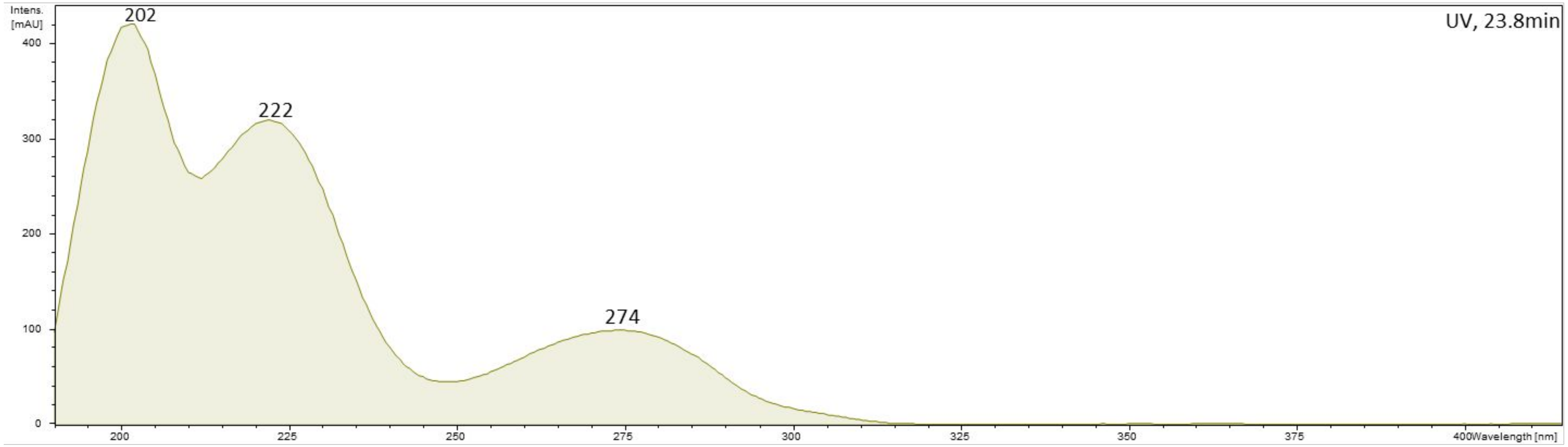

Figure S23. UV/Vis spectrum (MeCN-H_2O) of compound **B-10**.

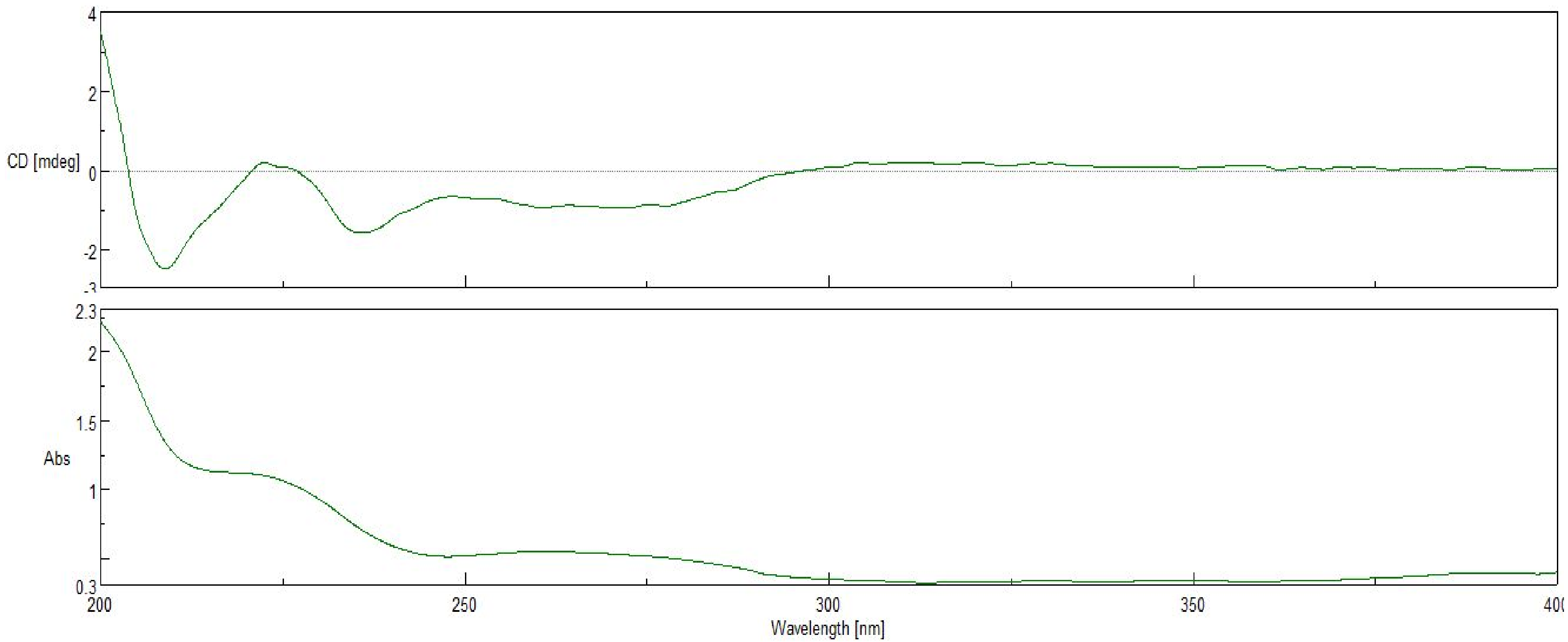

Figure S24. Experimental ECD (upper) and UV (lower) spectra (MeOH) of compound **B-10**.

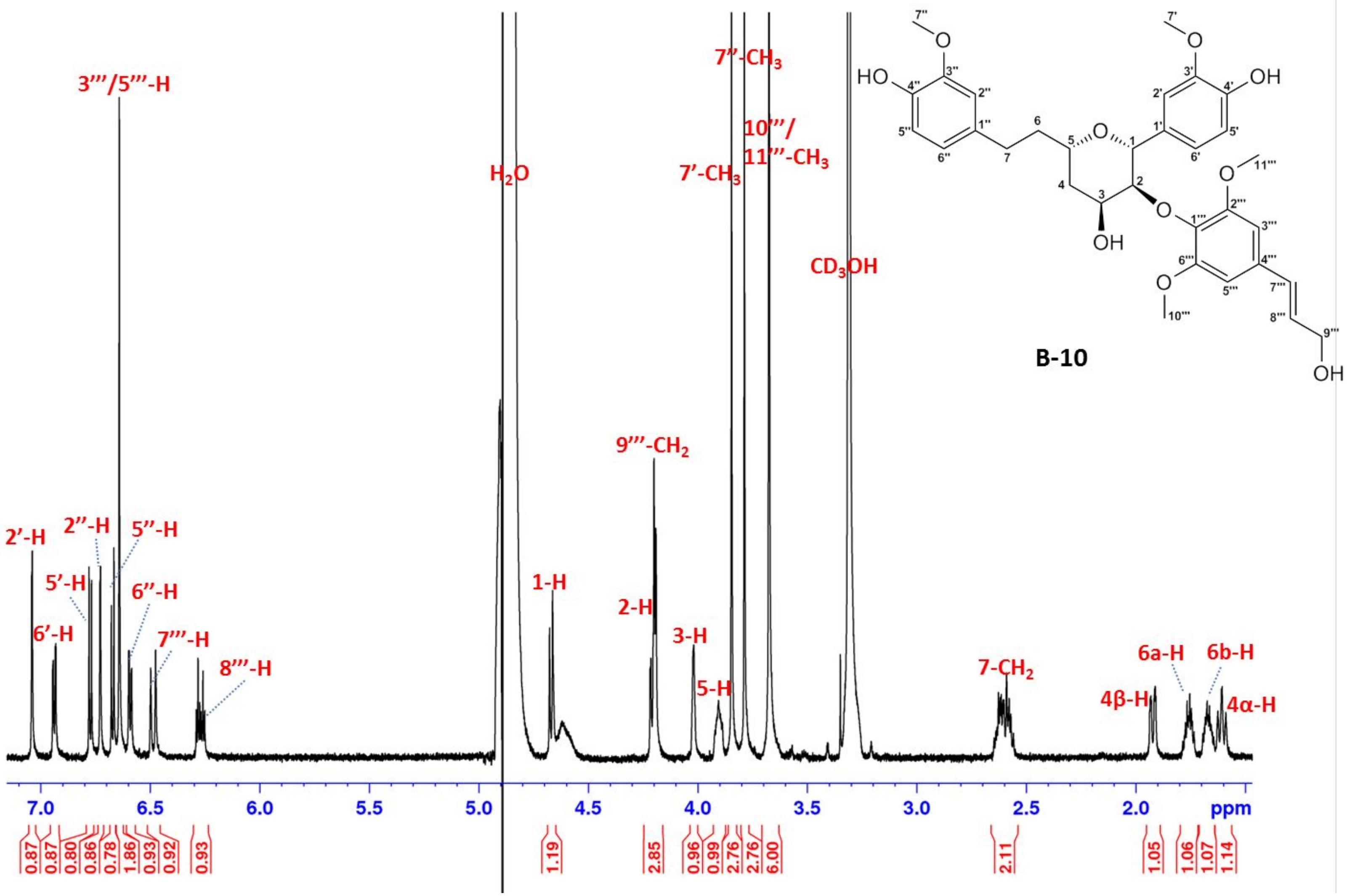

Figure S25. ^{1}H NMR spectrum with water suppression (700 MHz, CD_3OH) of compound **B-10**.

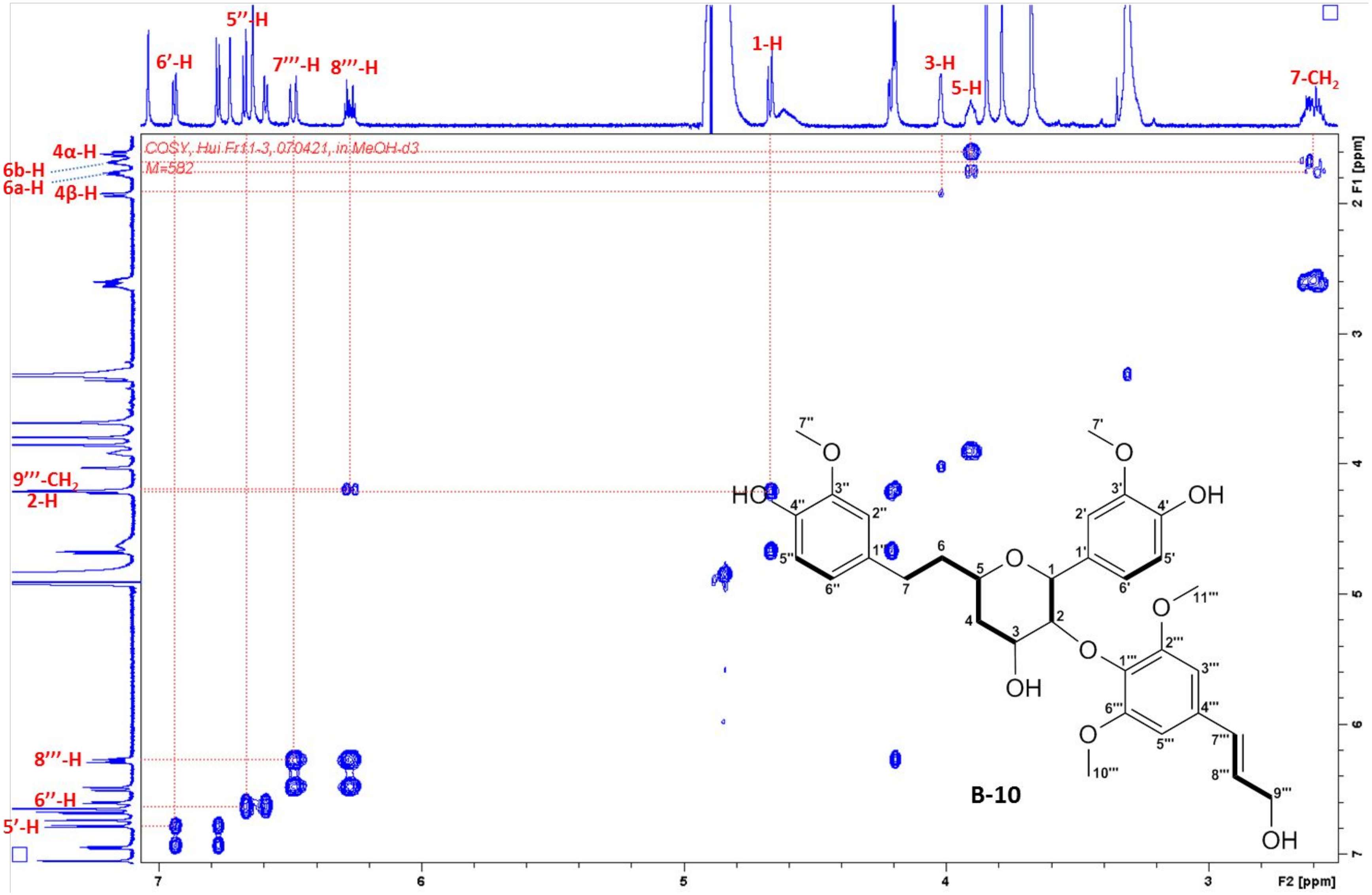

Figure S26. ^{1}H–^{1}H COSY spectrum of compound **B-10** in CD_3OH.

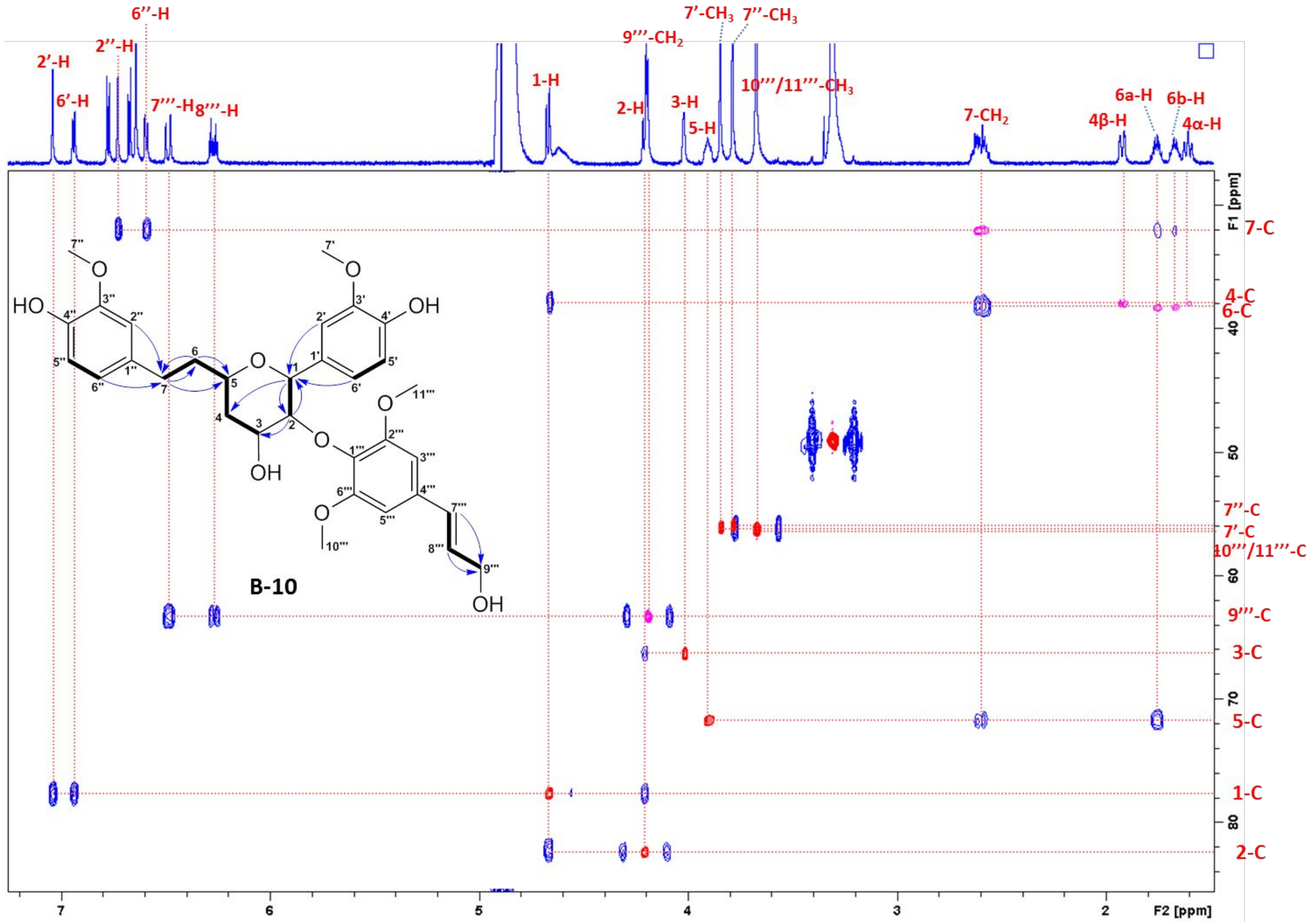

Figure S27. Superimposed HSQC and HMBC spectra of compound **B-10** in CD_3OH (part-1).

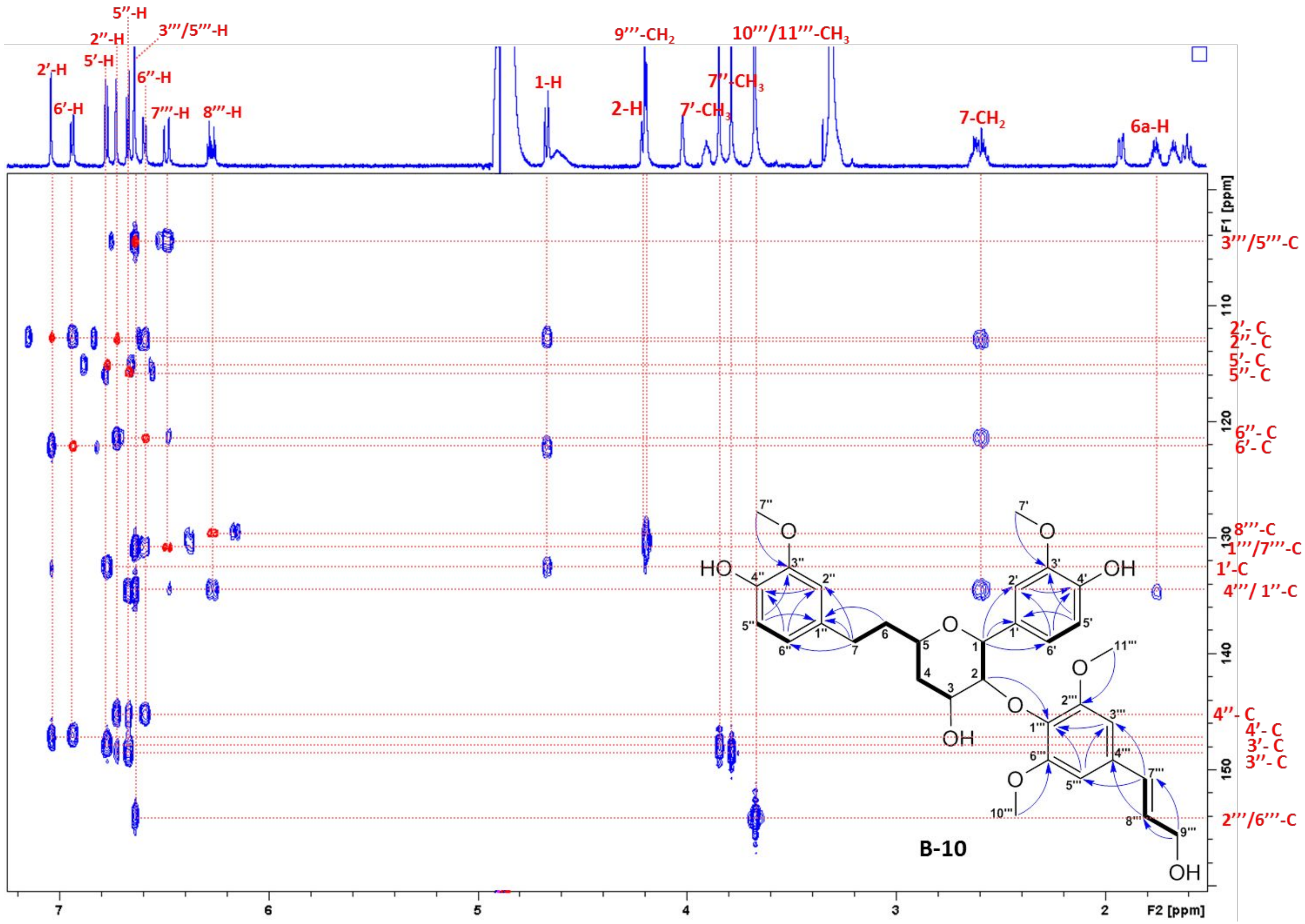

Figure S28. Superimposed HSQC and HMBC spectra of compound **B-10** in CD_3OH (part-2).

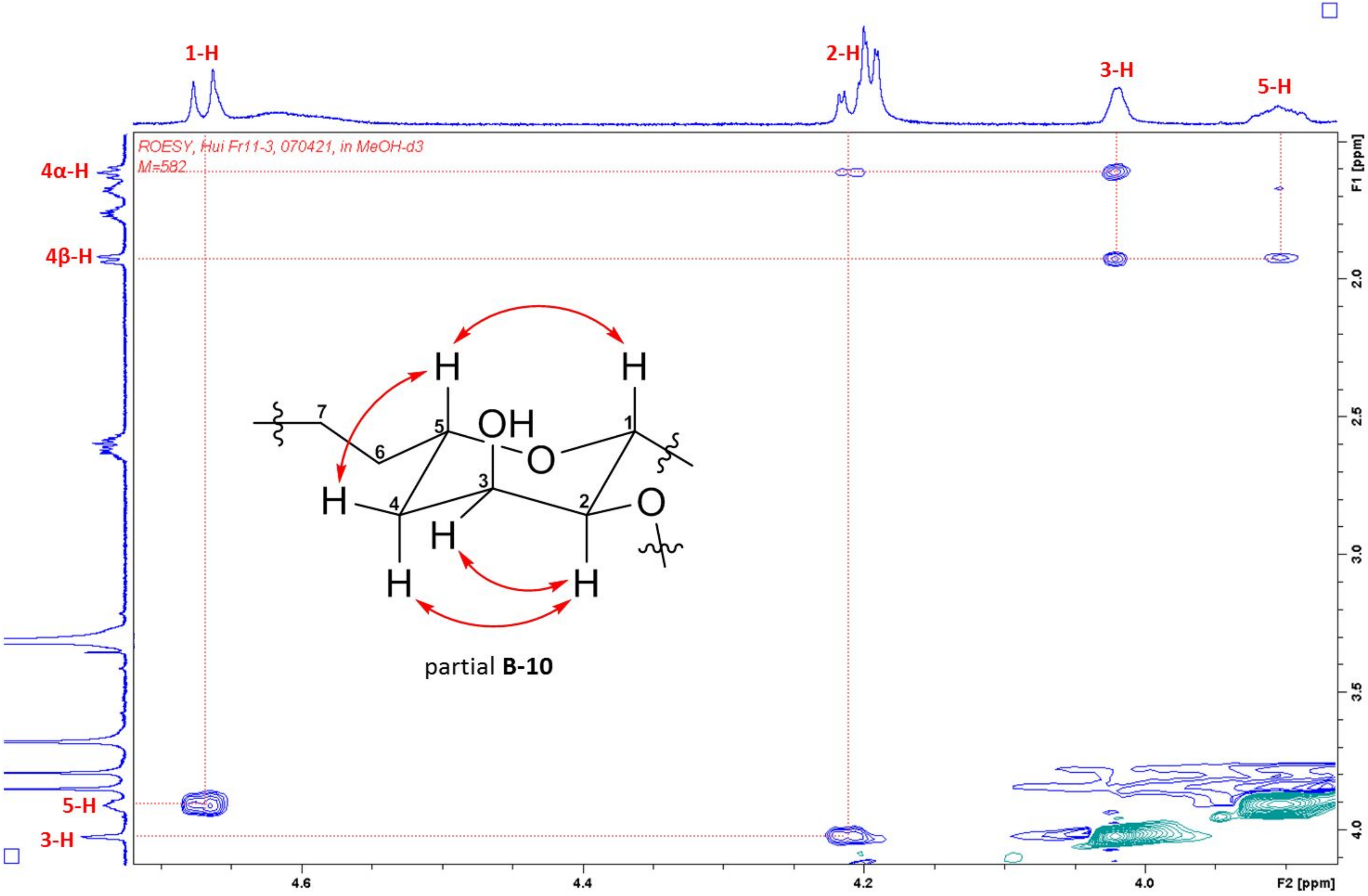

Figure S29. ROESY spectrum of compound **B-10** in CD_3OH.

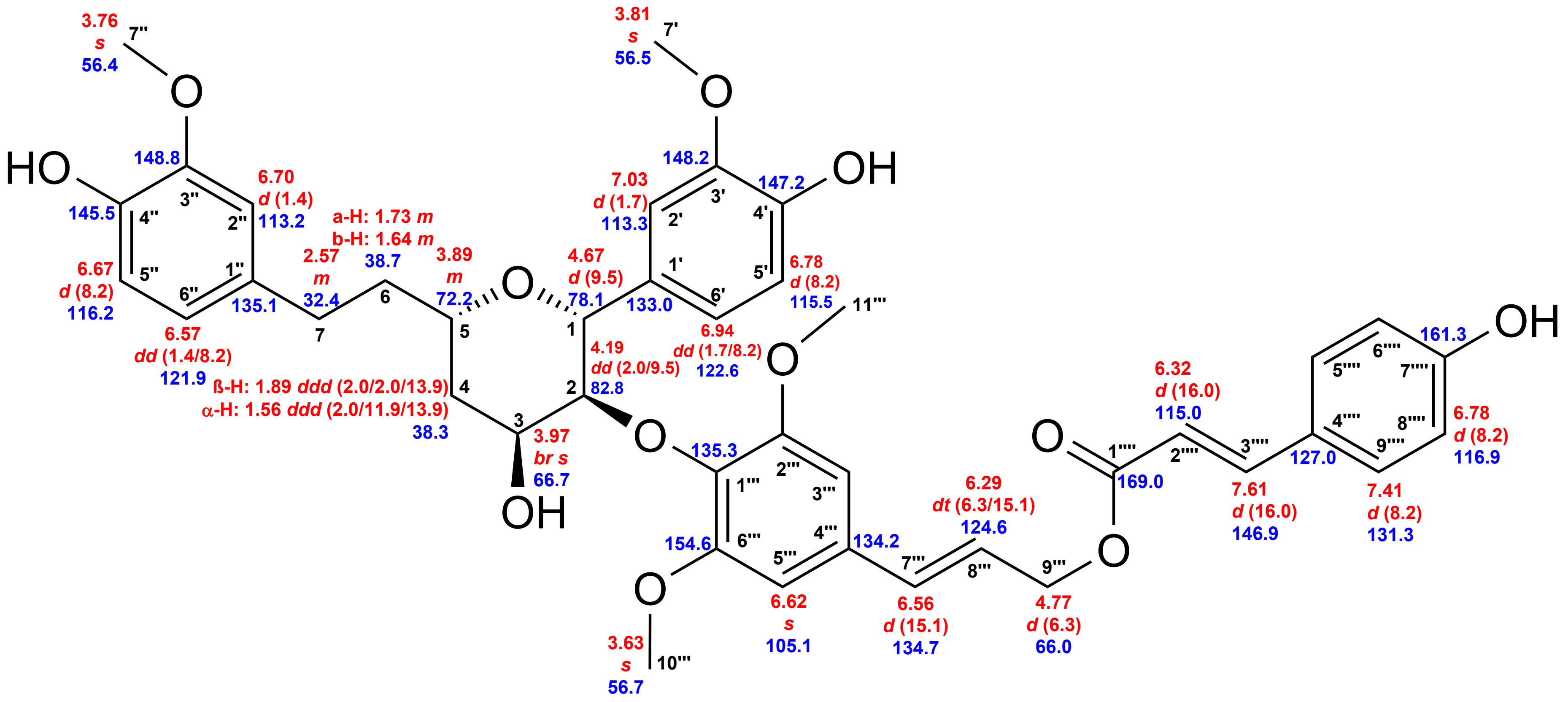

Figure S30. Chemical shifts of compound **B-16**.

Red: [1]H chemical shifts (δ ppm, *mult*., $^3J_{HH}$ in Hz). Blue: [13]C chemical shifts (δ ppm).

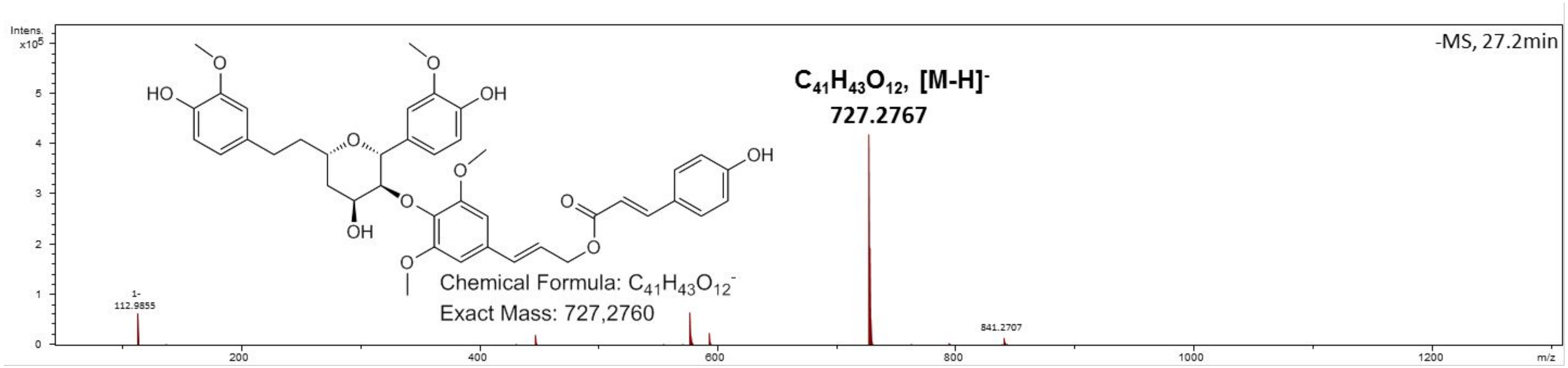

Figure S31. HR-ESI-MS spectrum of compound **B-16**.

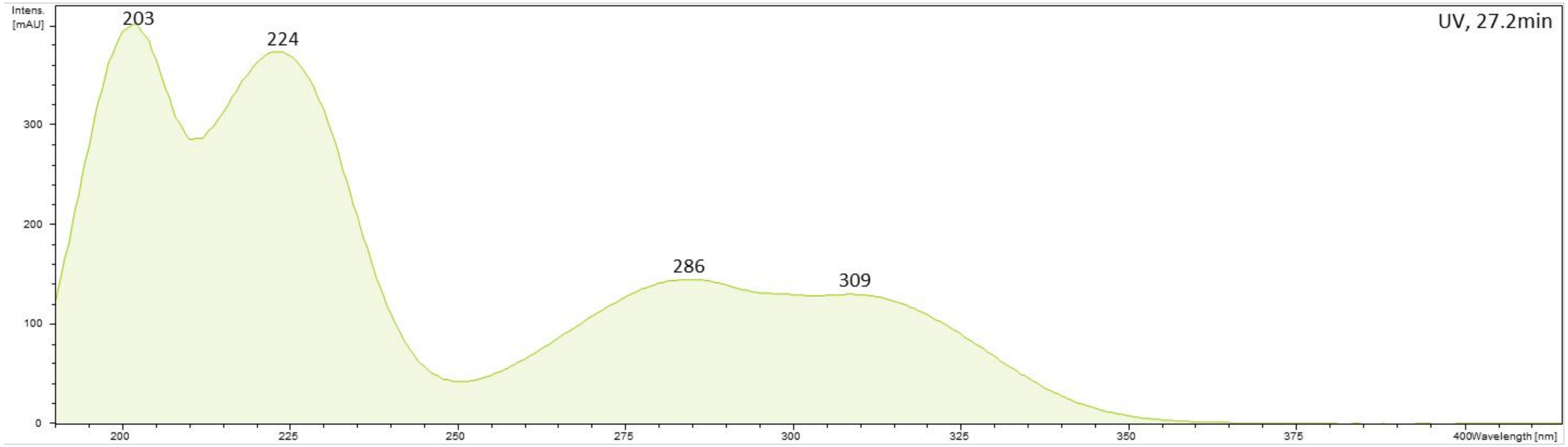

Figure S32. UV/Vis spectrum (MeCN-H_2O) of compound **B-16**.

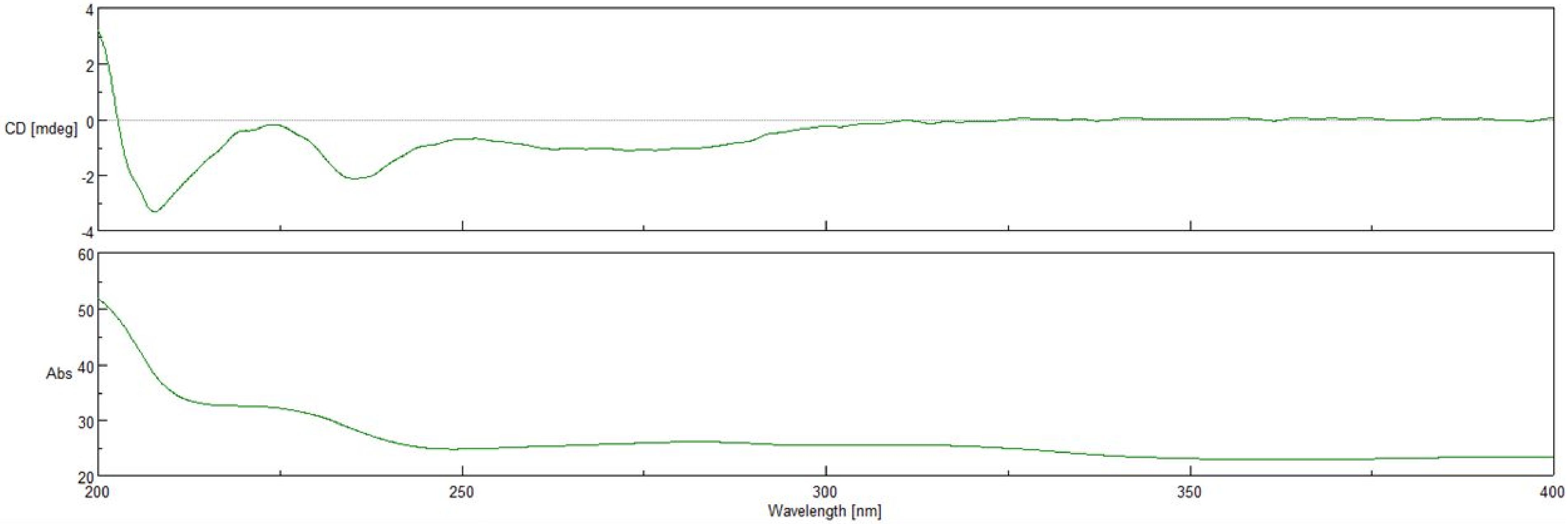

Figure S33. Experimental ECD (upper) and UV (lower) spectra (MeOH) of compound **B-16**.

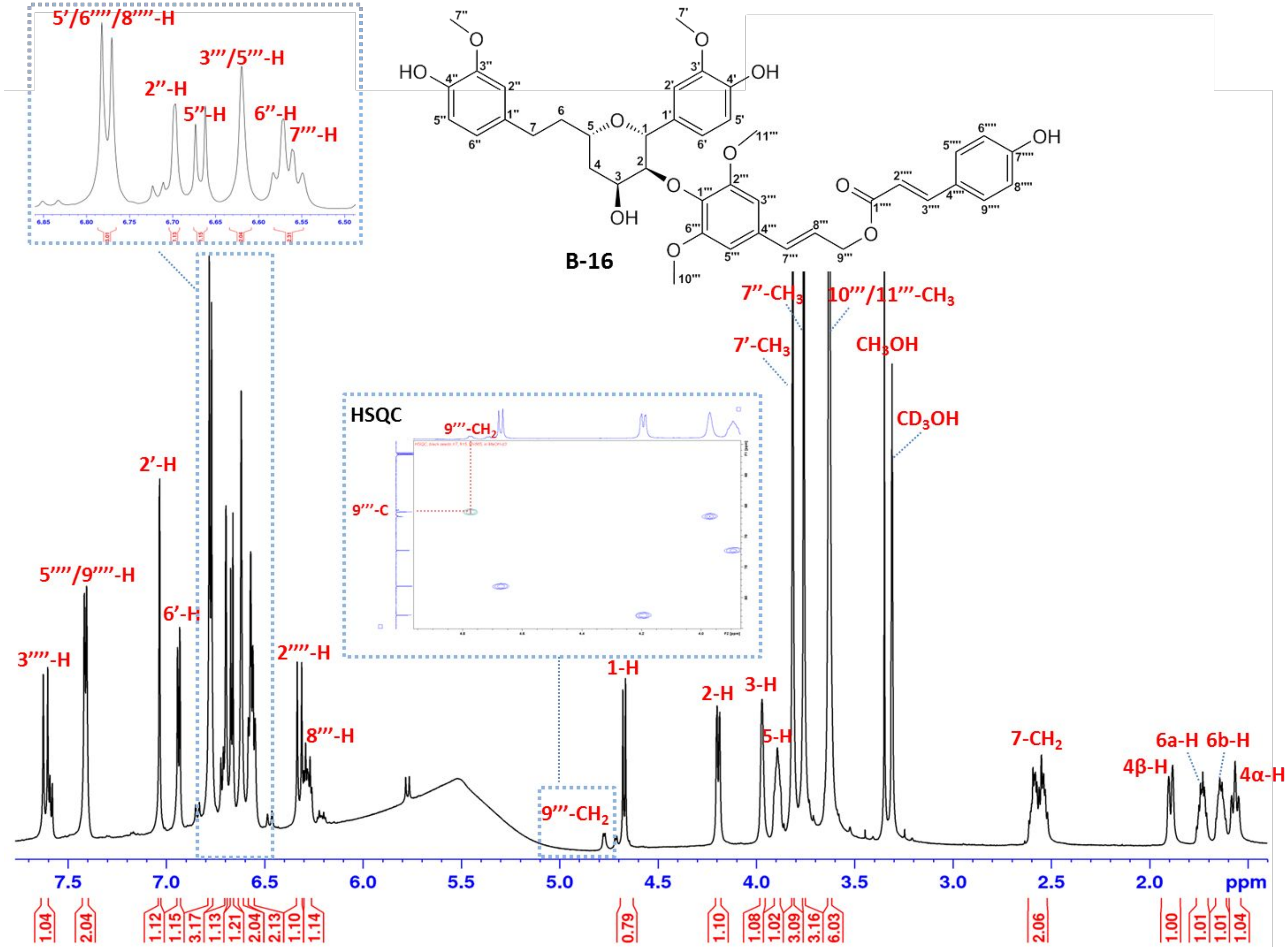

Figure S34. ^{1}H NMR spectrum (700 MHz, CD_3OH) of compound **B-16**.

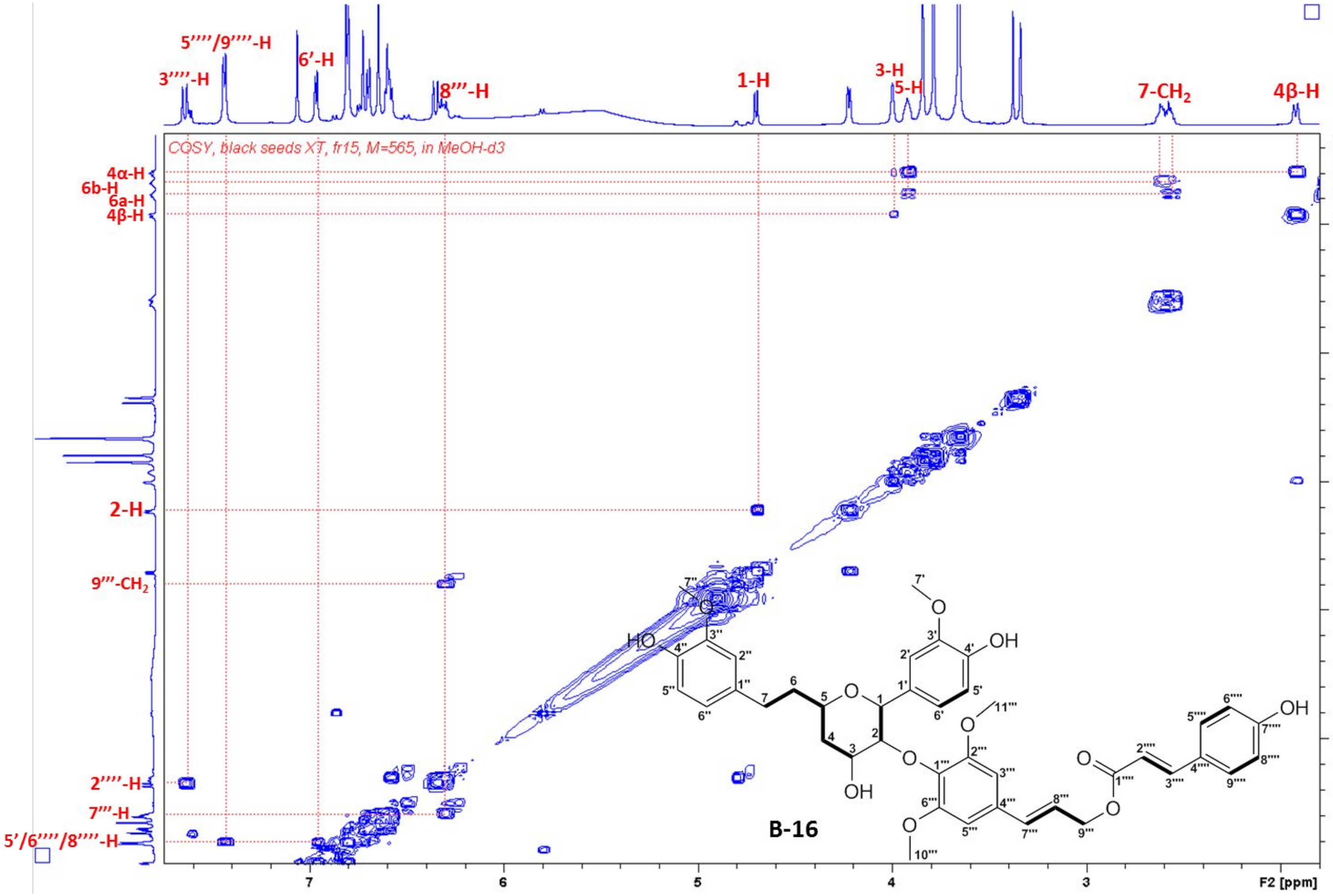

Figure S35. ^{1}H–^{1}H COSY spectrum of compound **B-16** in CD_3OH.

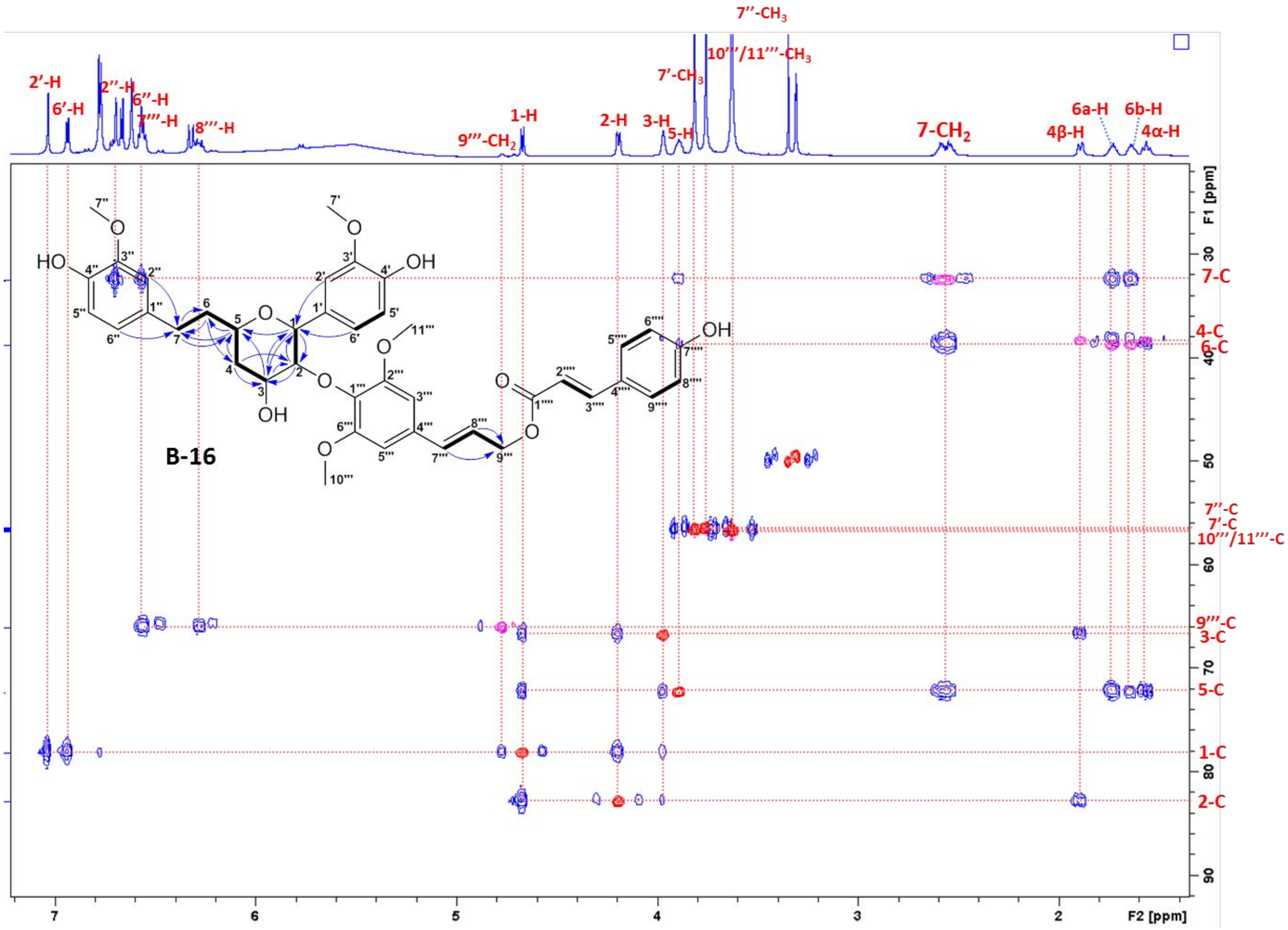

Figure S36. Superimposed HSQC and HMBC spectra of compound **B-16** in CD_3OH (part-1).

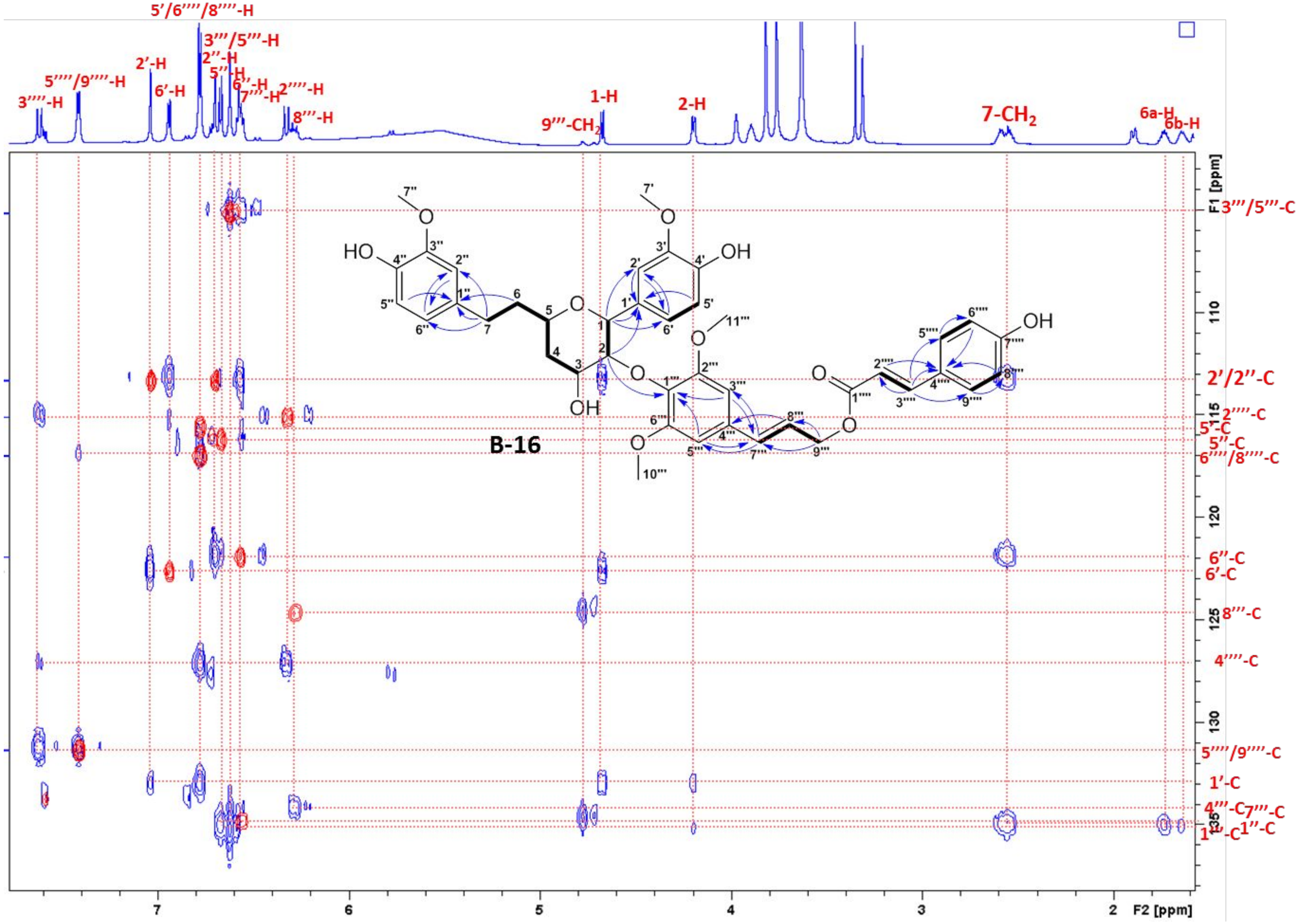

Figure S37. Superimposed HSQC and HMBC spectra of compound **B-16** in CD_3OH (part-2).

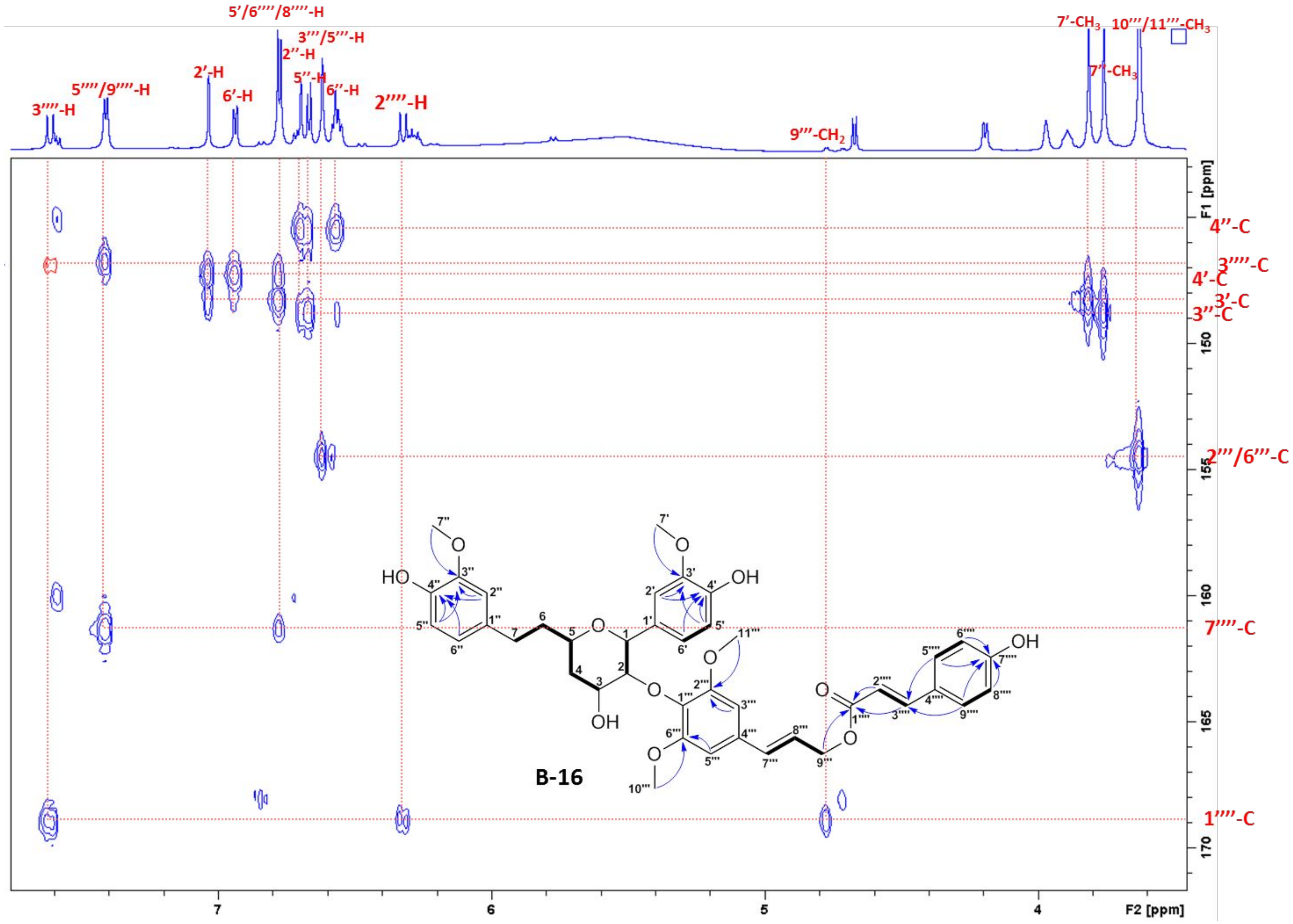

Figure S38. Superimposed HSQC and HMBC spectra of compound **B-16** in CD_3OH (part-3).

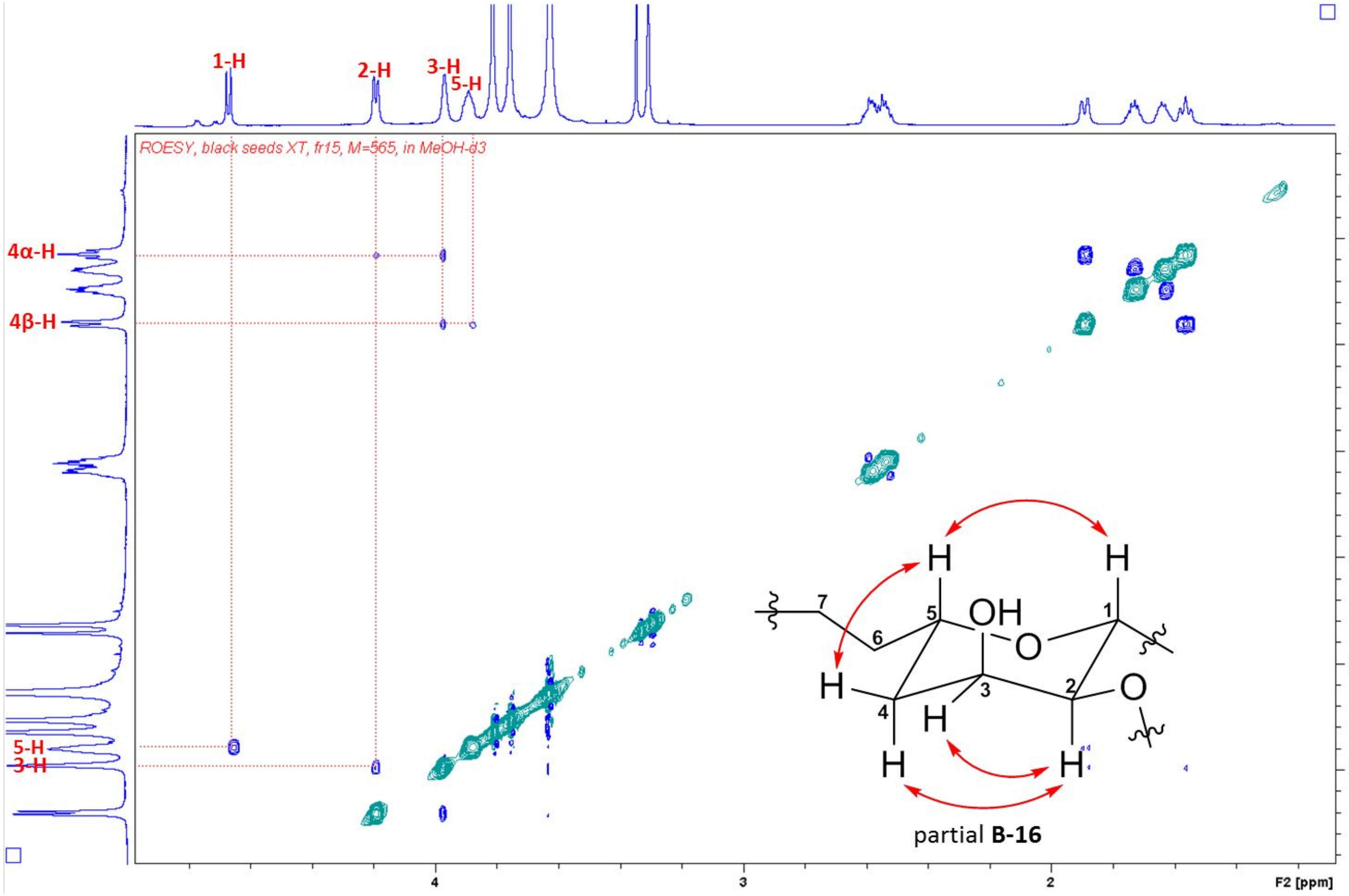

Figure S39. ROESY spectrum of compound **B-16** in CD_3OH.

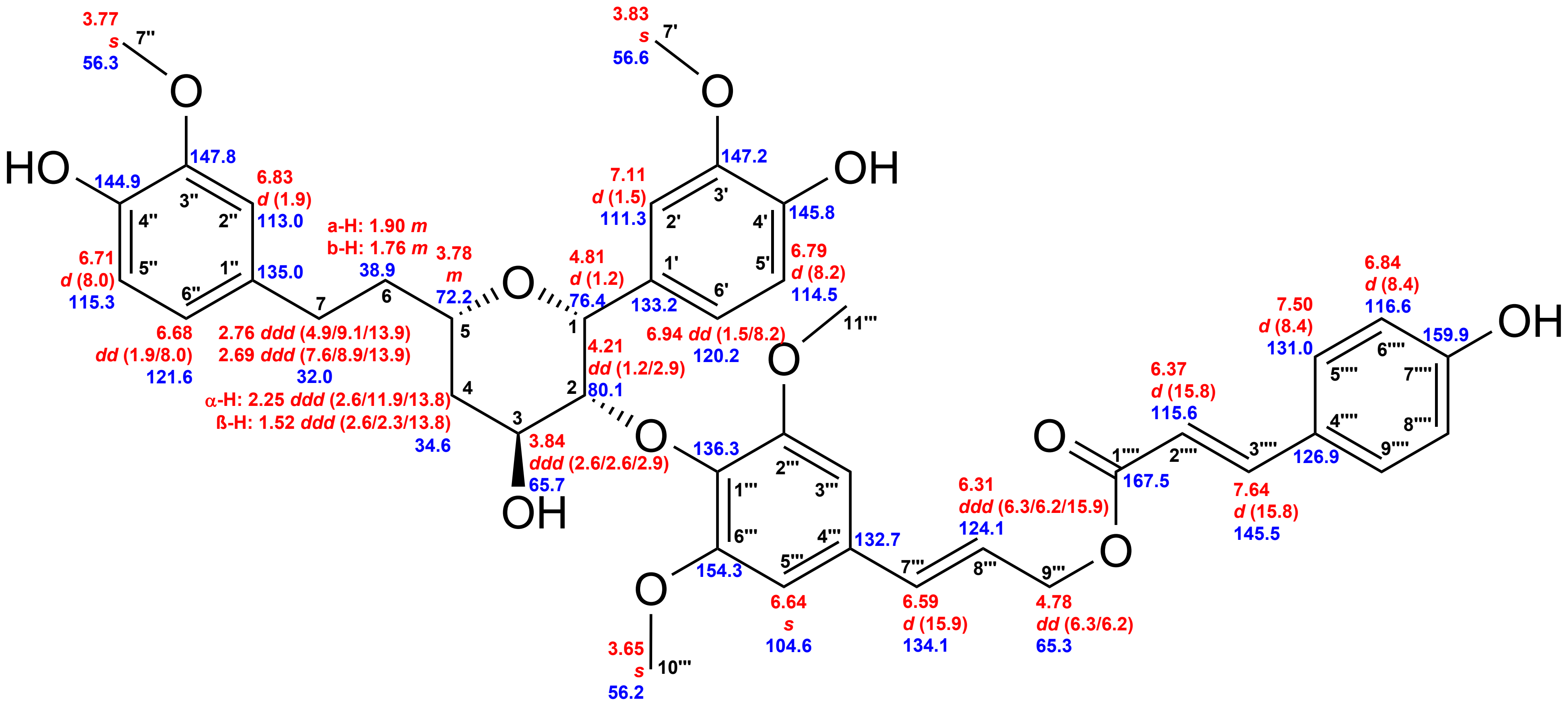

Figure S40. Chemical shifts of compound **B-14**.

Red: ^{1}H chemical shifts (δ ppm, *mult*., $^{3}J_{HH}$ in Hz). Blue: ^{13}C chemical shifts (δ ppm).

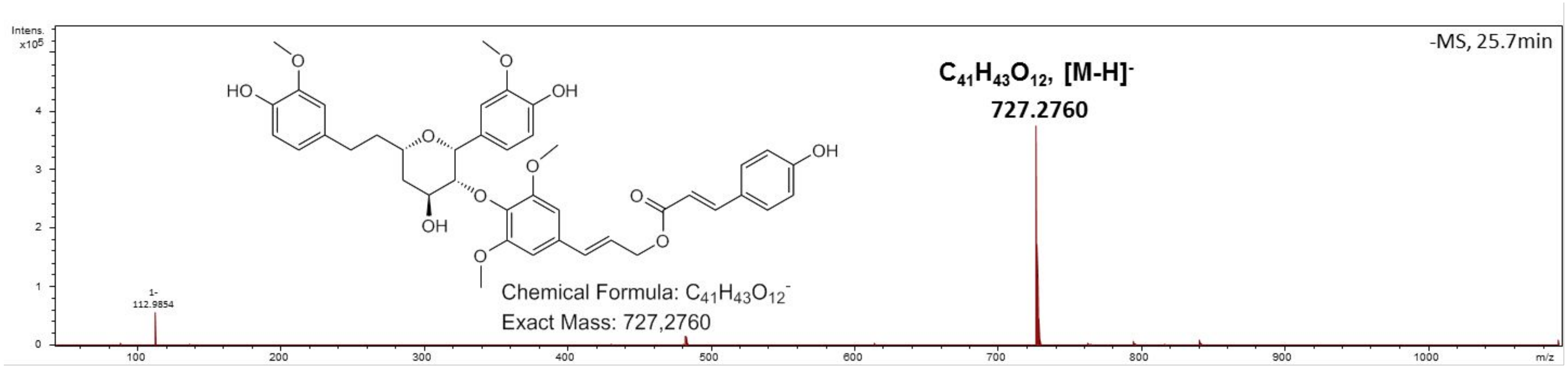

Figure S41. HR-ESI-MS spectrum of compound **B-14**.

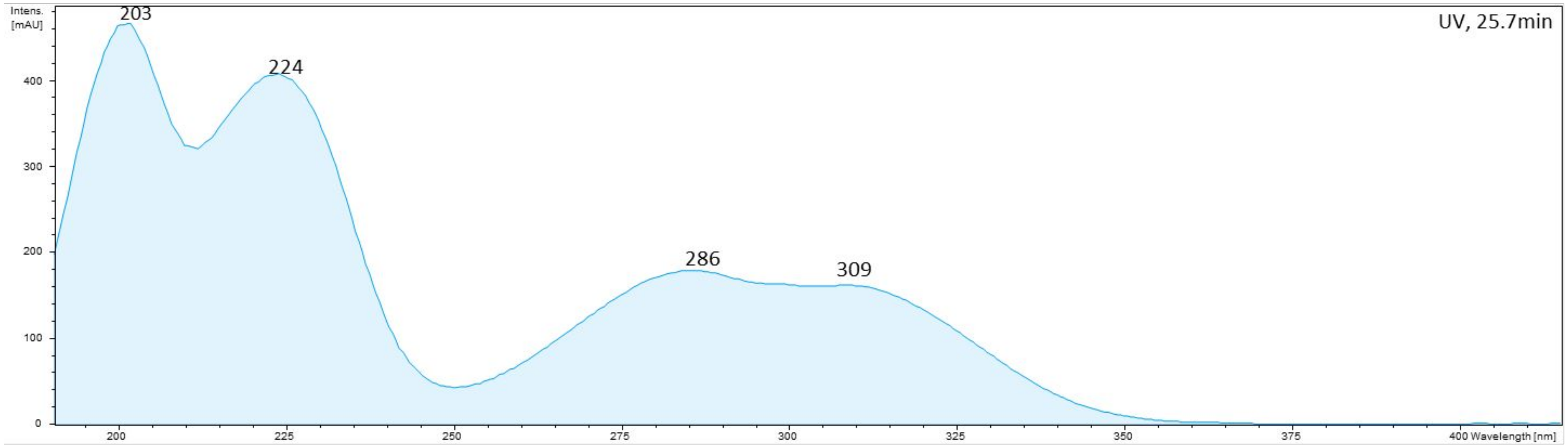

Figure S42. UV/Vis spectrum (MeCN-H_2O) of compound **B-14**.

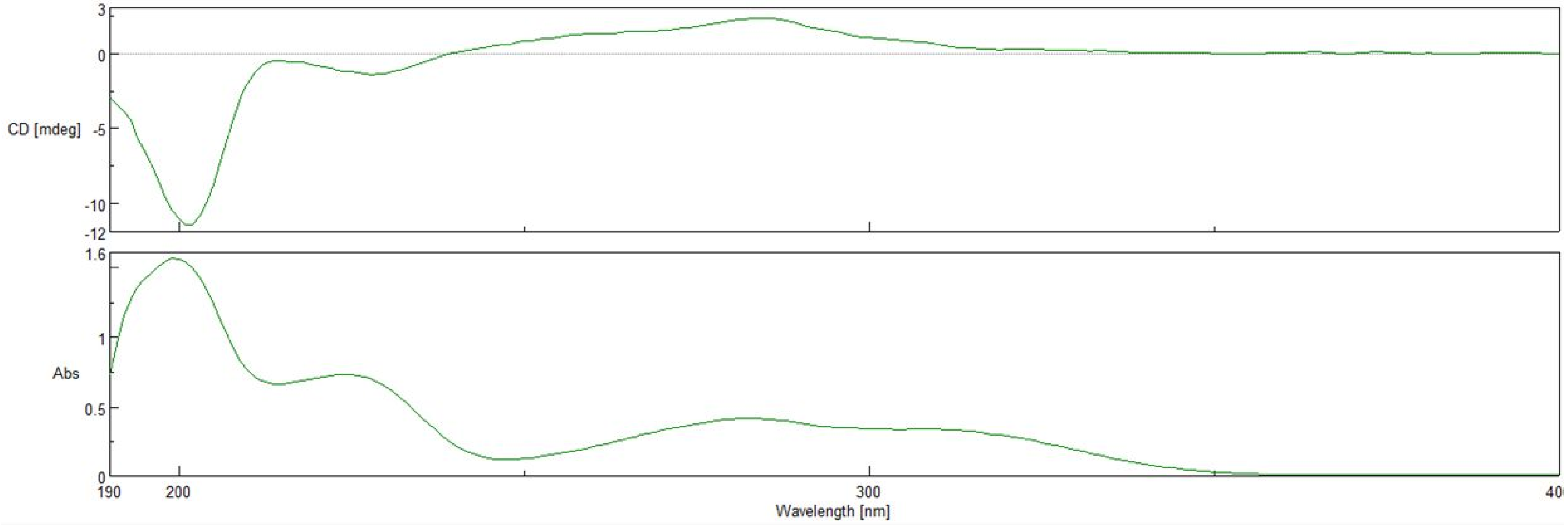

Figure S43. Experimental ECD (upper) and UV (lower) spectra (MeOH) of compound **B-14**.

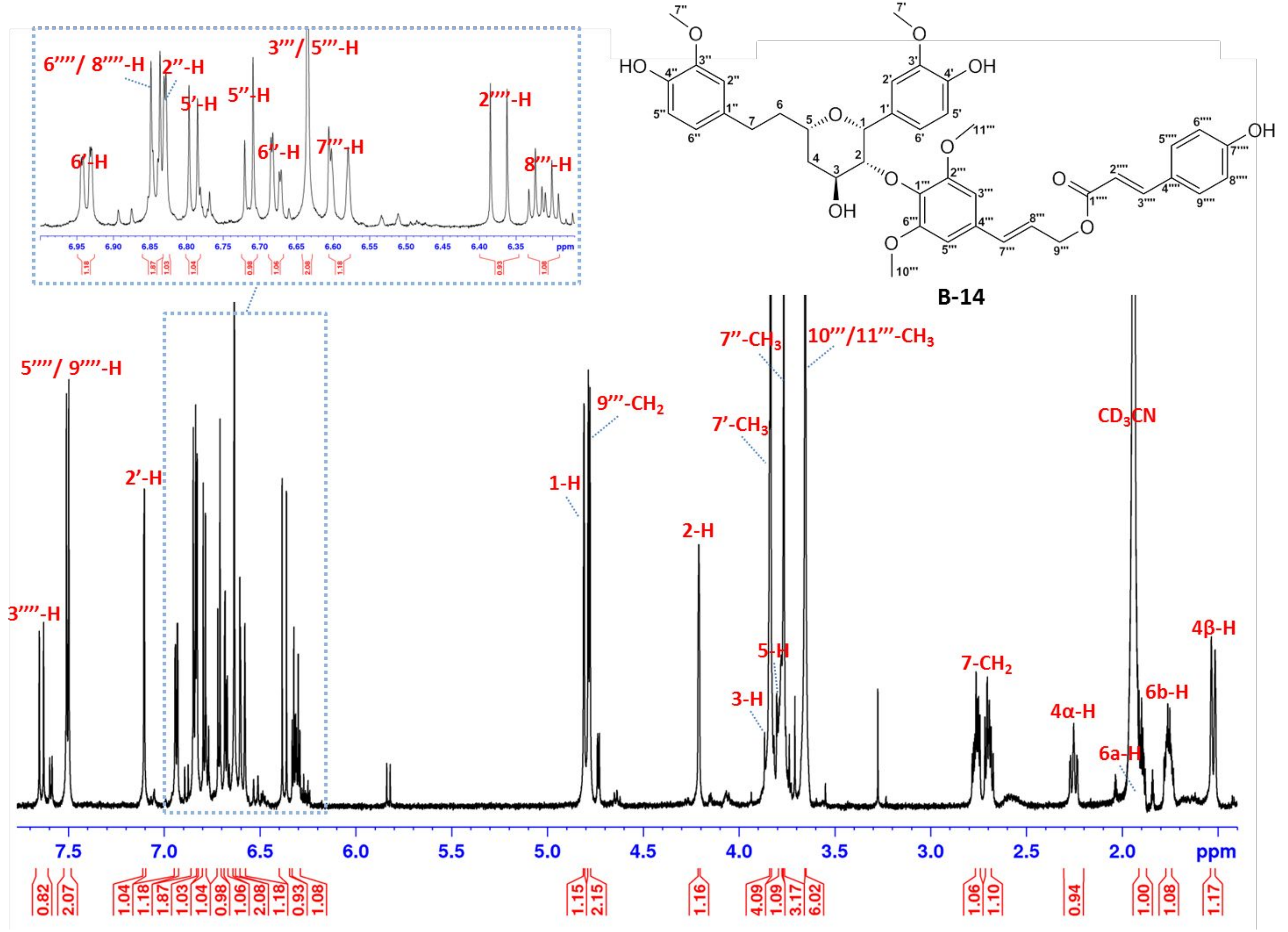

Figure S44. ^{1}H NMR spectrum (700 MHz, CD_3CN) of compound **B-14**.

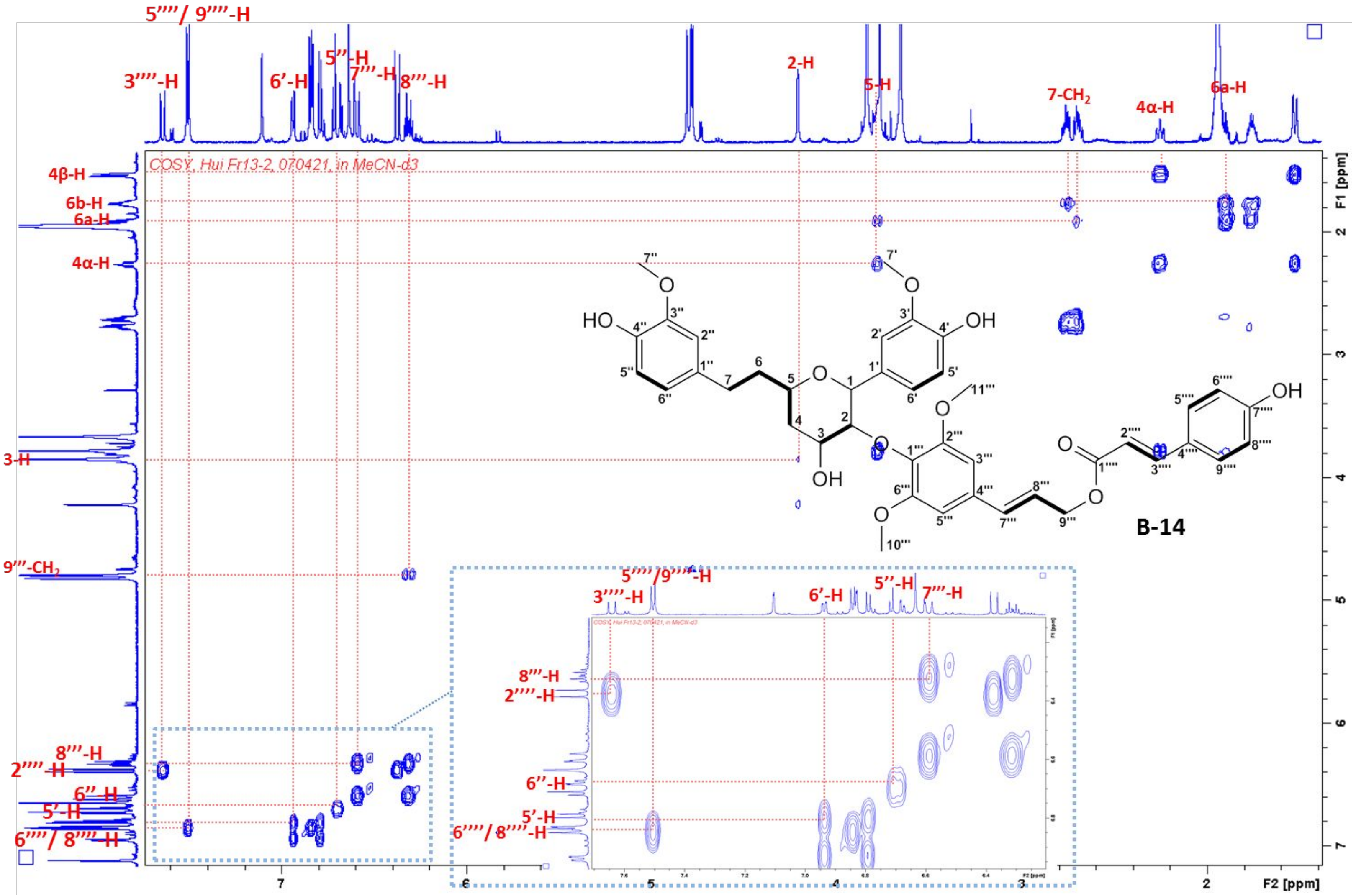

Figure S45. ^{1}H–^{1}H COSY spectrum of compound **B-14** in CD_3CN.

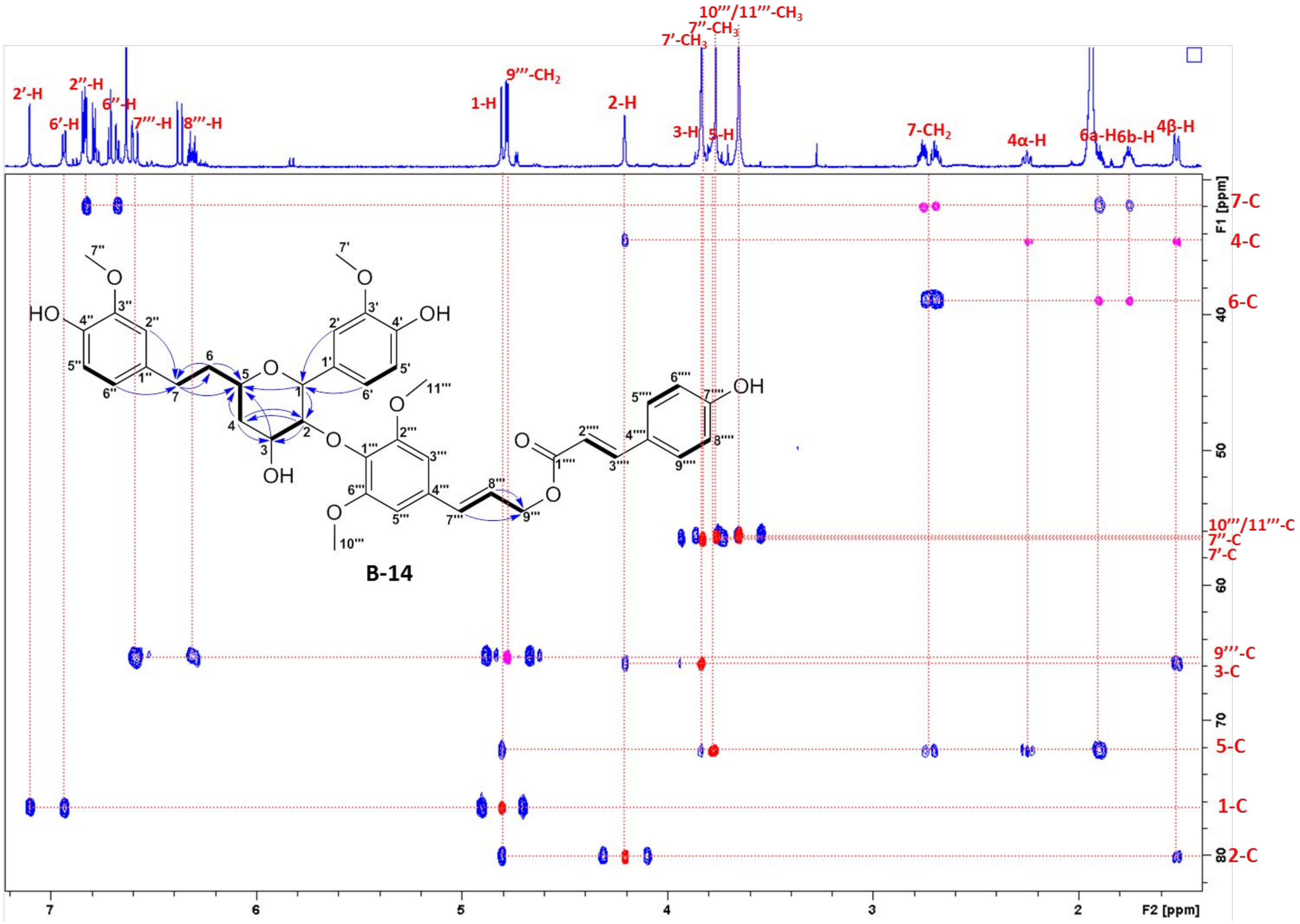

Figure S46. Superimposed HSQC and HMBC spectra of compound **B-14** in CD_3CN (part-1).

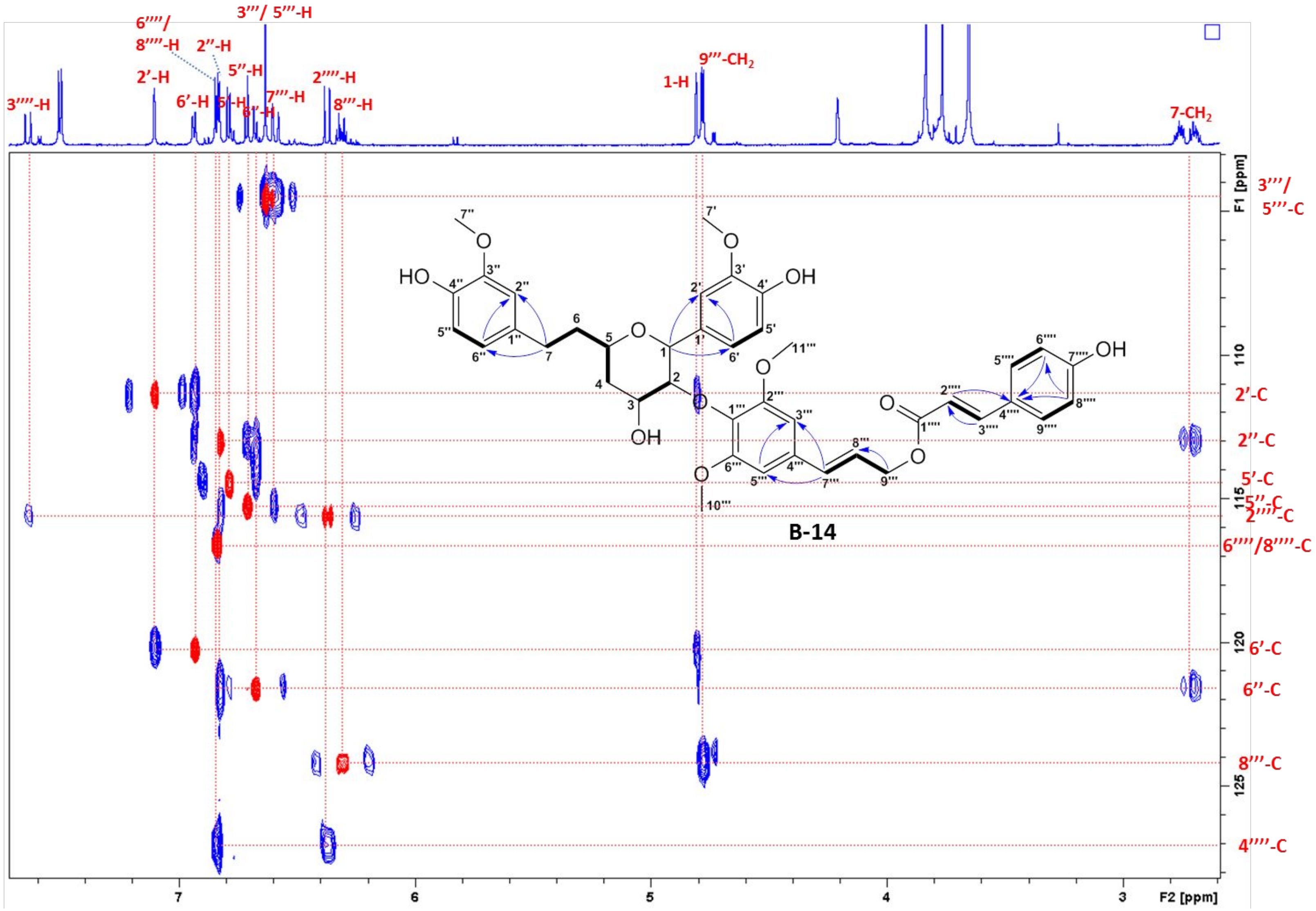

Figure S47. Superimposed HSQC and HMBC spectra of compound **B-14** in CD_3CN (part-2).

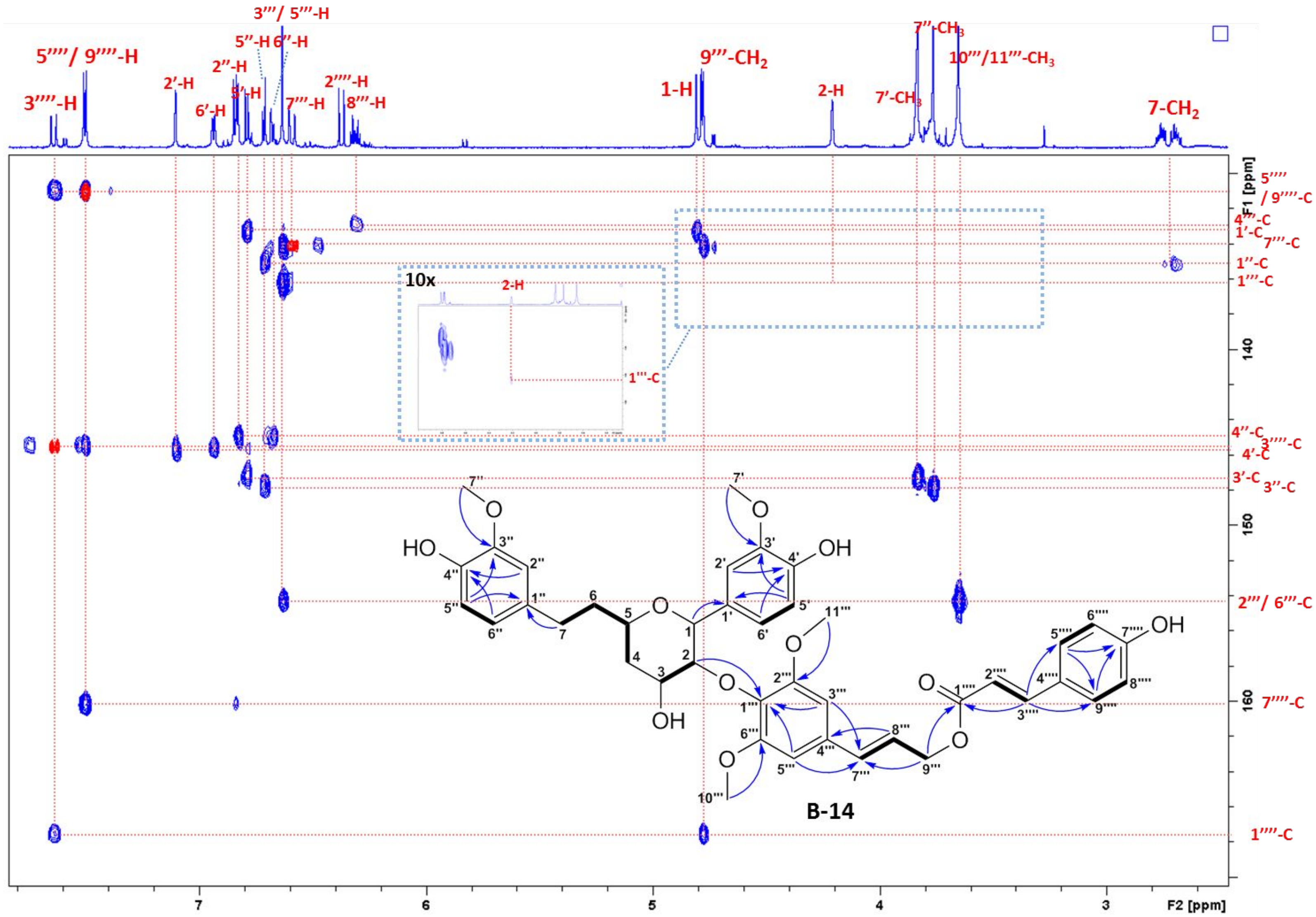

Figure S48. Superimposed HSQC and HMBC spectra of compound **B-14** in CD_3CN (part-3).

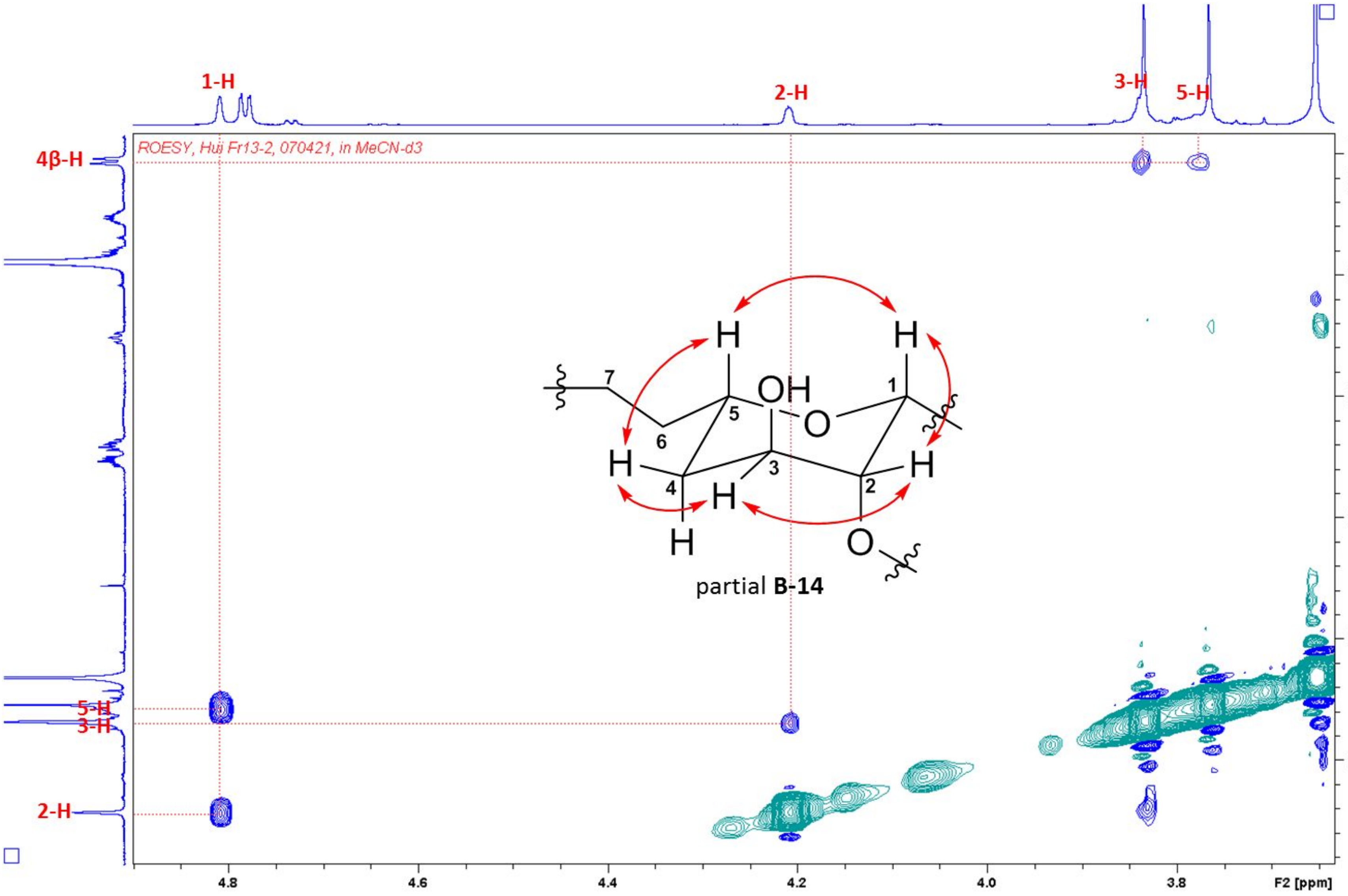

Figure S49. ROESY spectrum of compound **B-14** in CD_3CN.

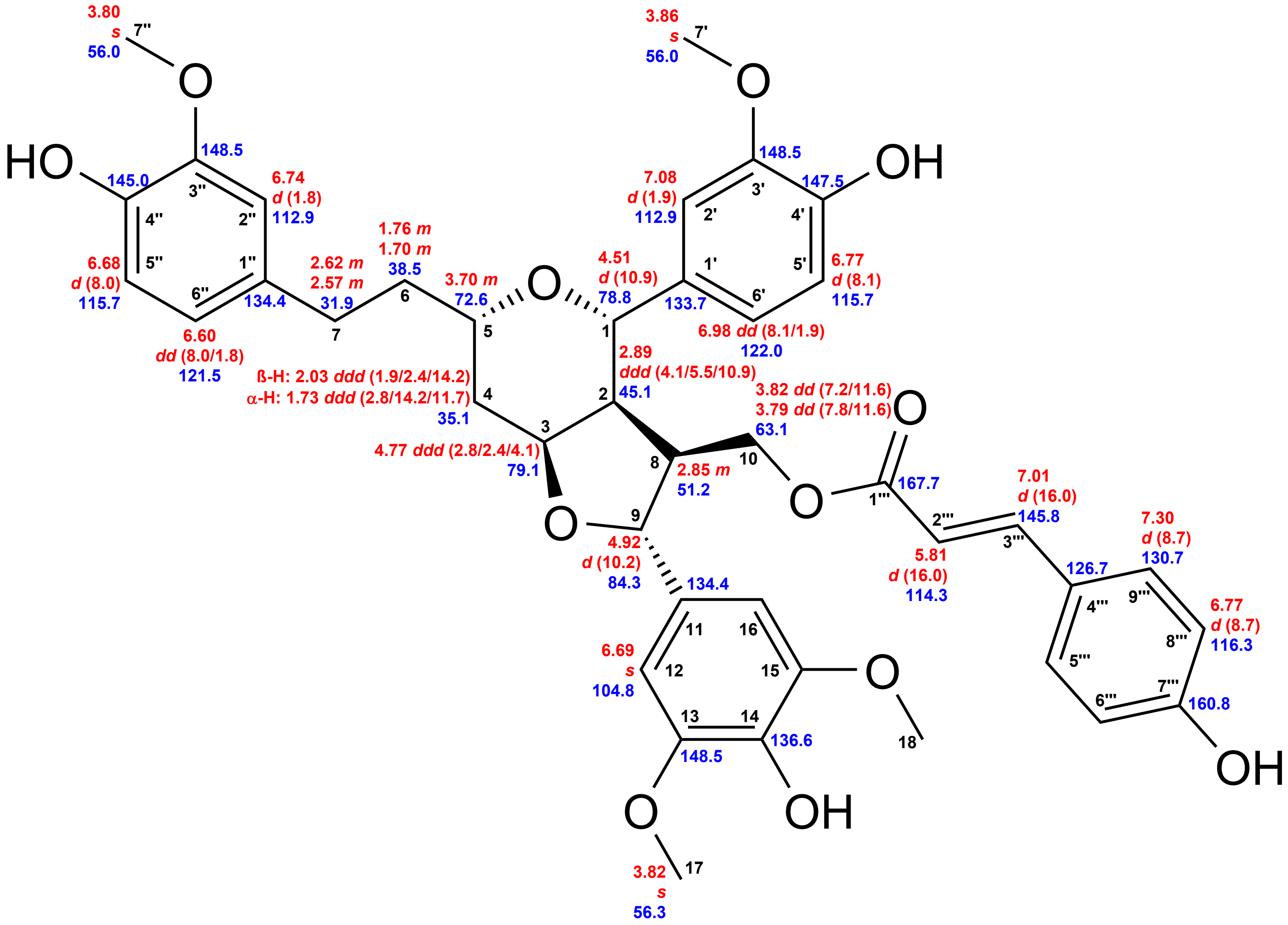

Figure S50. Chemical shifts of compound **B-12**. Red: ^{1}H chemical shifts (δ ppm, *mult.*, $^{3}J_{HH}$ in Hz). Blue: ^{13}C chemical shifts (δ ppm).

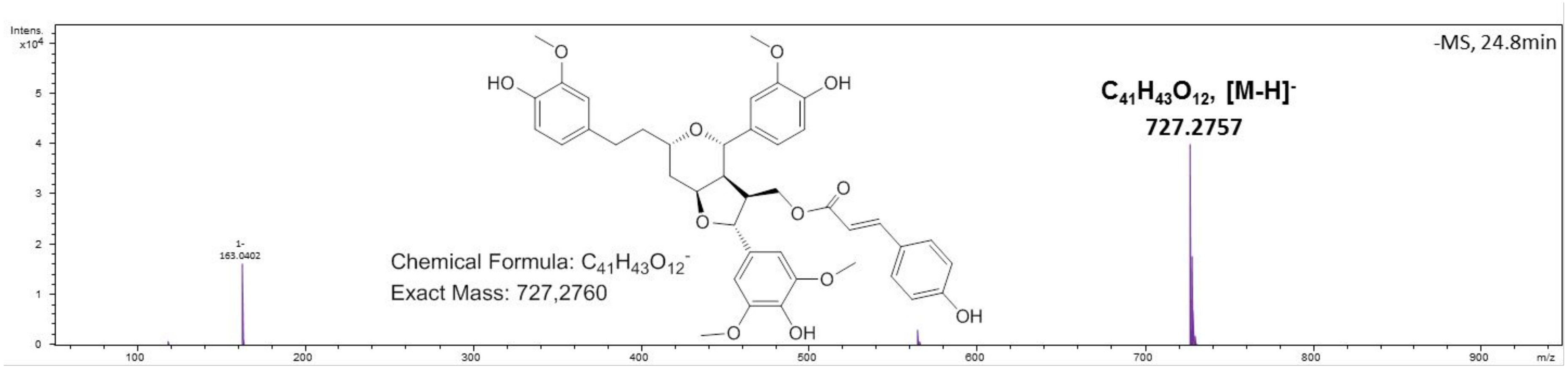

Figure S51. HR-ESI-MS spectrum of compound **B-12**.

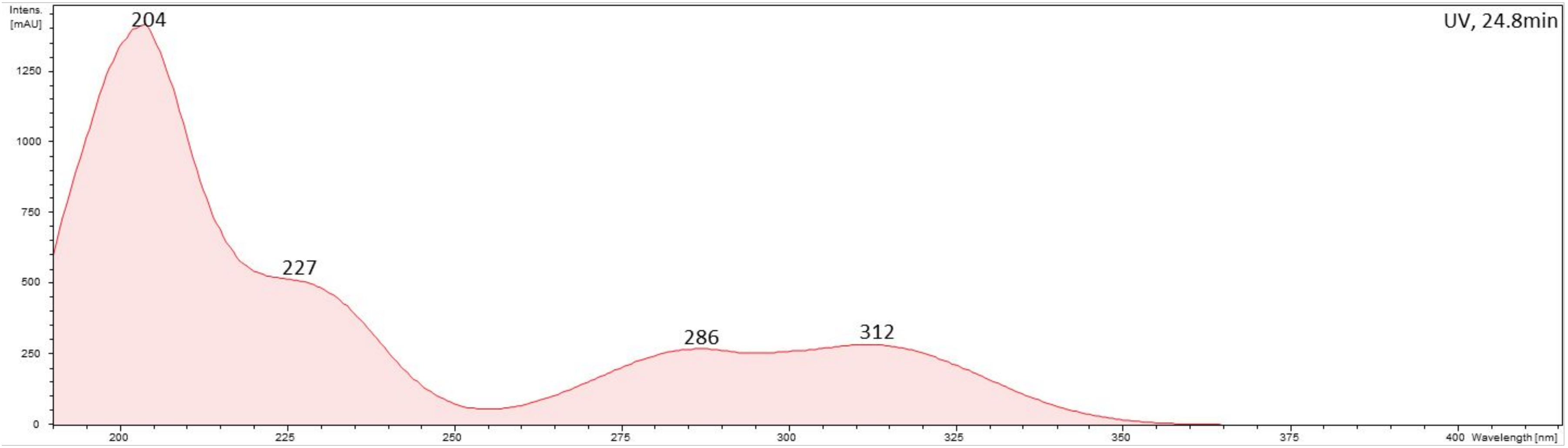

Figure S52. UV/Vis spectrum (MeCN-H_2O) of compound **B-12**.

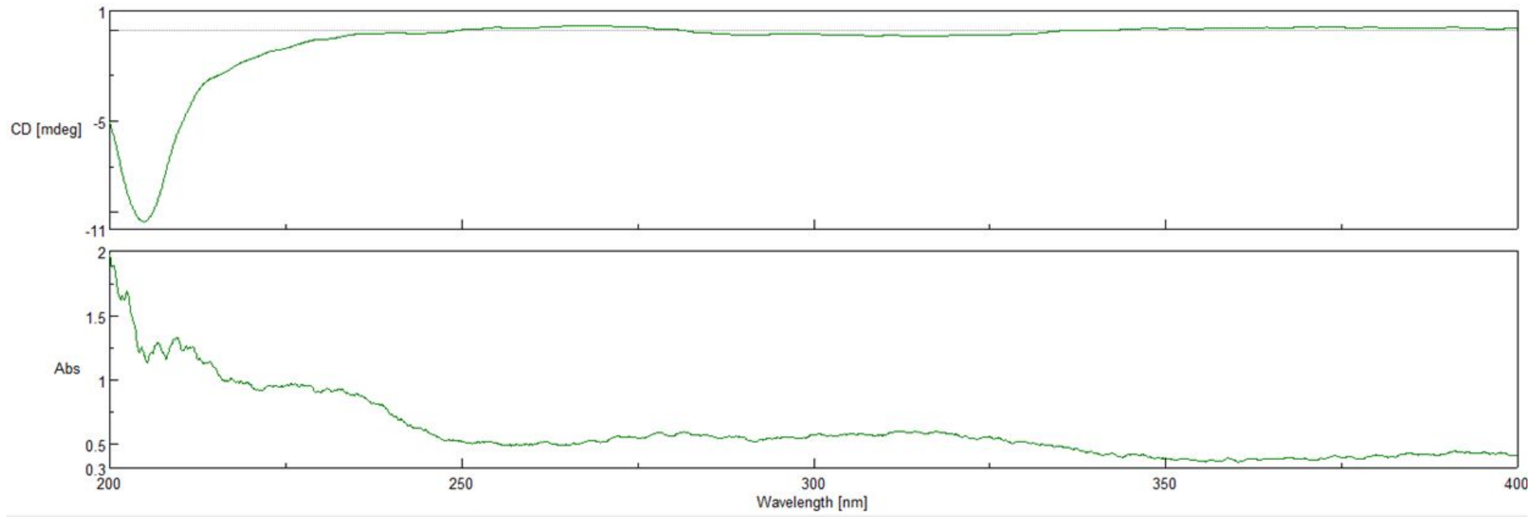

Figure S53. Experimental ECD (upper) and UV (lower) spectra (MeOH) of compound **B-12**.

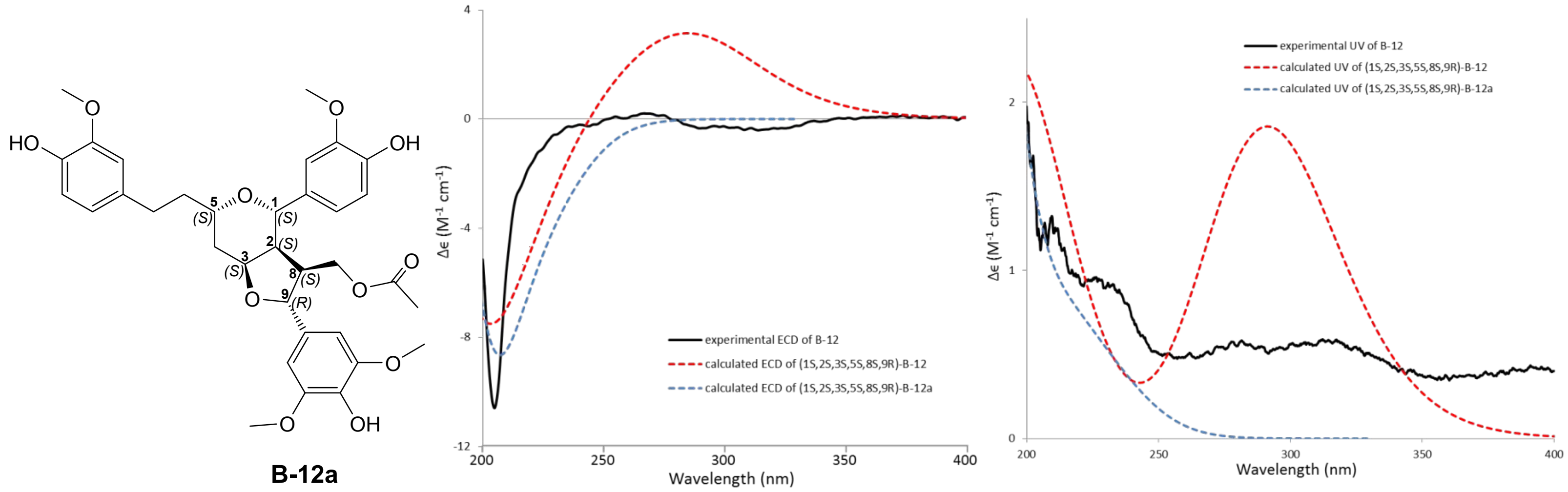

Figure S54. Comparison of experimental and calculated ECD (left) and UV (right) spectra of compounds **B-12** and **B-12a**.

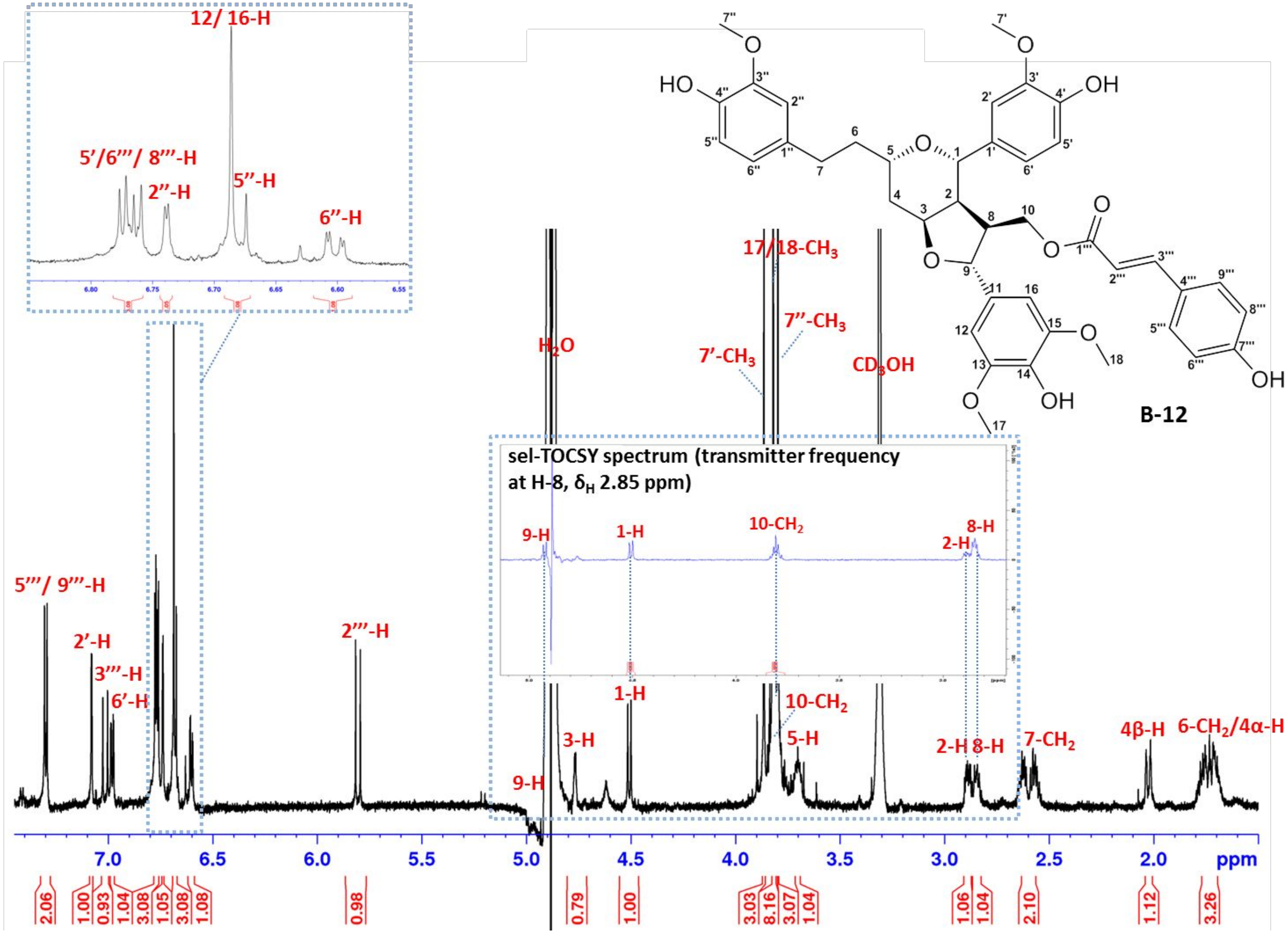

Figure S55. ^{1}H NMR spectrum with water suppression (700 MHz, CD_3OH) of compound **B-12**.

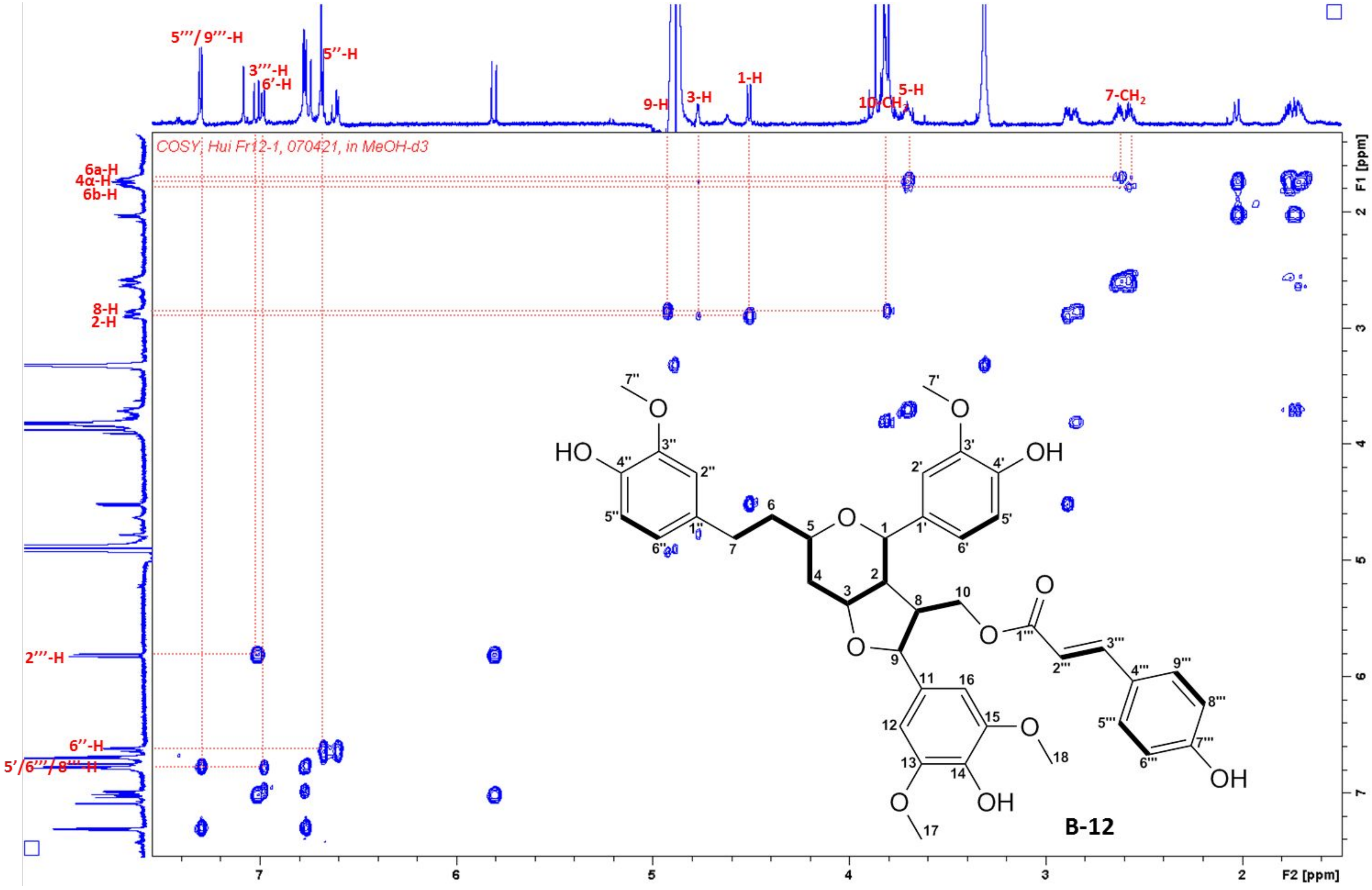

Figure S56. ^1H–^1H COSY spectrum of compound **B-12** in CD_3OH.

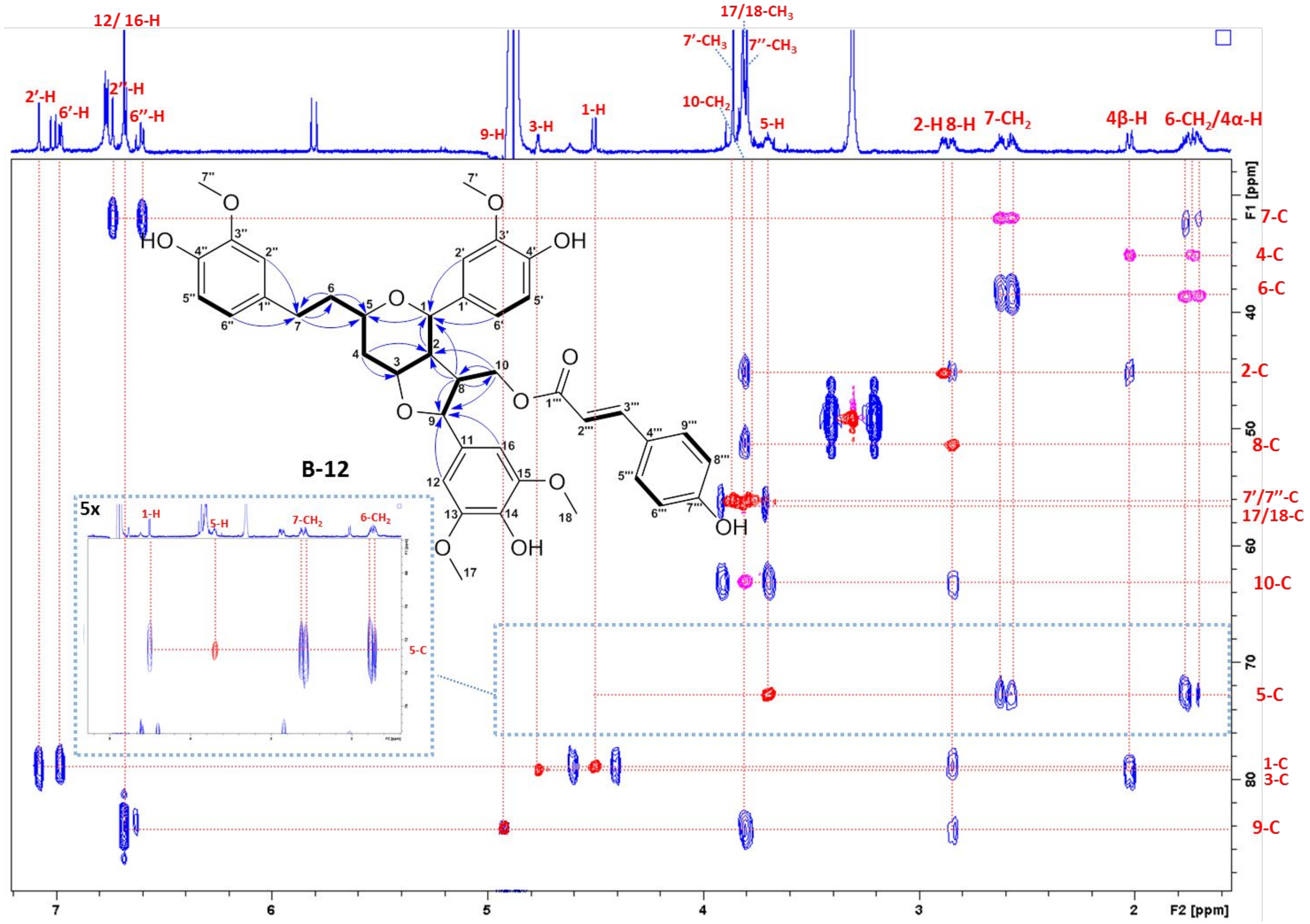

Figure S57. Superimposed HSQC and HMBC spectra of compound **B-12** in CD_3OH (part-1).

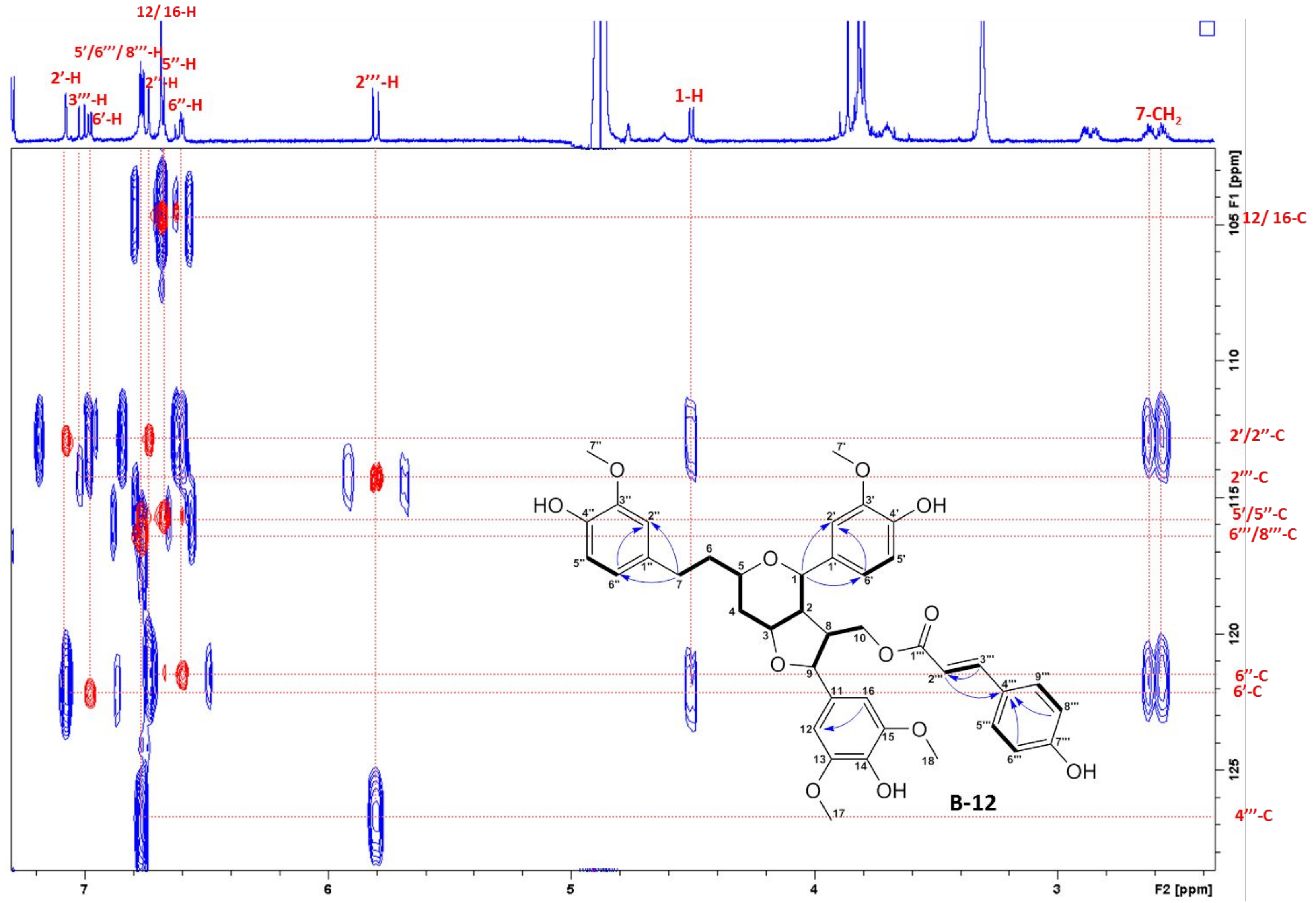

Figure S58. Superimposed HSQC and HMBC spectra of compound **B-12** in CD_3OH (part-2).

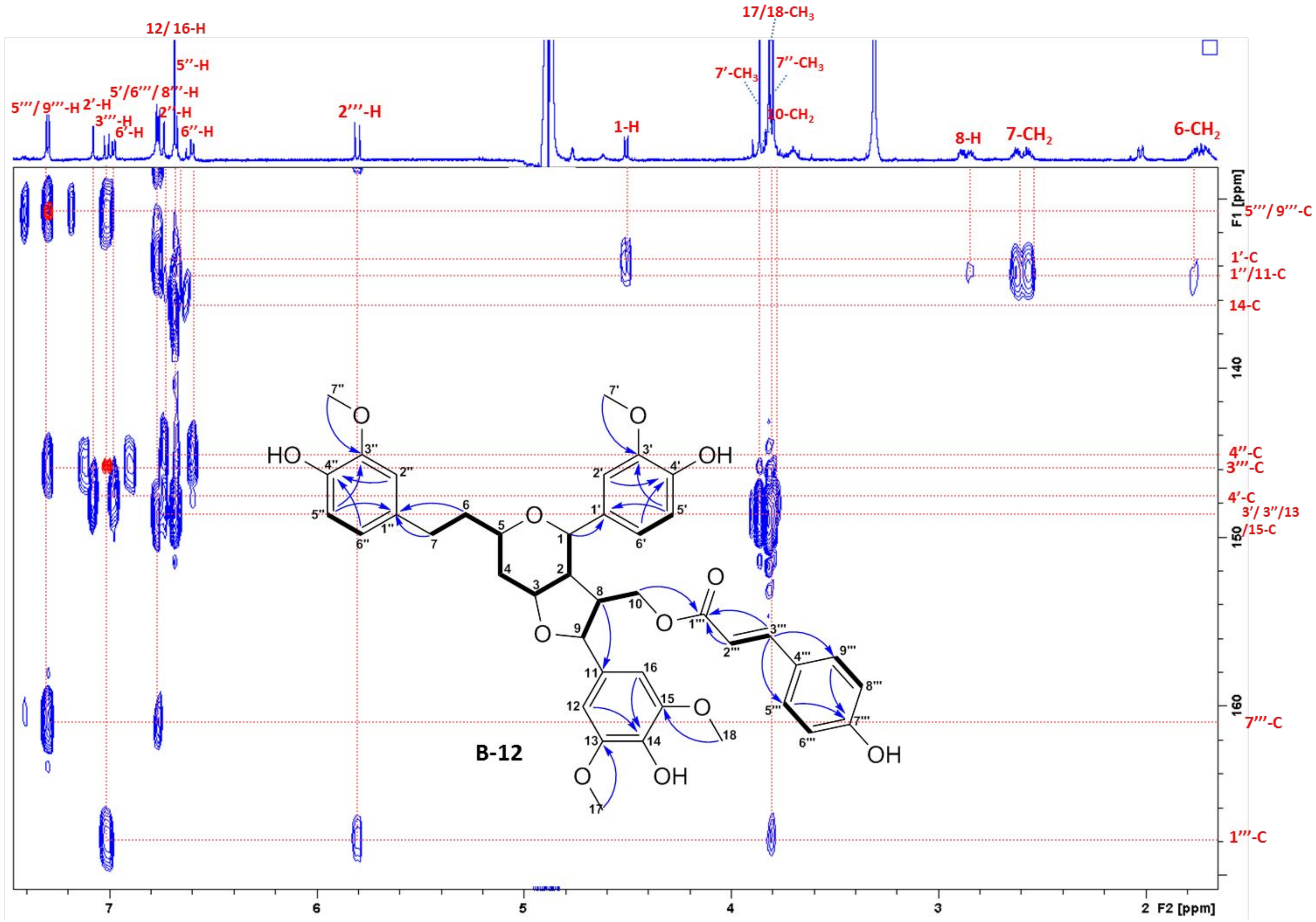

Figure S59. Superimposed HSQC and HMBC spectra of compound **B-12** in CD_3OH (part-3).

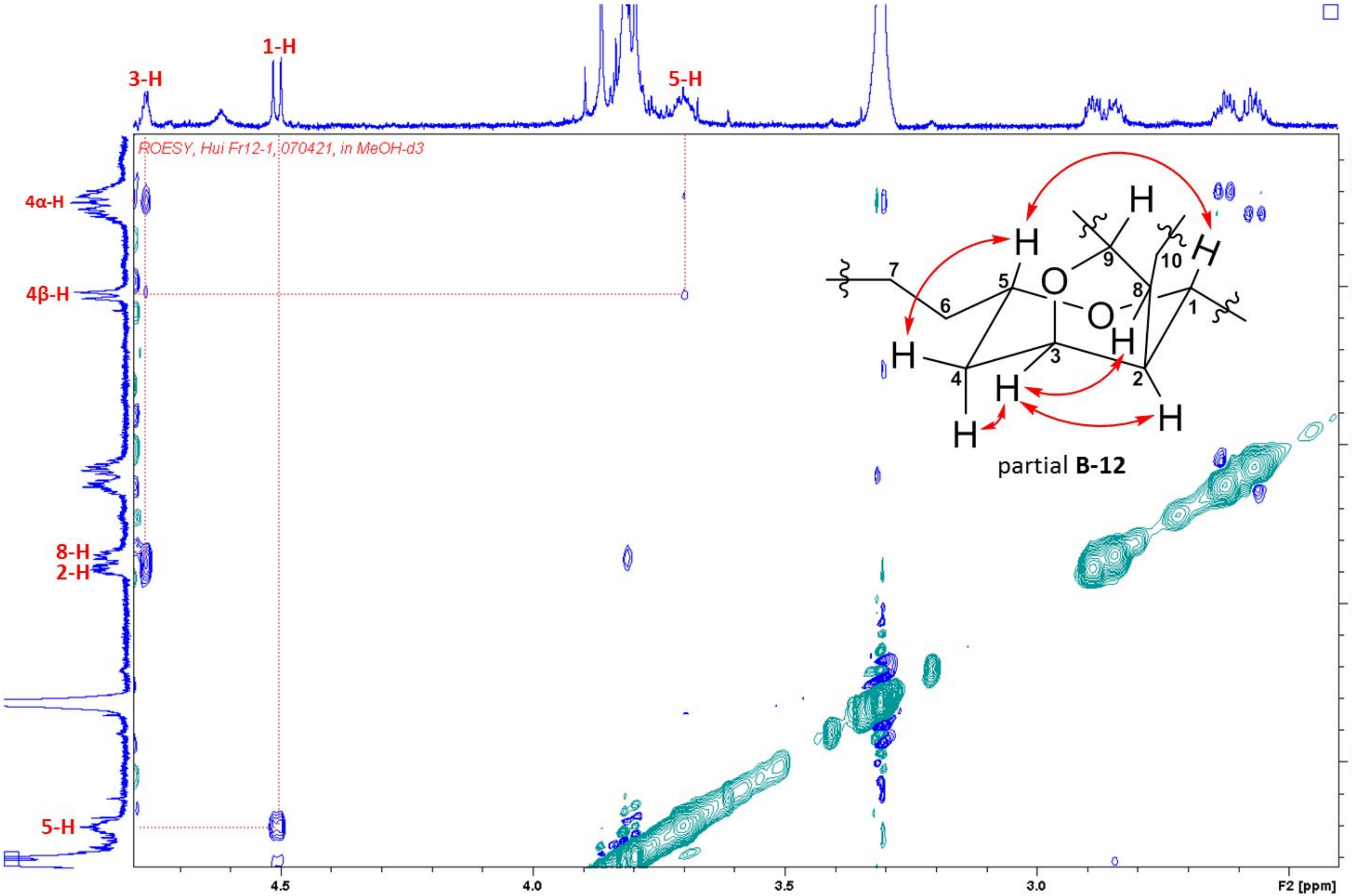

Figure S60. ROESY spectrum of compound **B-12** in CD_3OH.

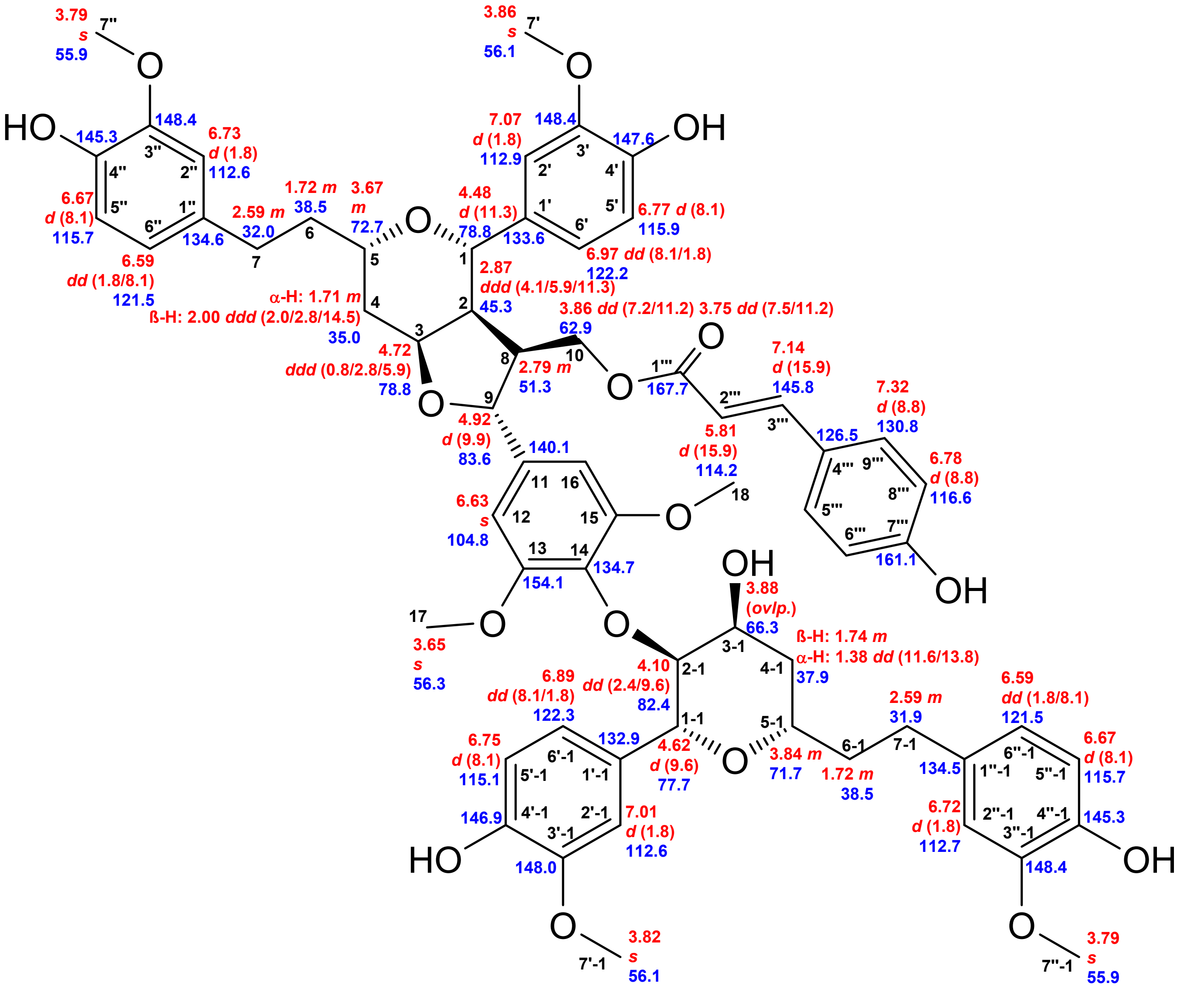

Figure S61. Chemical shifts of compound **B-15**. Red: ^{1}H chemical shifts (δ ppm, *mult.*, $^{3}J_{HH}$ in Hz). Blue: ^{13}C chemical shifts (δ ppm).

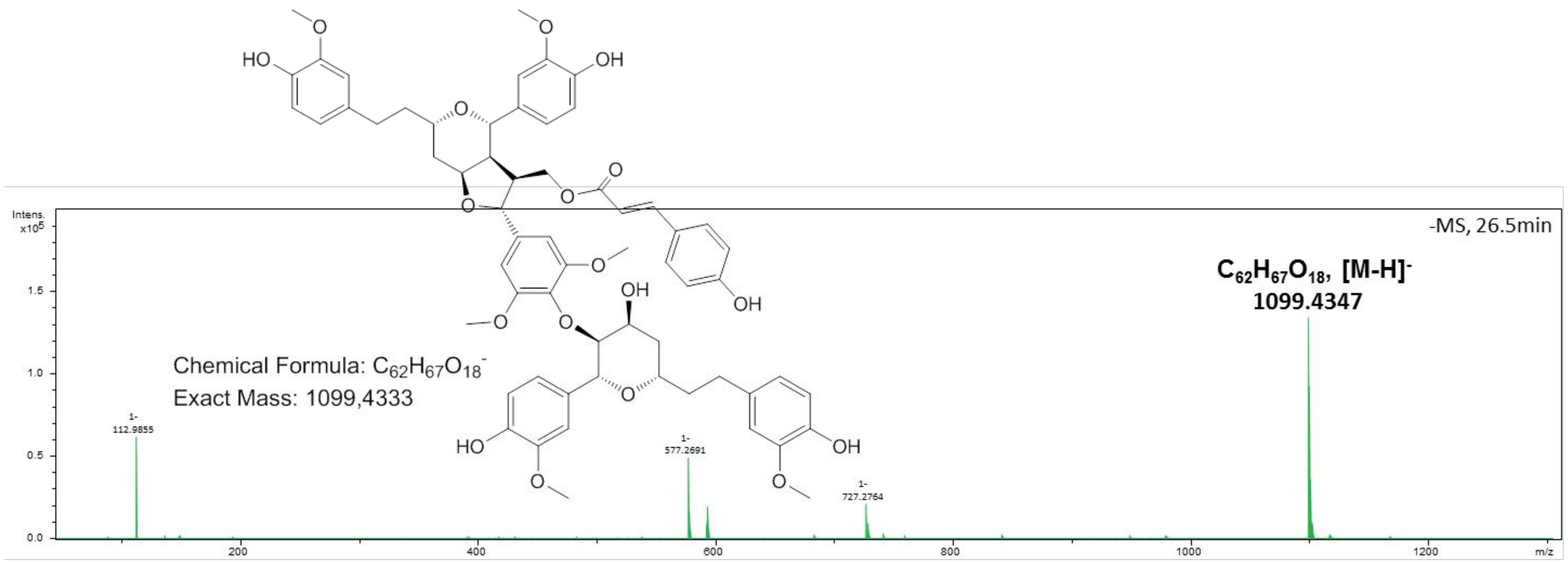

Figure S62. HR-ESI-MS spectrum of compound **B-15**.

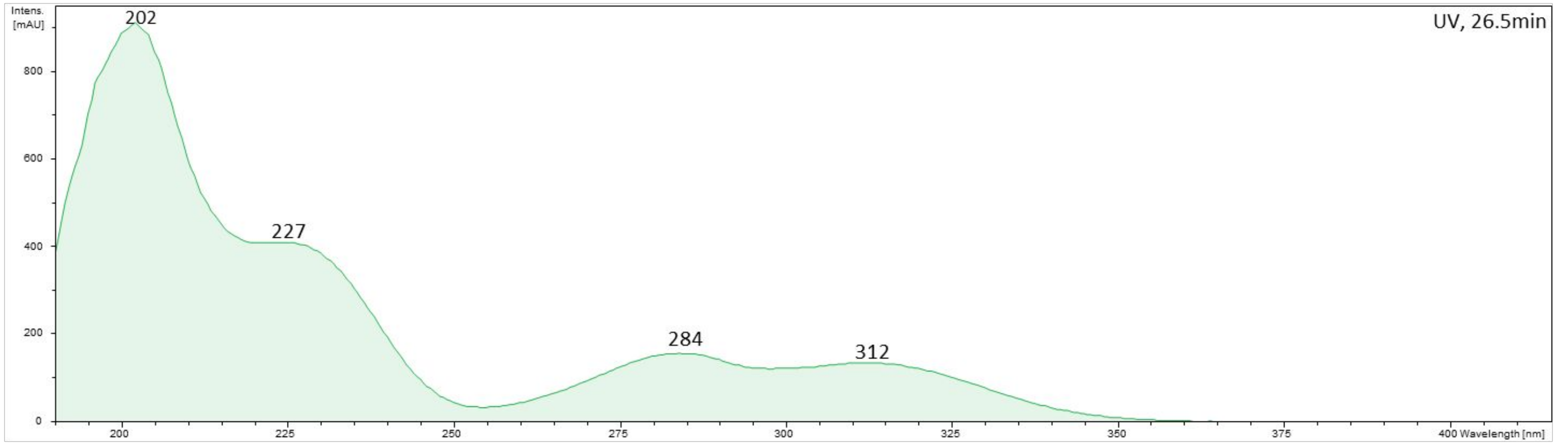

Figure S63. UV/Vis spectrum (MeCN-H_2O) of compound **B-15**.

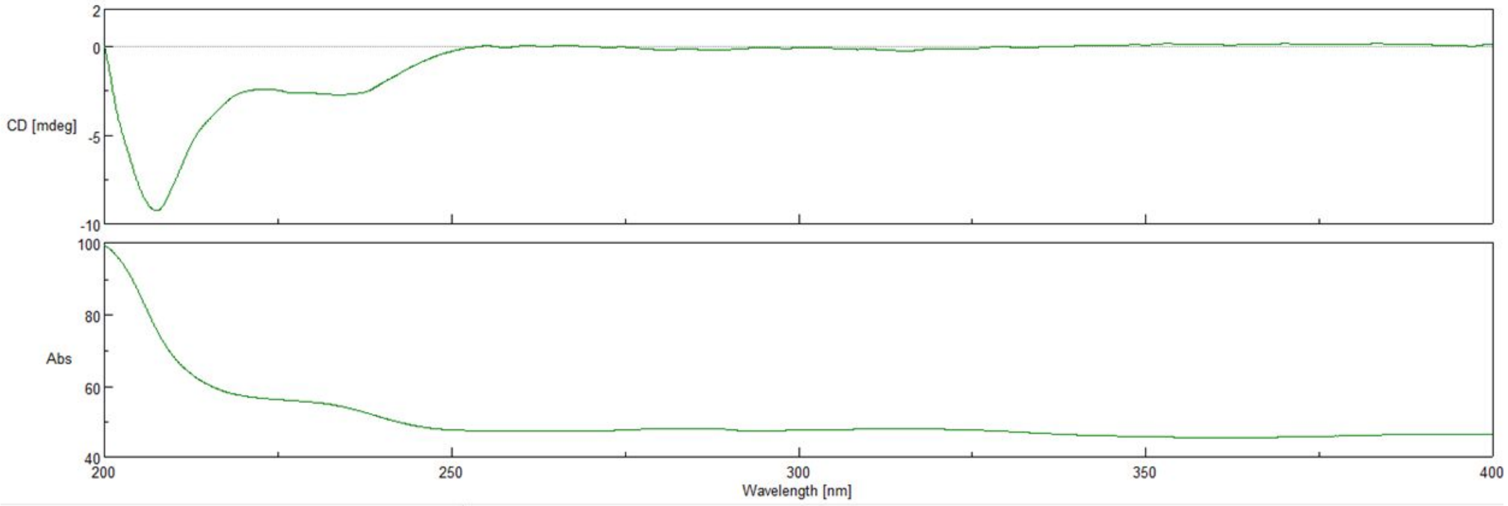

Figure S64. Experimental ECD (upper) and UV (lower) spectra (MeOH) of compound **B-15**.

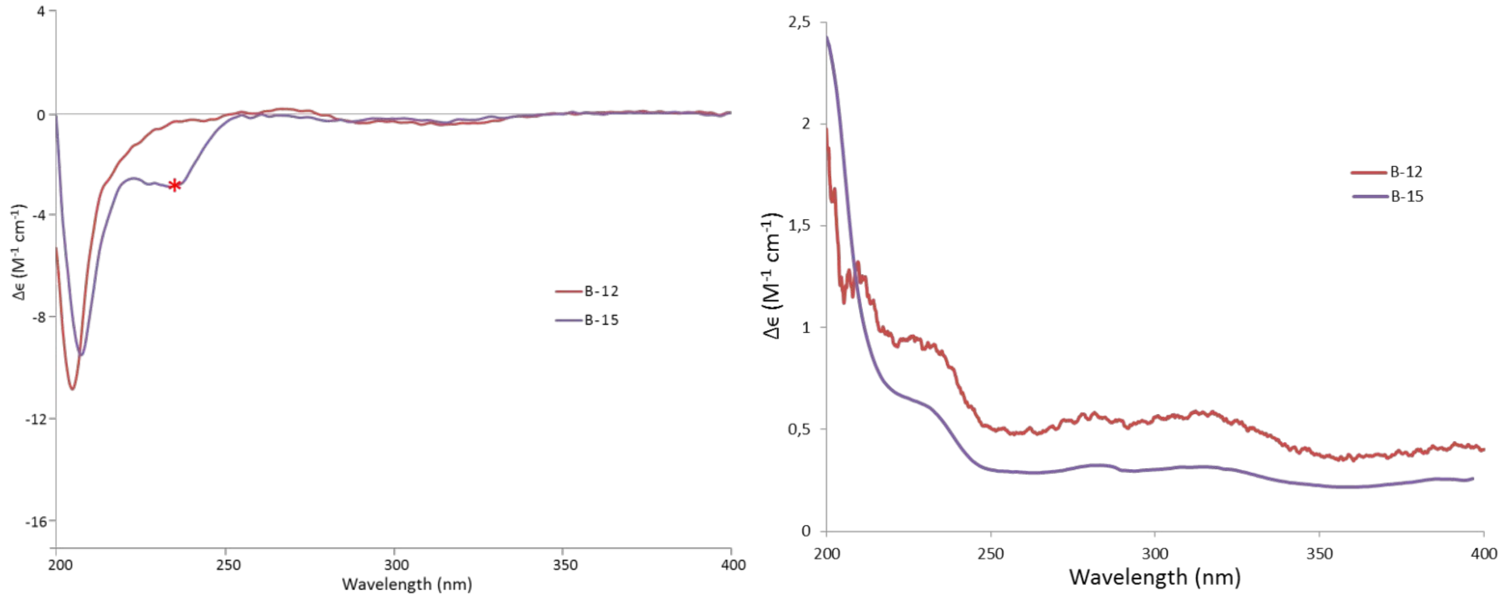

Figure S65. Experimental ECD (left) and UV (right) spectra of compounds **B-12** and **B-15**.

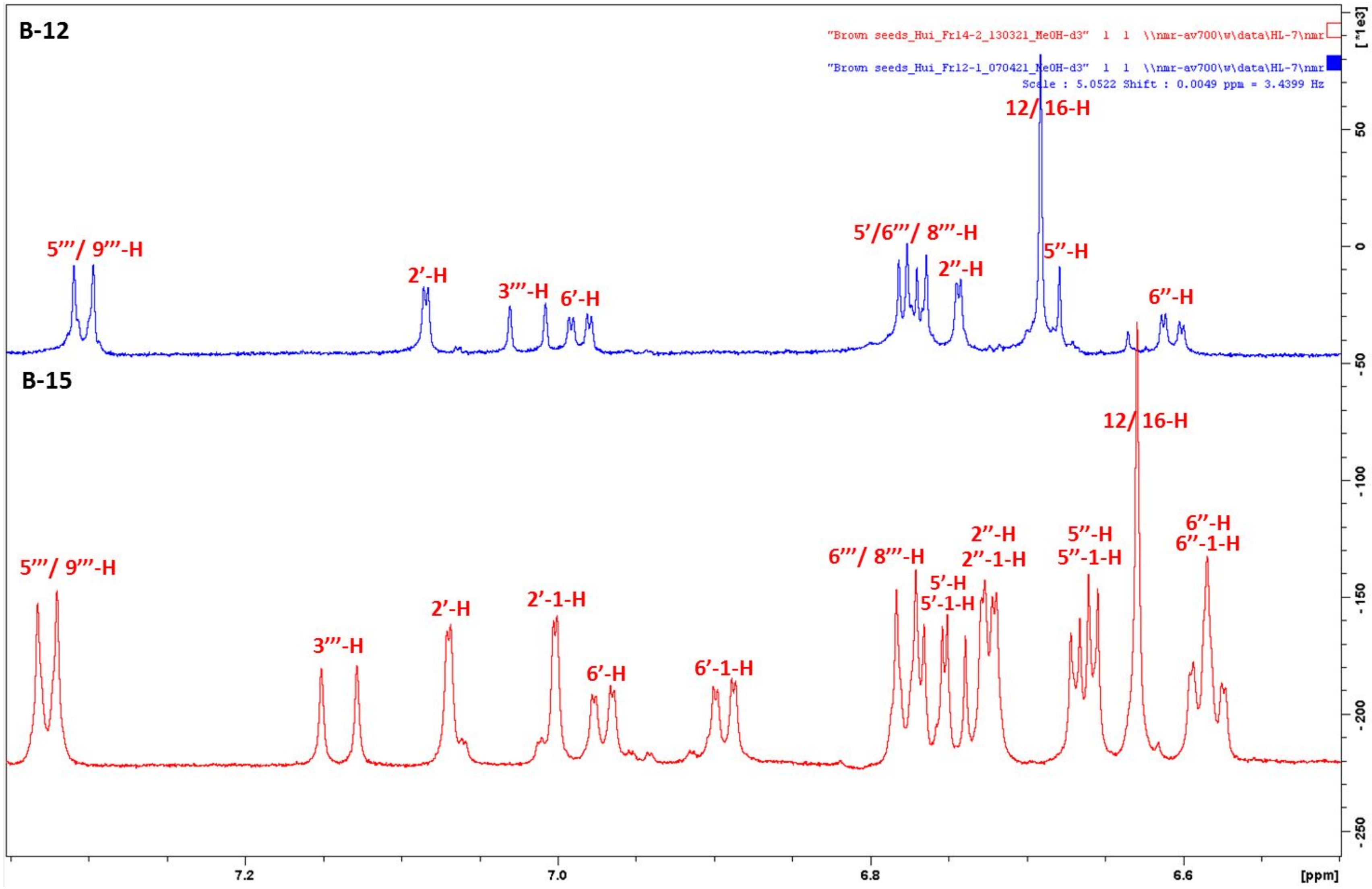

Figure S66. Detail of the ^{1}H NMR spectra (700 MHz, CD_3OH) of compound **B-12** (above) and compound **B-15** (below) (part-1).

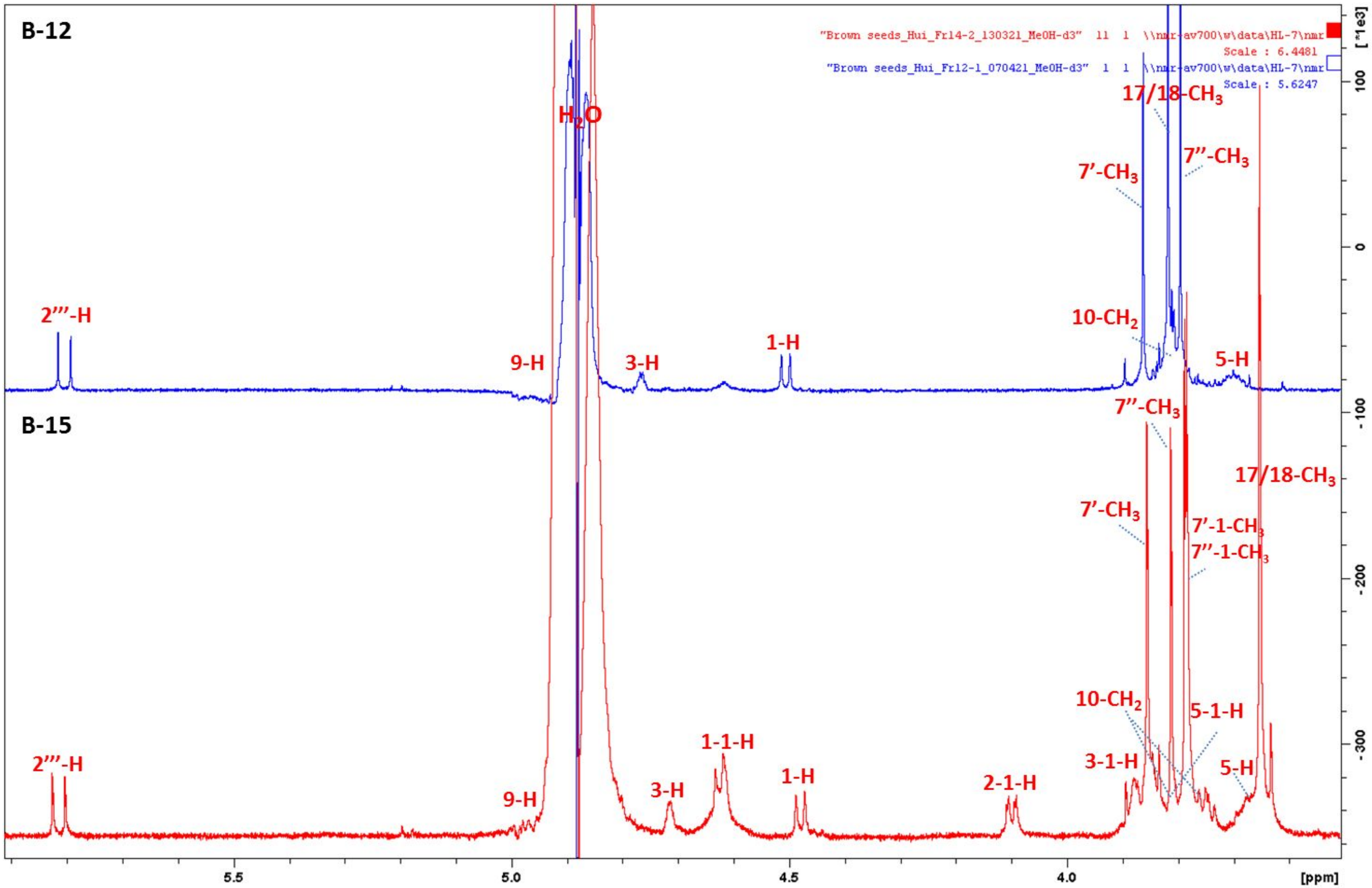

Figure S67. Detail of the ^{1}H NMR spectra (700 MHz, CD_3OH) of compound **B-12** (above) and compound **B-15** (below) (part-2).

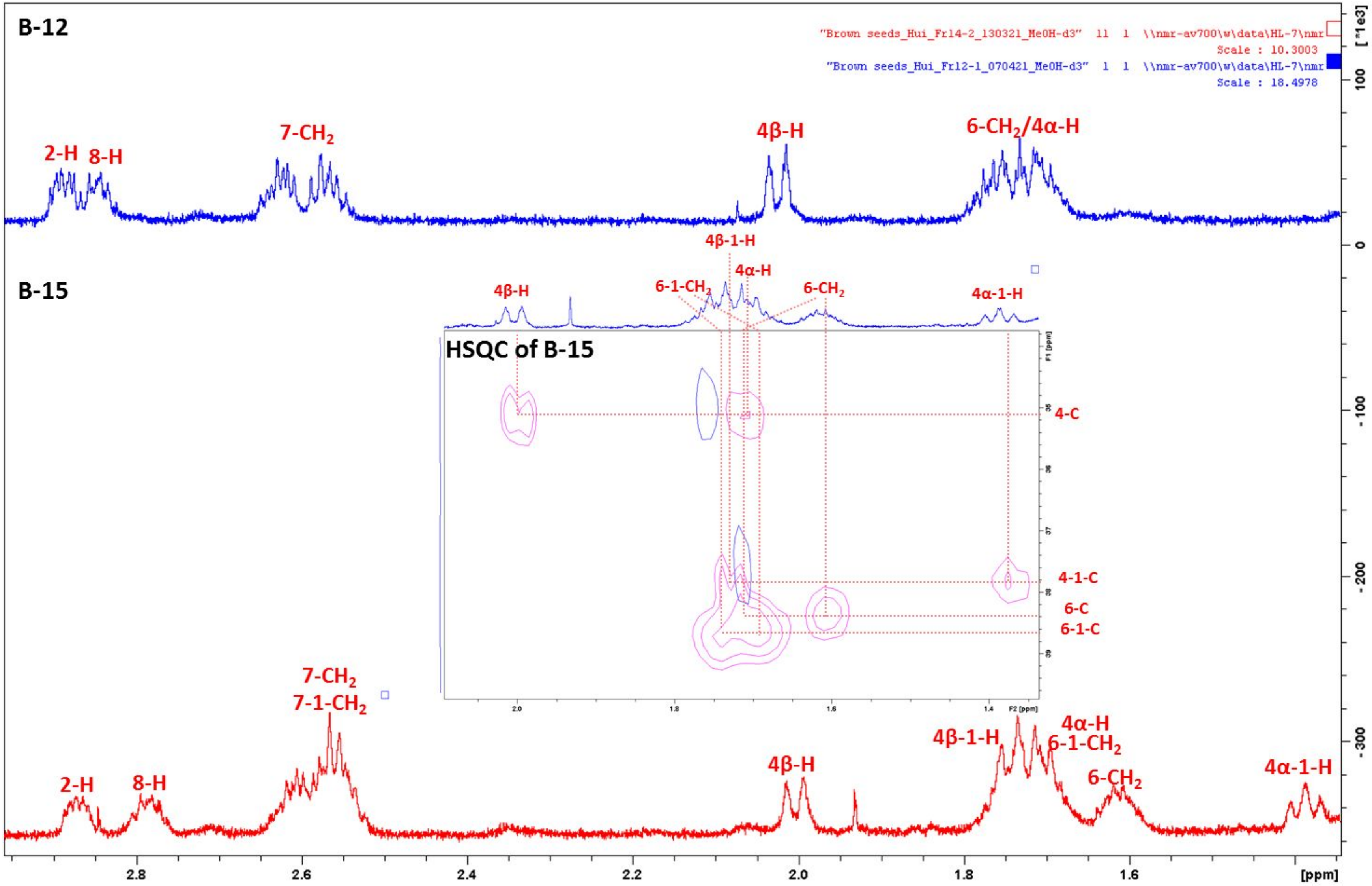

Figure S68. Detail of the ^{1}H NMR spectra (700 MHz, CD_3OH) of compound **B-12** (above) and compound **B-15** (below) (part-3).

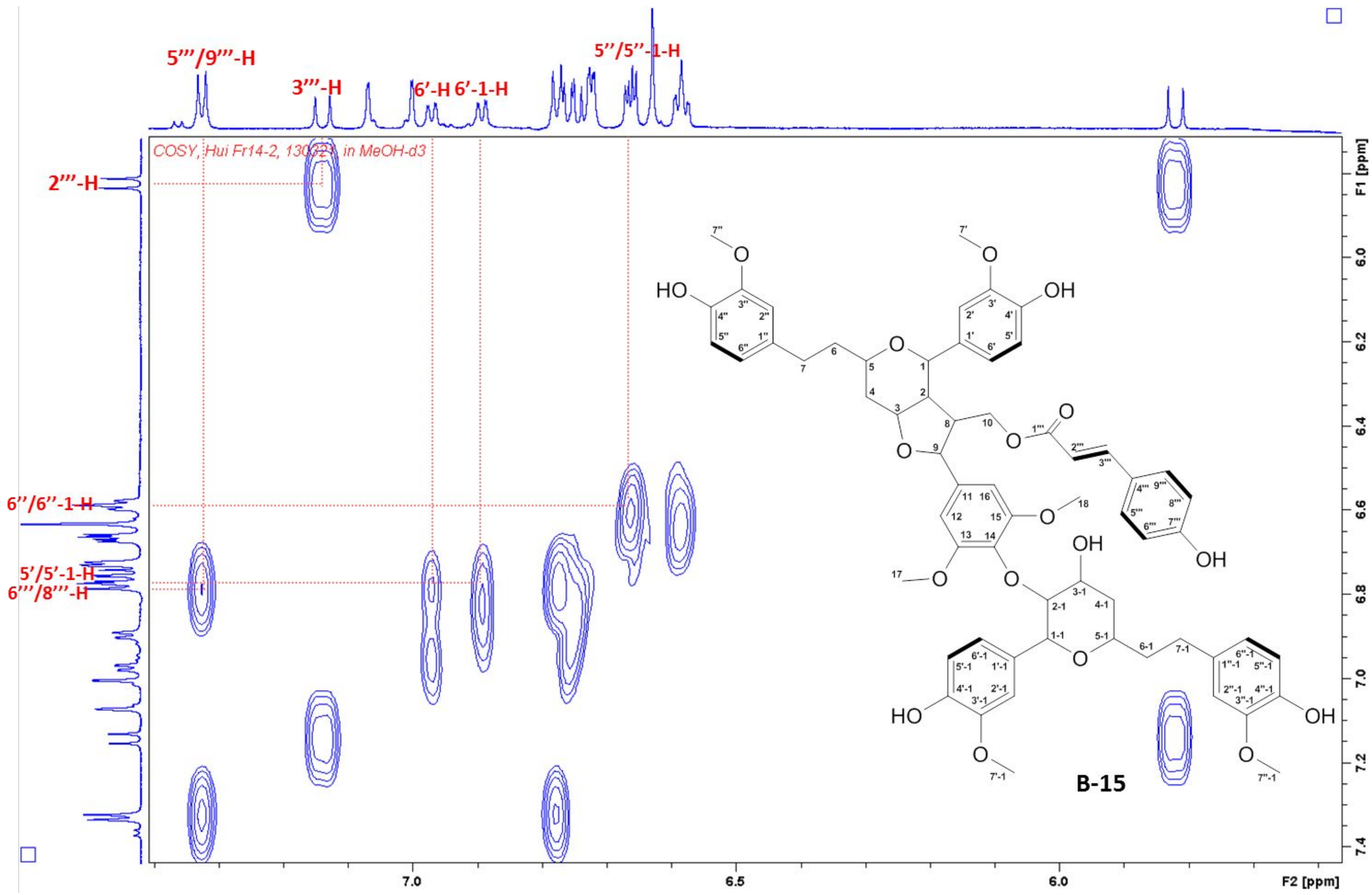

Figure S69. ^{1}H–^{1}H COSY spectrum of compound **B-15** in CD_3OH (part-1).

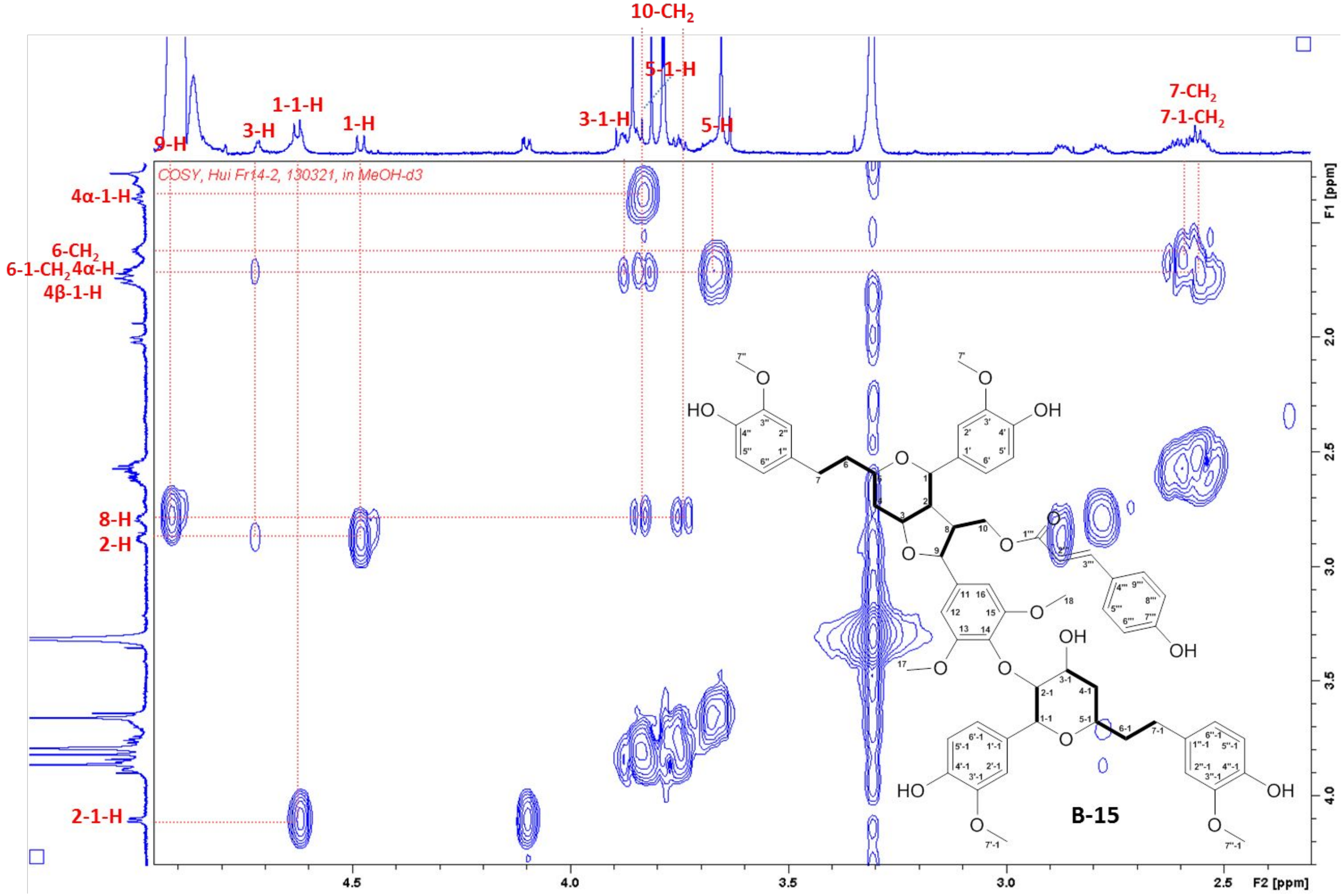

Figure S70. ^1H–^1H COSY spectrum of compound **B-15** in CD_3OH (part-2).

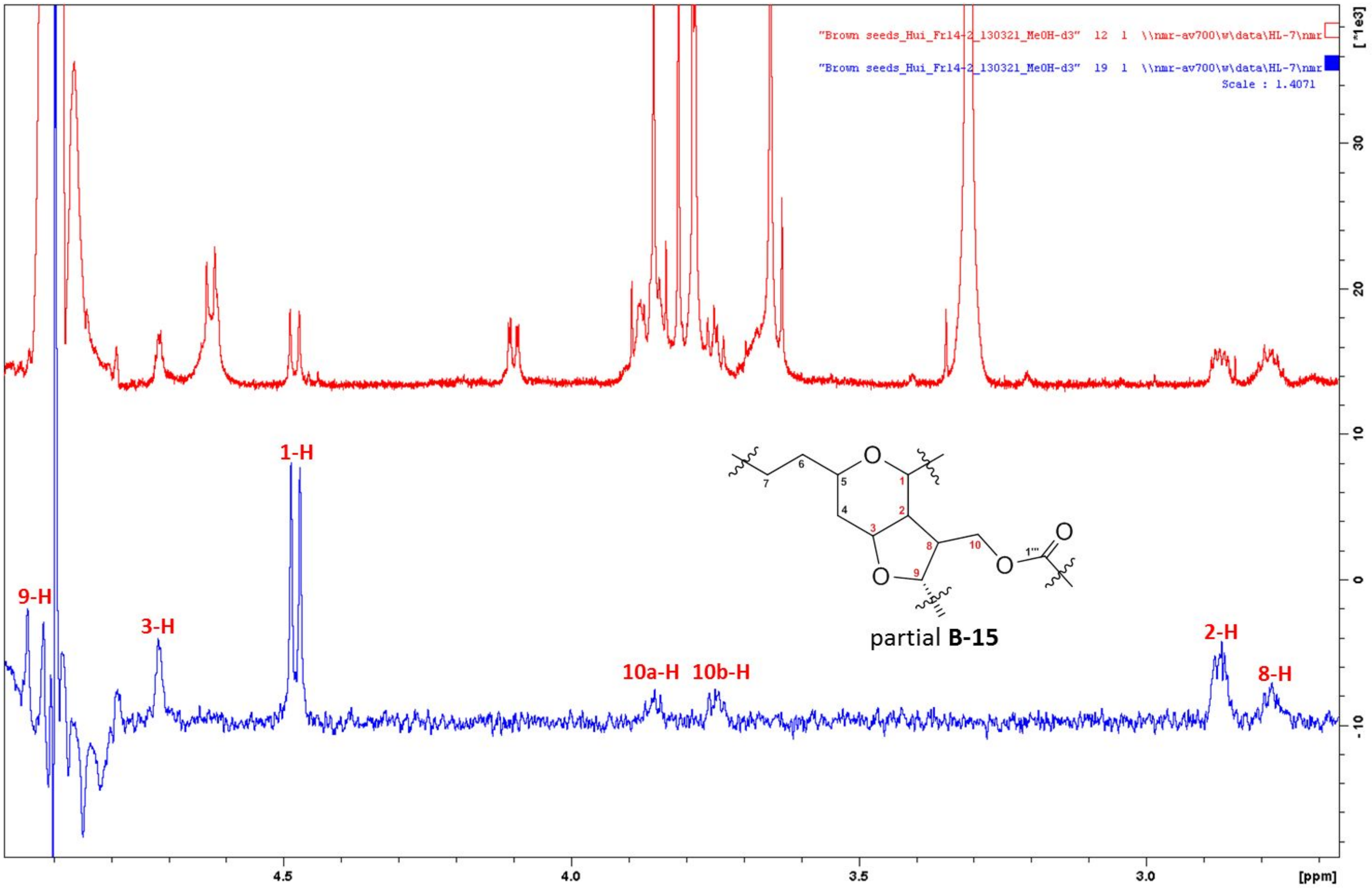

Figure S71. ^{1}H NMR spectrum (above) and sel-TOCSY spectrum (below, transmitter frequency at H-2, δ_H 2.87 ppm) of compound **B-15**. (in CD_3OH).

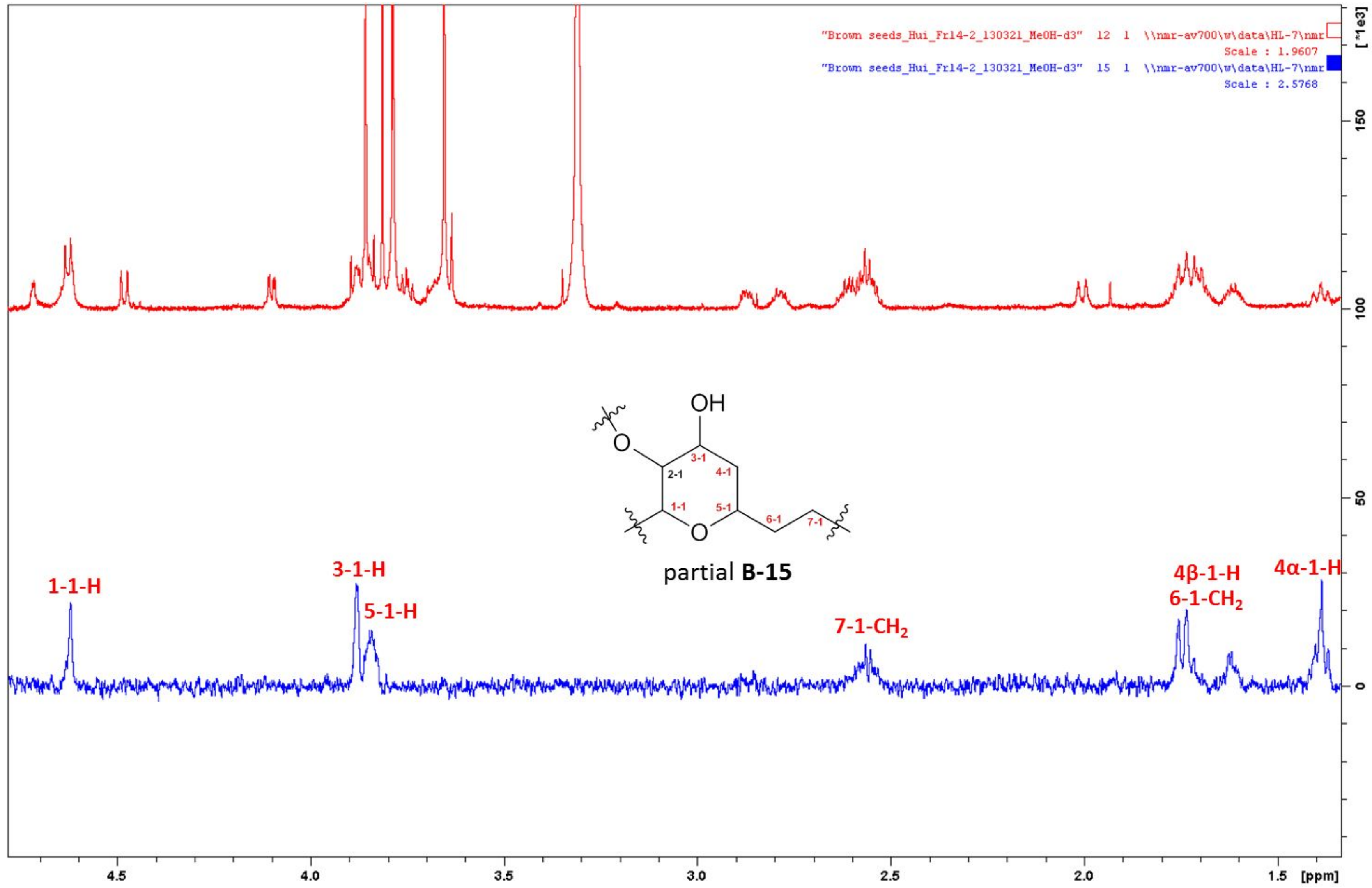

Figure S72. ^{1}H NMR spectrum (above) and sel-TOCSY spectrum (below, transmitter frequency at H-4α-1, δ_H 1.42 ppm) of compound **B-15**. (in CD_3OH).

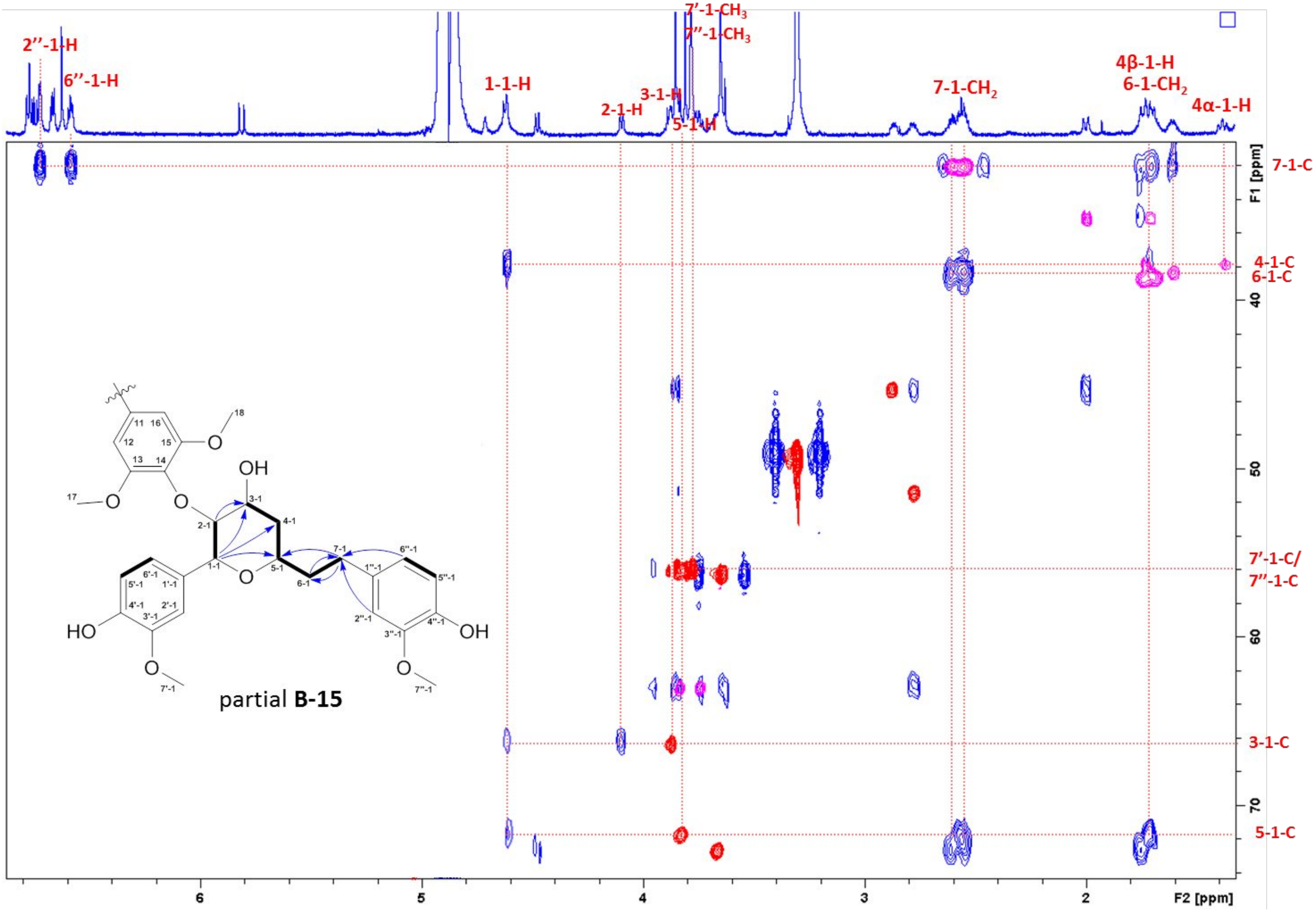

Figure S73. Superimposed HSQC and HMBC spectra of partial compound **B-15** in CD_3OH (part-1).

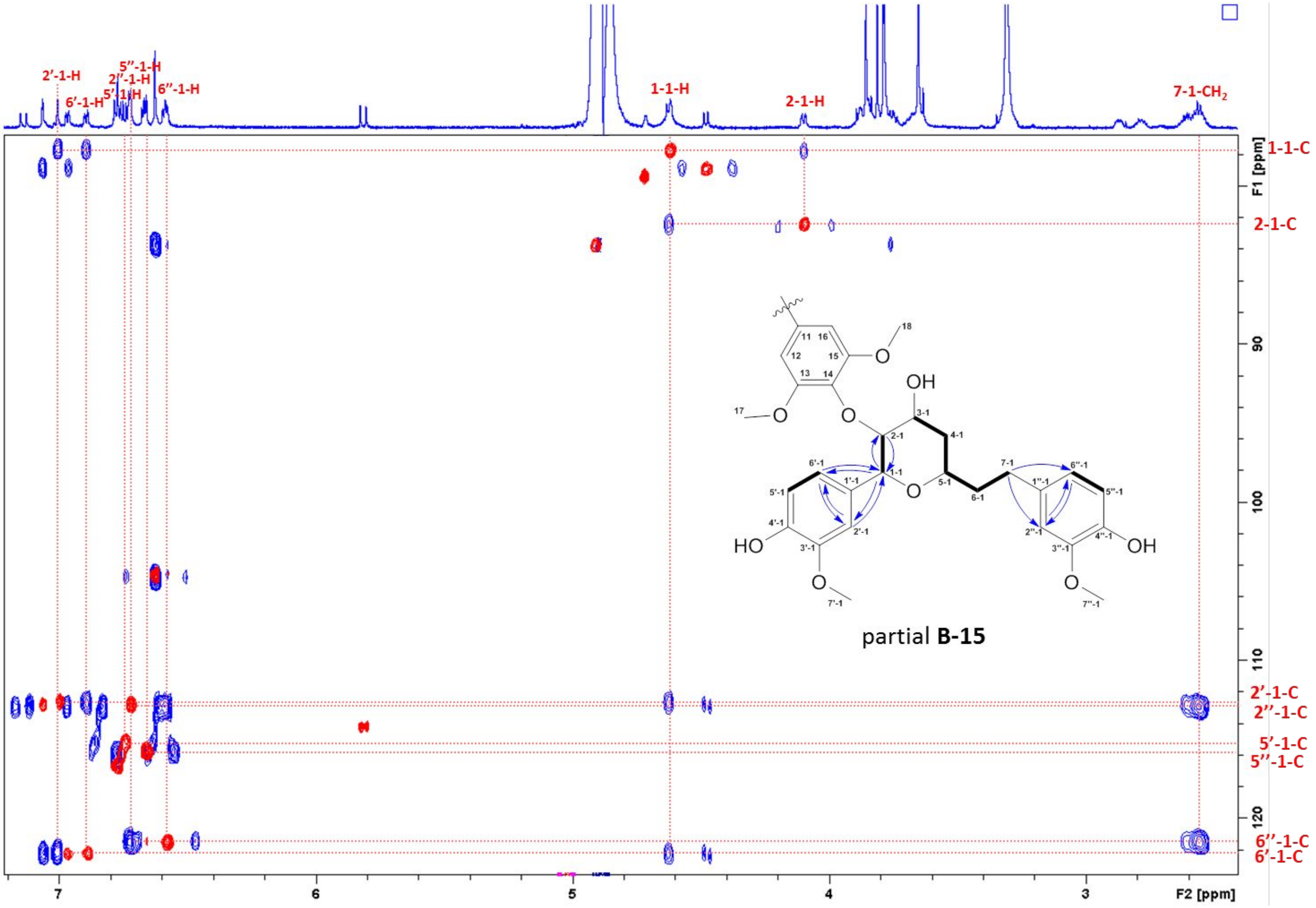

Figure S74. Superimposed HSQC and HMBC spectra of partial compound **B-15** in CD_3OH (part-2).

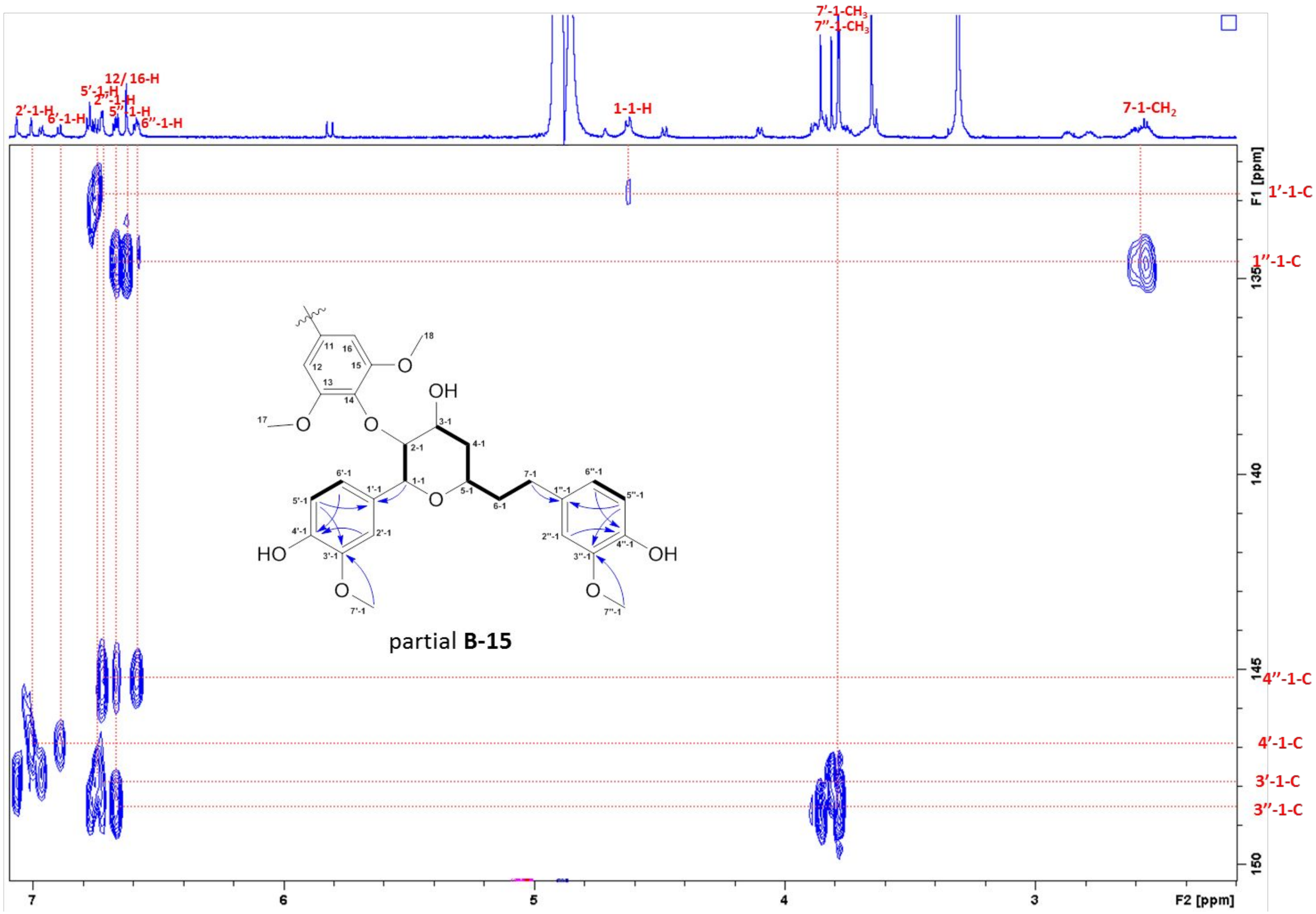

Figure S75. Superimposed HSQC and HMBC spectra of partial compound **B-15** in CD_3OH (part-3).

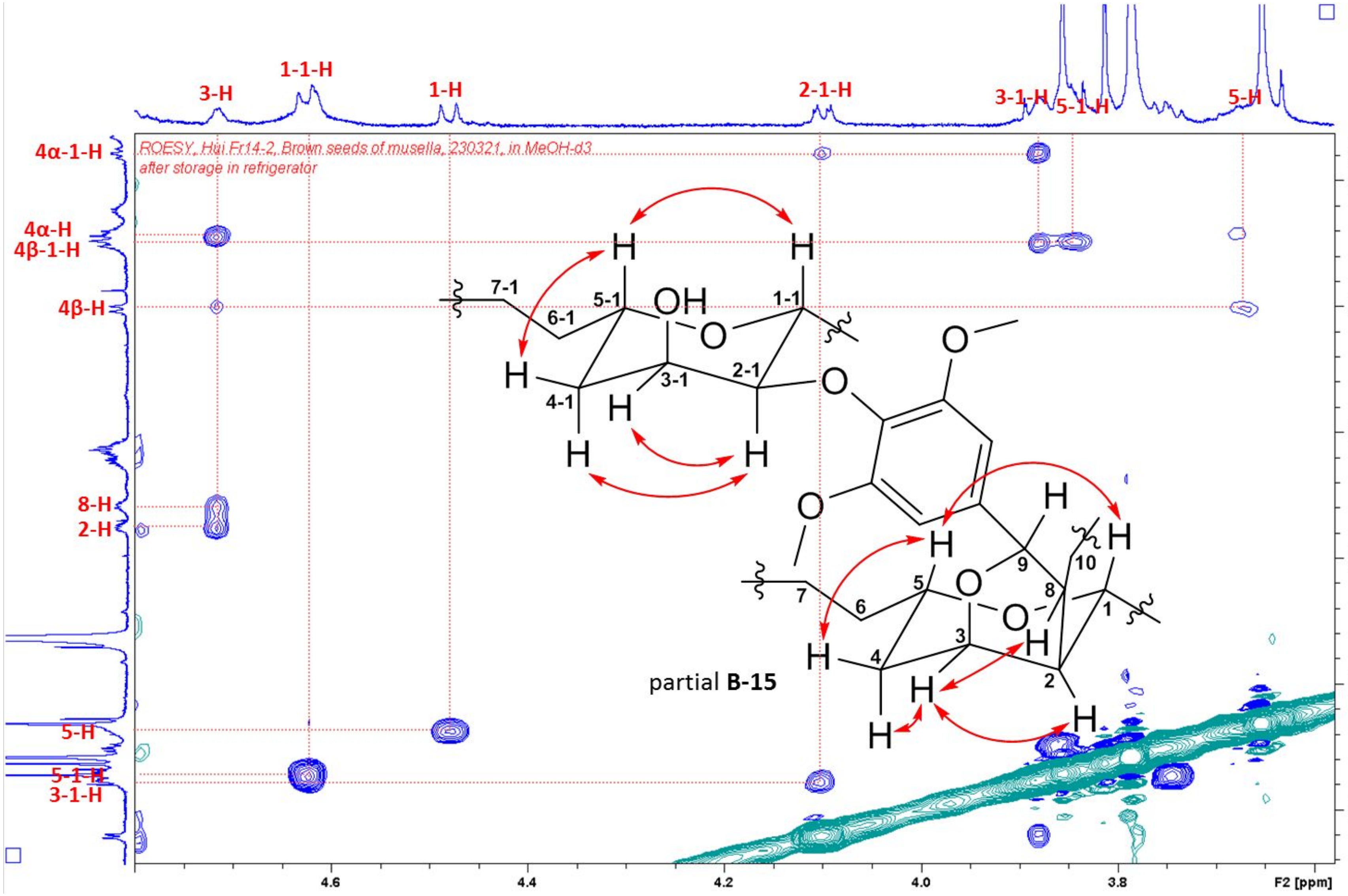

Figure S76. ROESY spectrum of compound **B-15** in CD_3OH.

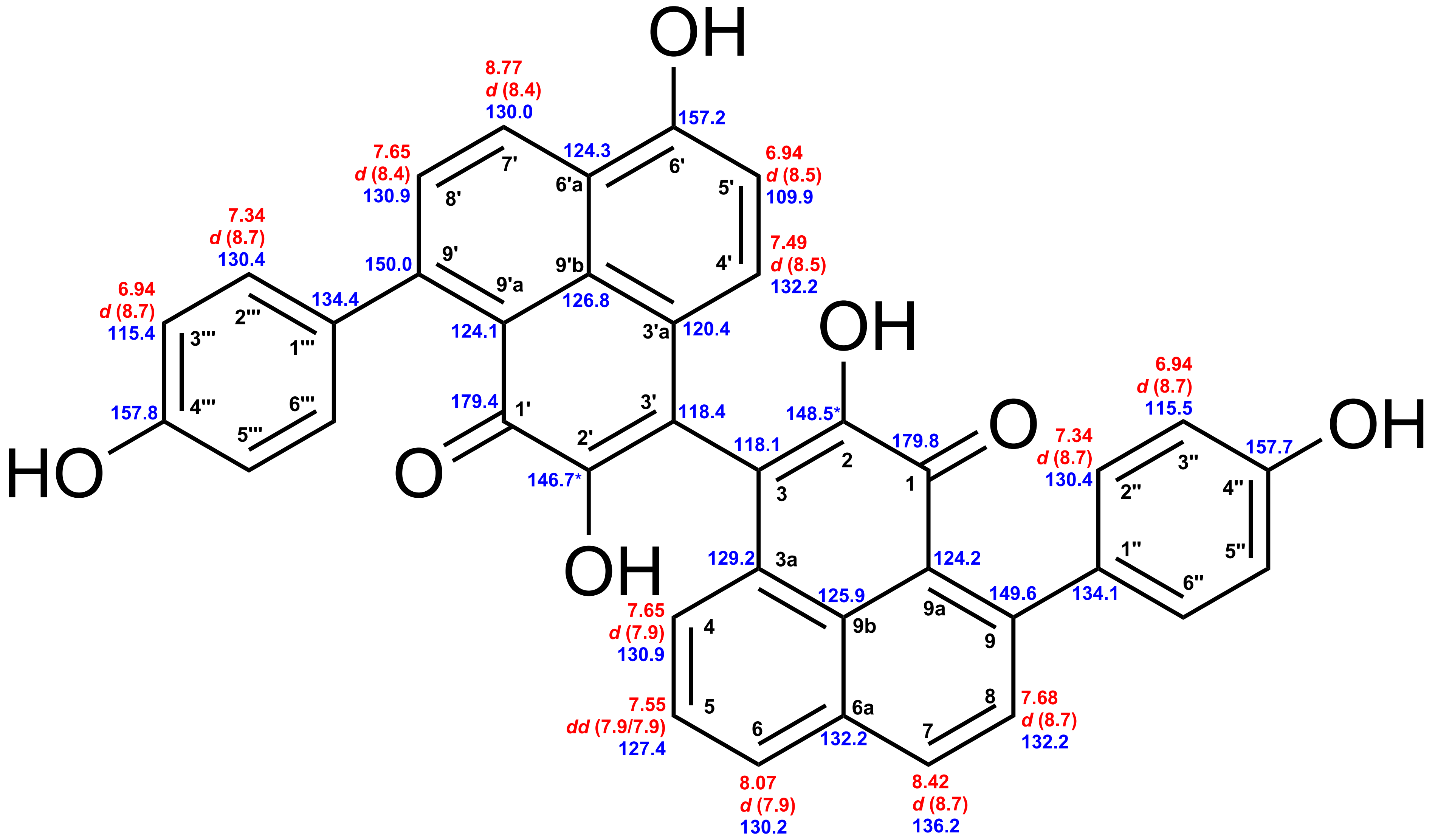

Figure S77. Chemical shifts of compound **M-6**.
Red: [1]H chemical shifts (δ ppm, *mult*., $^{3}J_{HH}$ in Hz). Blue: [13]C chemical shifts (δ ppm). *signals may be switched.

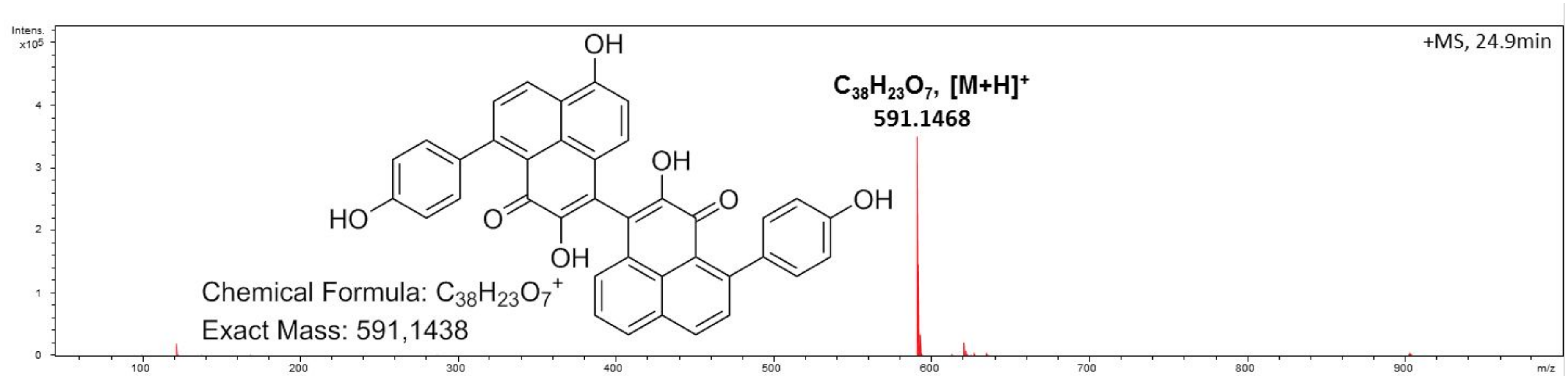

Figure S78. HR-ESI-MS spectrum of compound **M-6**.

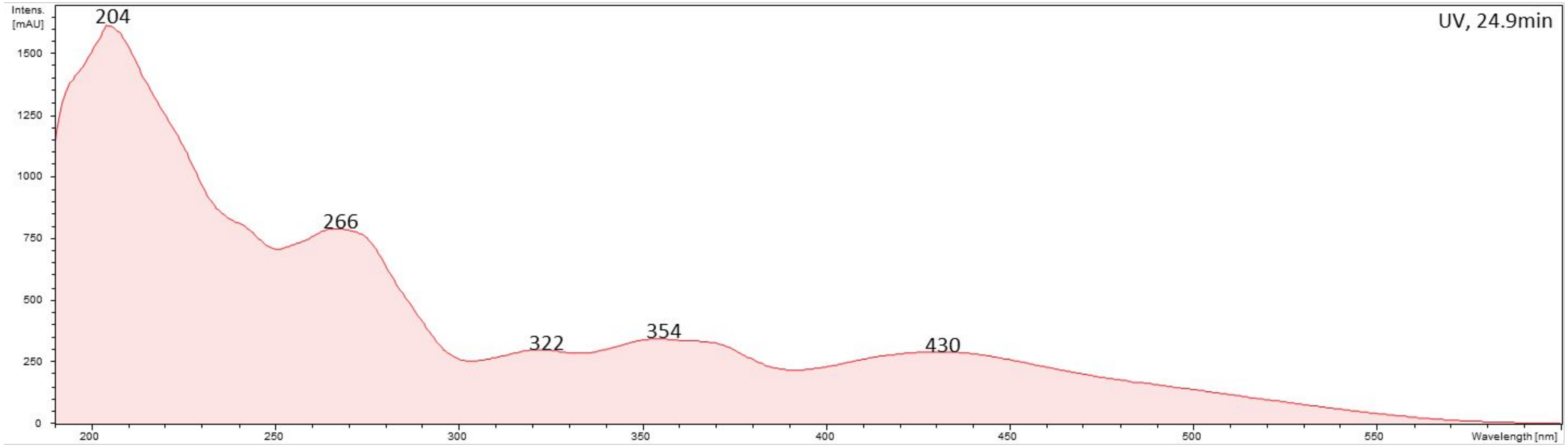

Figure S79. UV/Vis spectrum (MeCN-H_2O) of compound **M-6**.

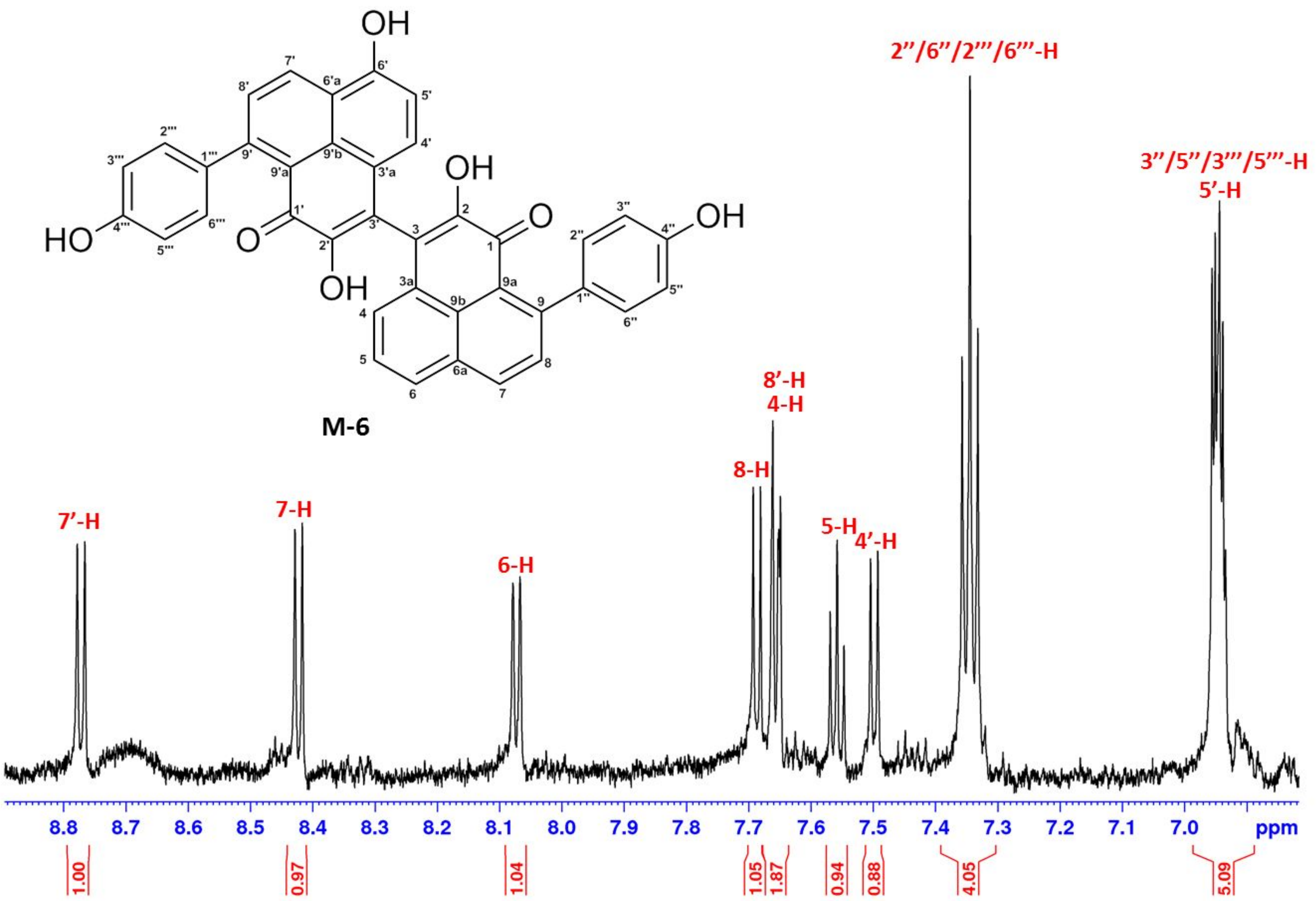

Figure S80. ^{1}H NMR spectrum (700 MHz, acetone-d$_6$) of compound **M-6**.

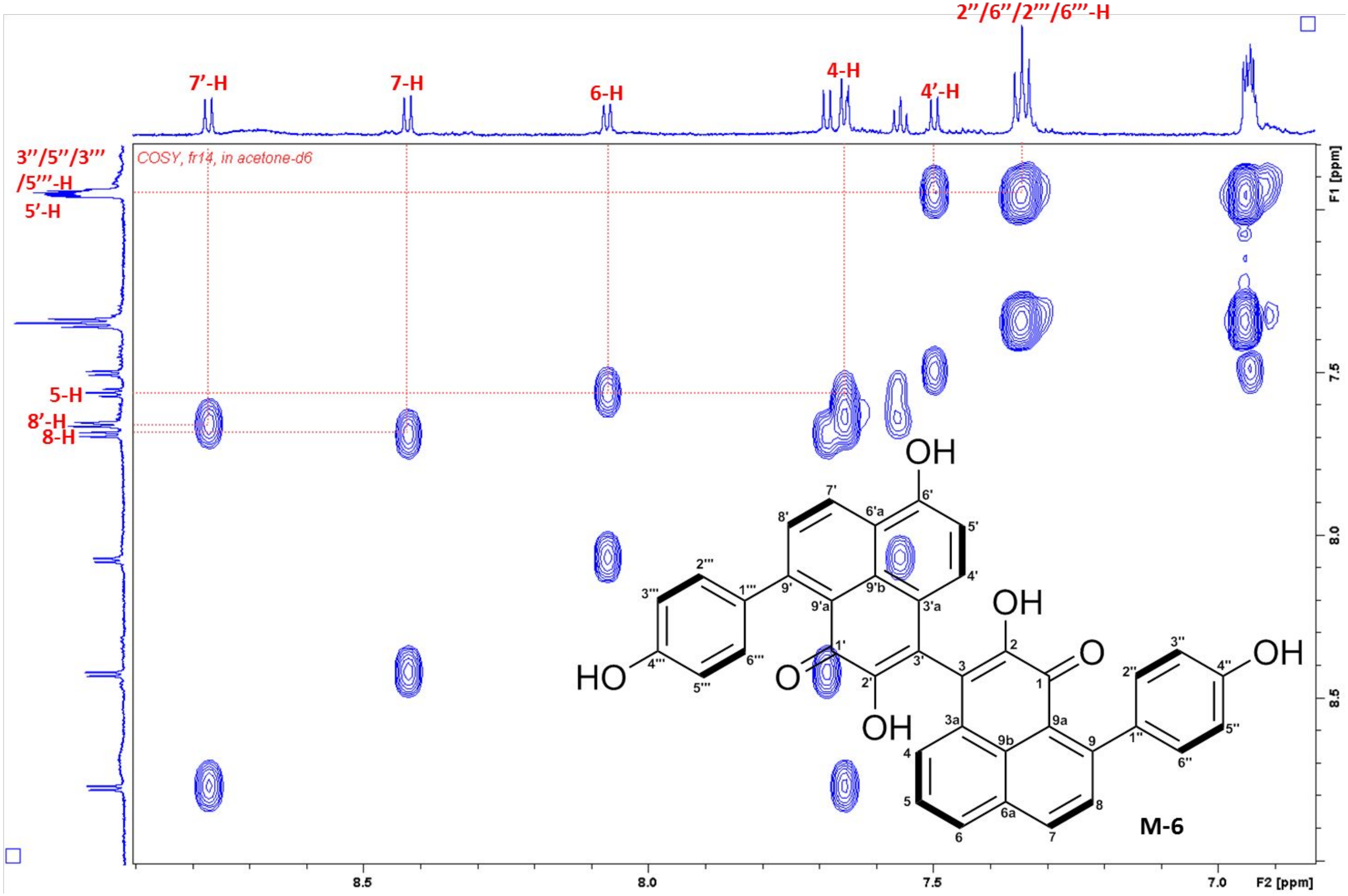

Figure S81. ^{1}H–^{1}H COSY spectrum of compound **M-6** in acetone-d$_6$.

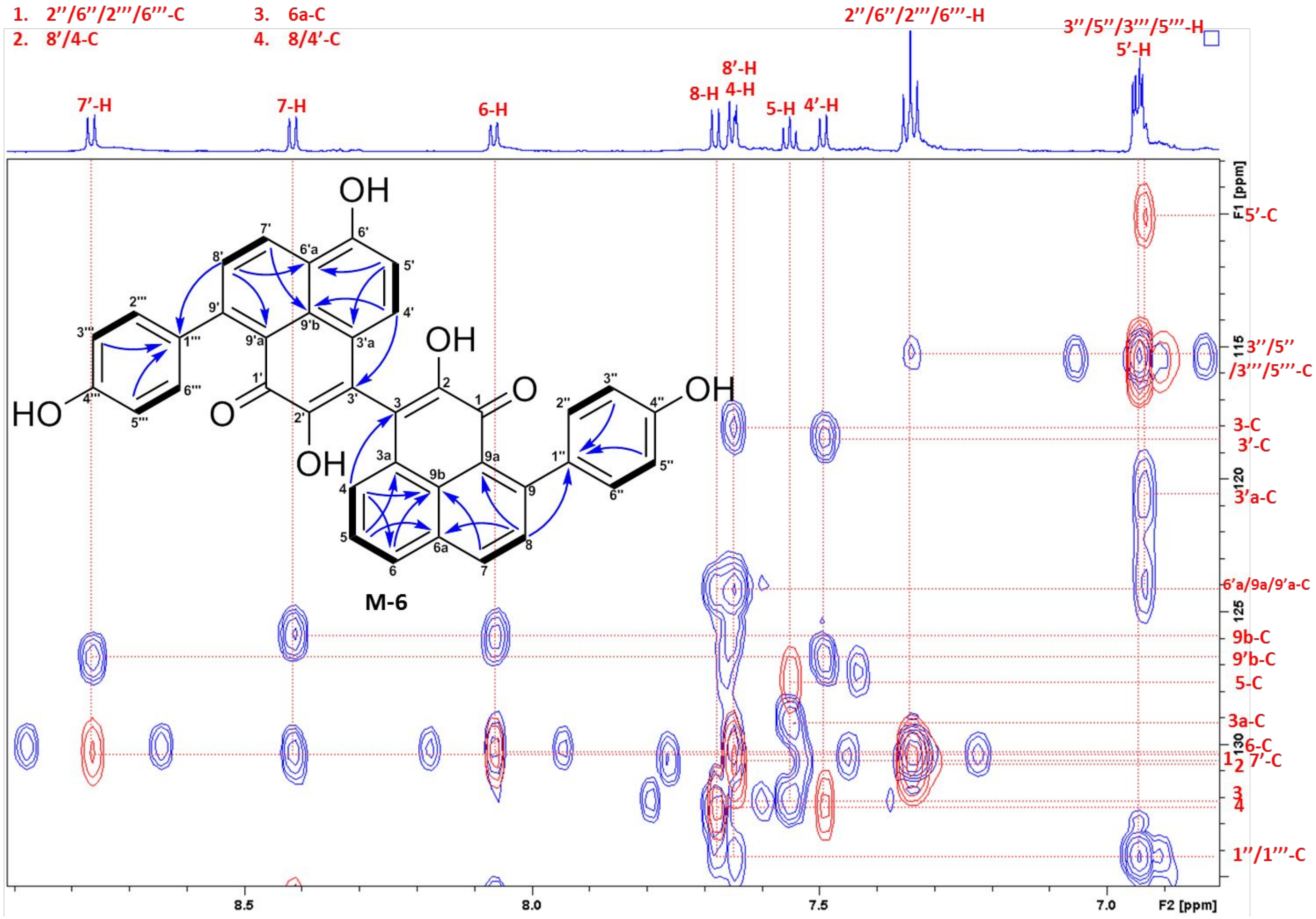

Figure S82. Superimposed HSQC and HMBC spectra of compound **M-6** in acetone-d_6 (part-1).

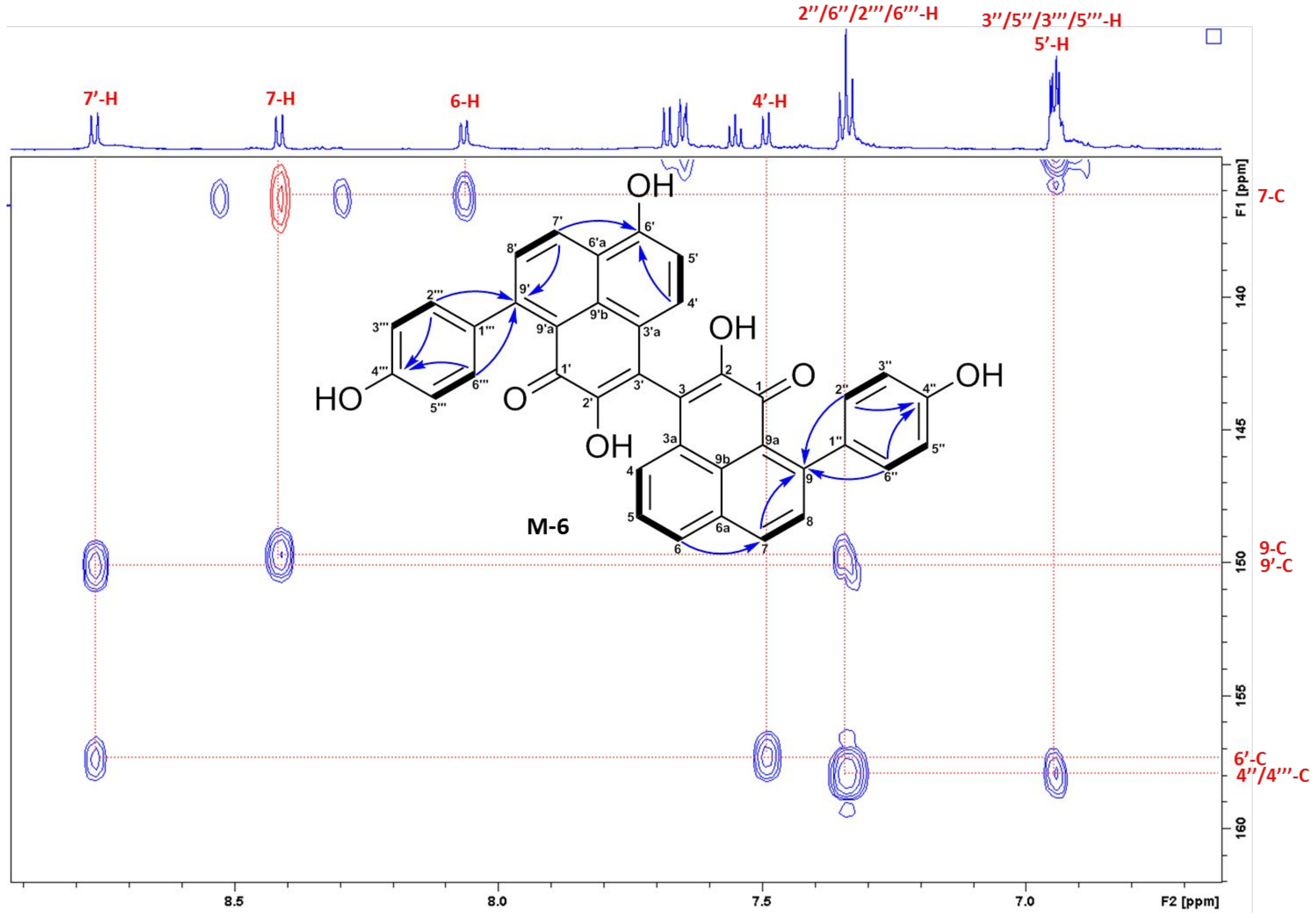

Figure S83. Superimposed HSQC and HMBC spectra of compound **M-6** in acetone-d_6 (part-2).

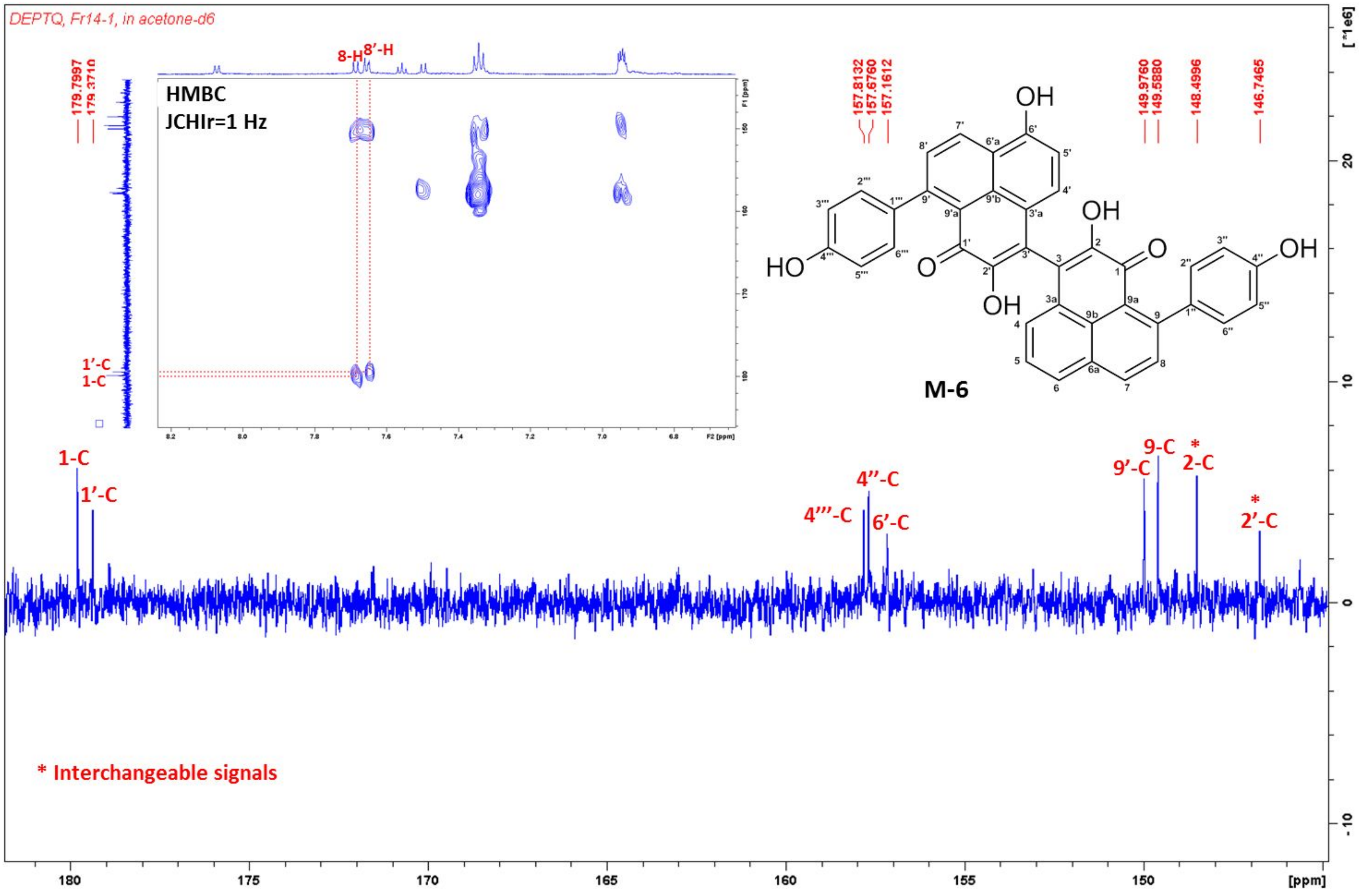

Figure S84. HMBC spectrum (J_{CH} = 1 Hz, acetone-d_6) and DEPTQ spectrum (175 MHz, acetone-d_6, part-1) of compound **M-6**.

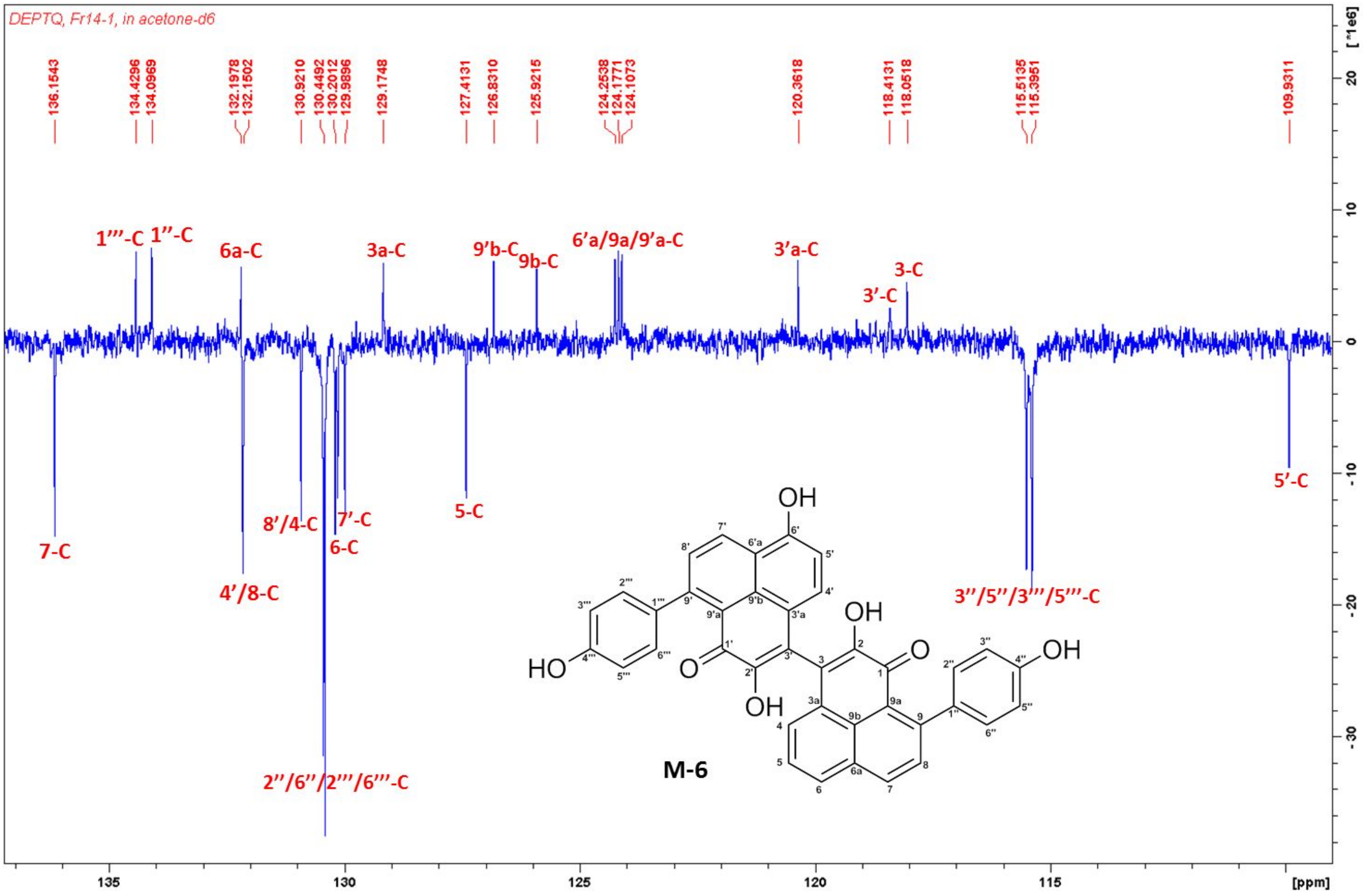

Figure S85. DEPTQ spectrum (175 MHz, acetone-d_6) of compound **M-6** (part-2).

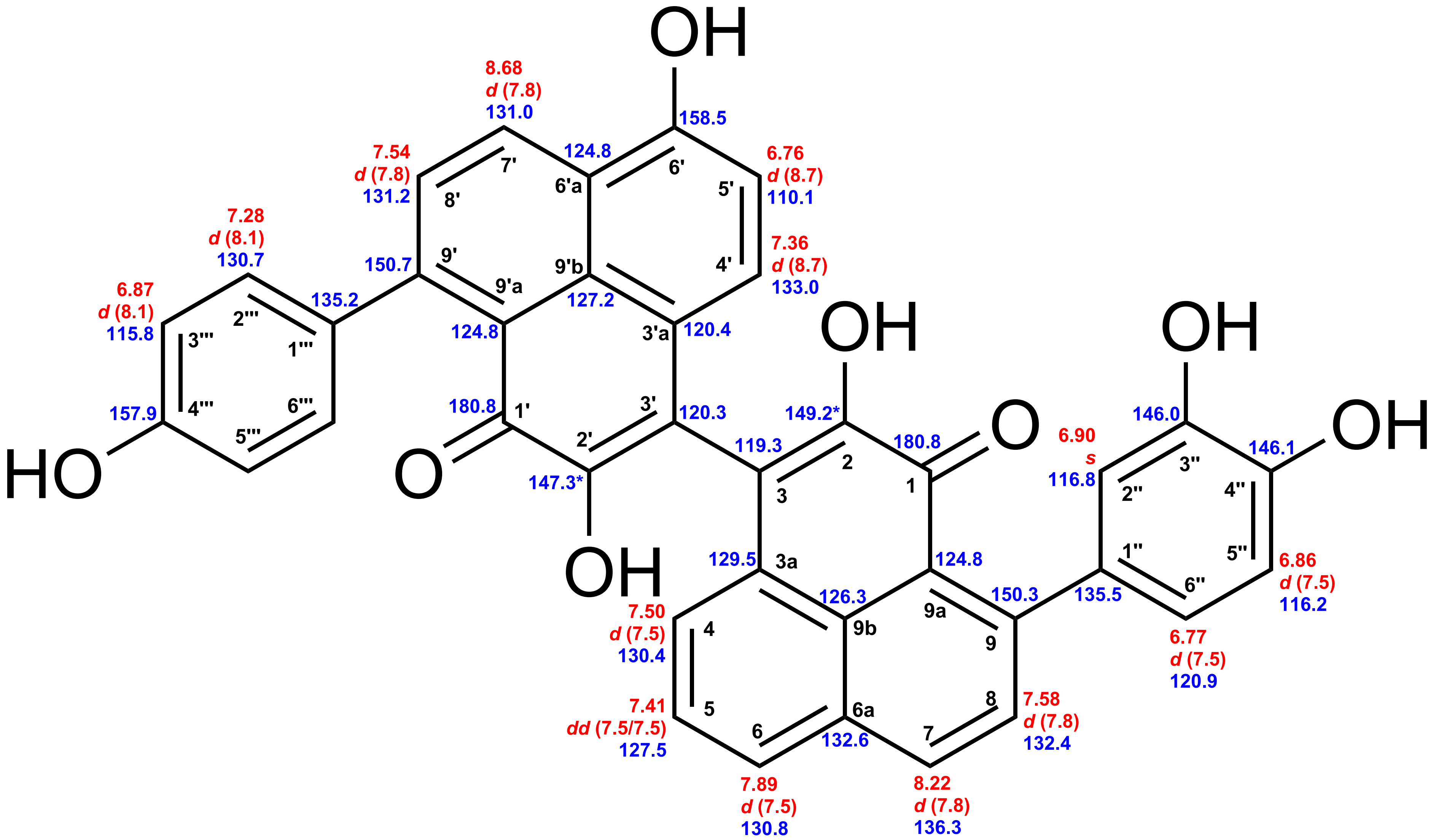

Figure S86. Chemical shifts of compound **M-4**.
Red: ^{1}H chemical shifts (δ ppm, *mult*., $^{3}J_{HH}$ in Hz). Blue: ^{13}C chemical shifts (δ ppm). * Signals may be switched.

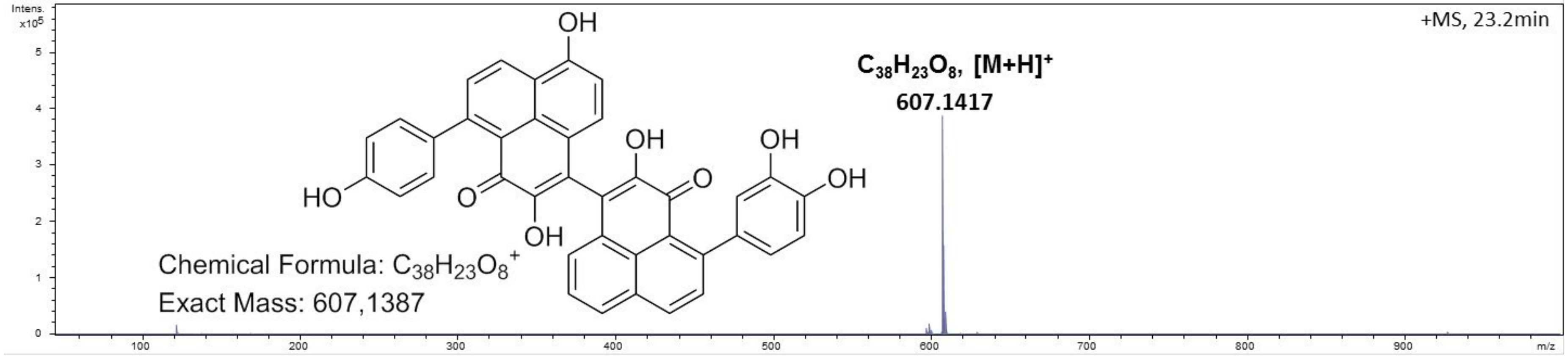

Figure S87. HR-ESI-MS spectrum of compound **M-4**.

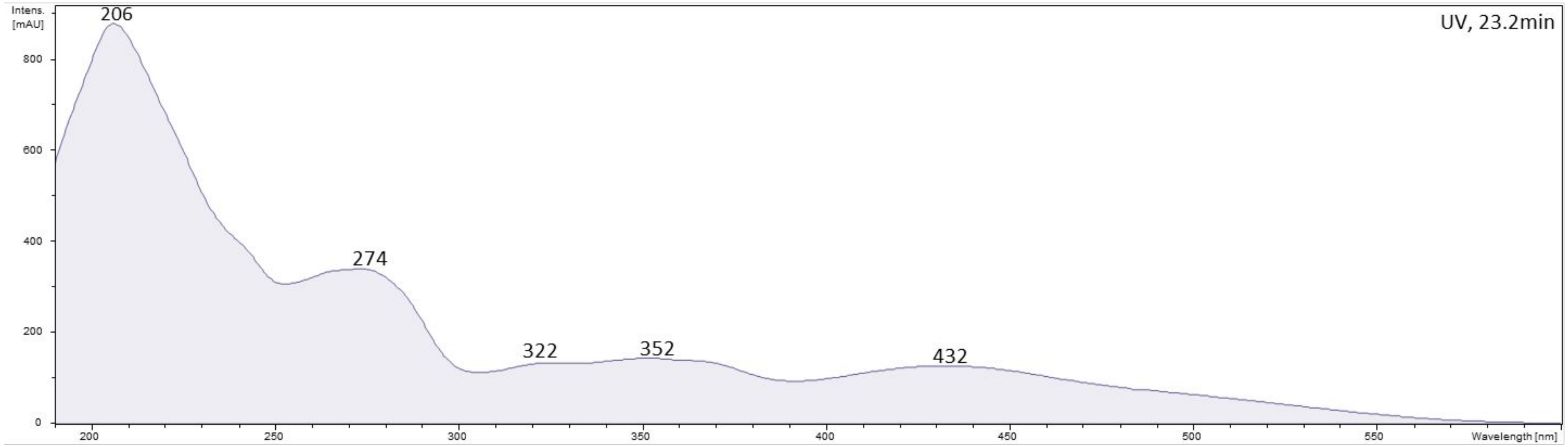

Figure S88. UV/Vis spectrum (MeCN-H_2O) of compound **M-4**.

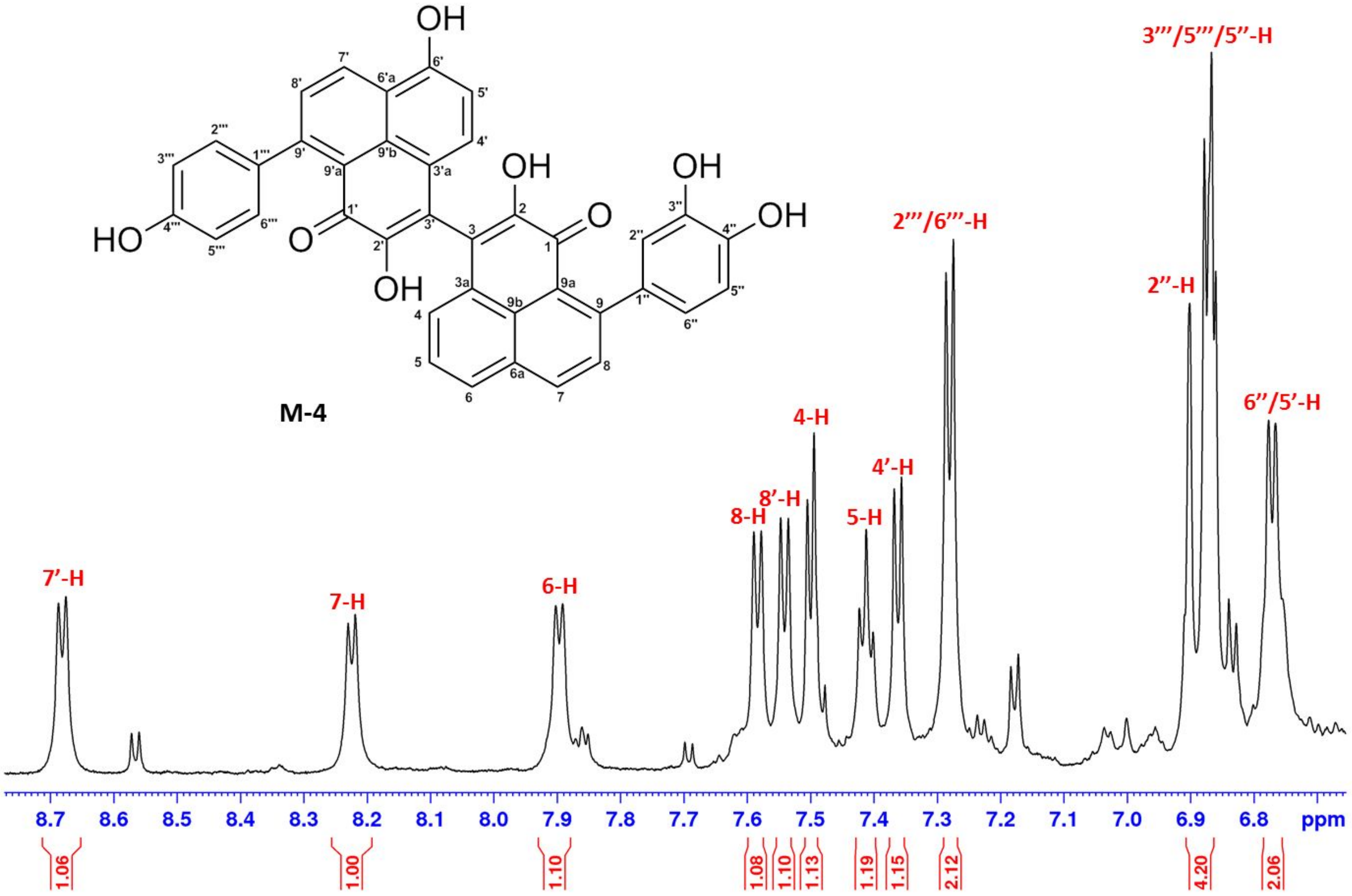

Figure S89. ^{1}H NMR spectrum (700 MHz, CD_3OH) of compound **M-4**.

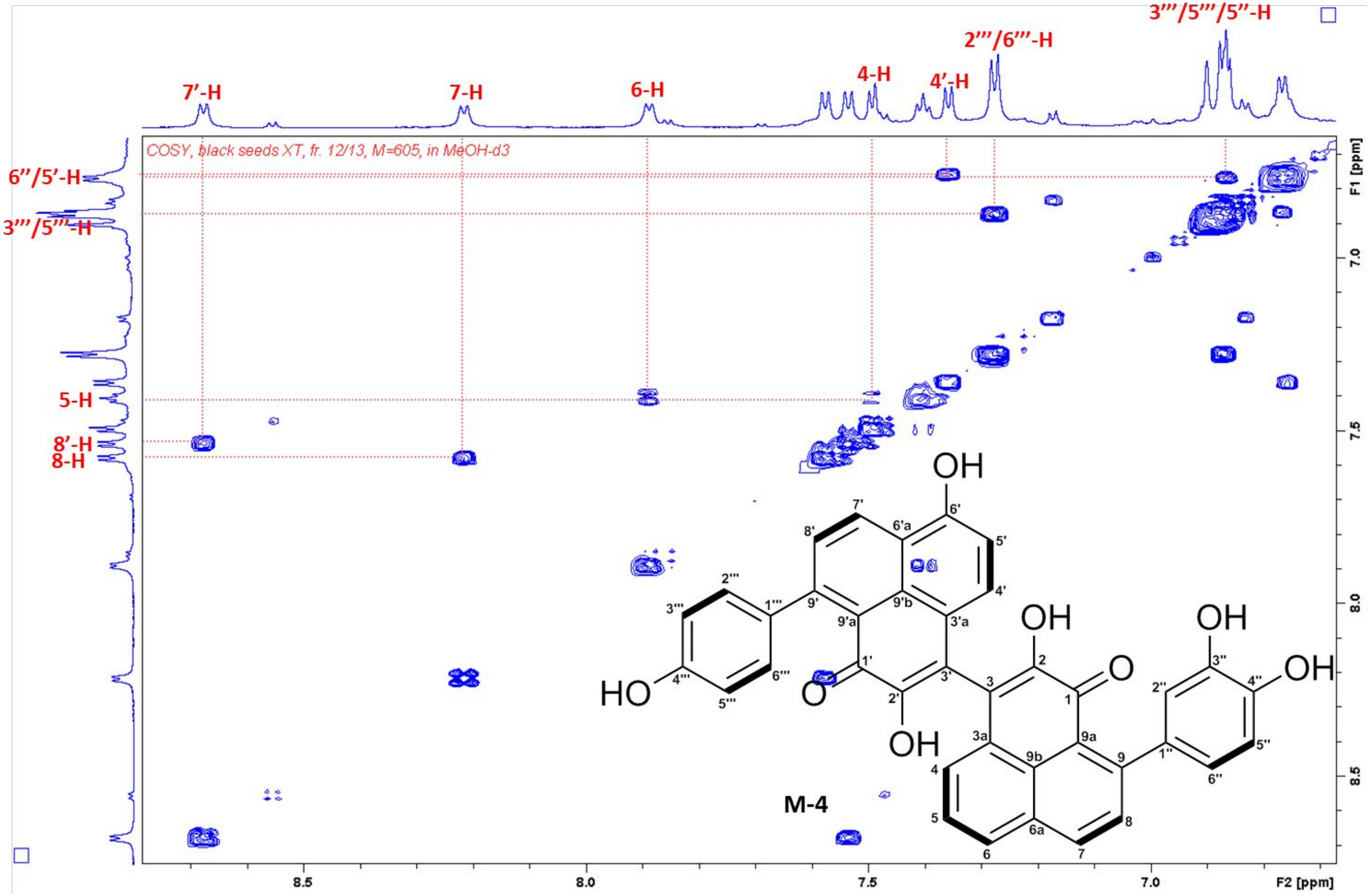

Figure S90. ^{1}H–^{1}H COSY spectrum of compound **M-4** in CD_3OH.

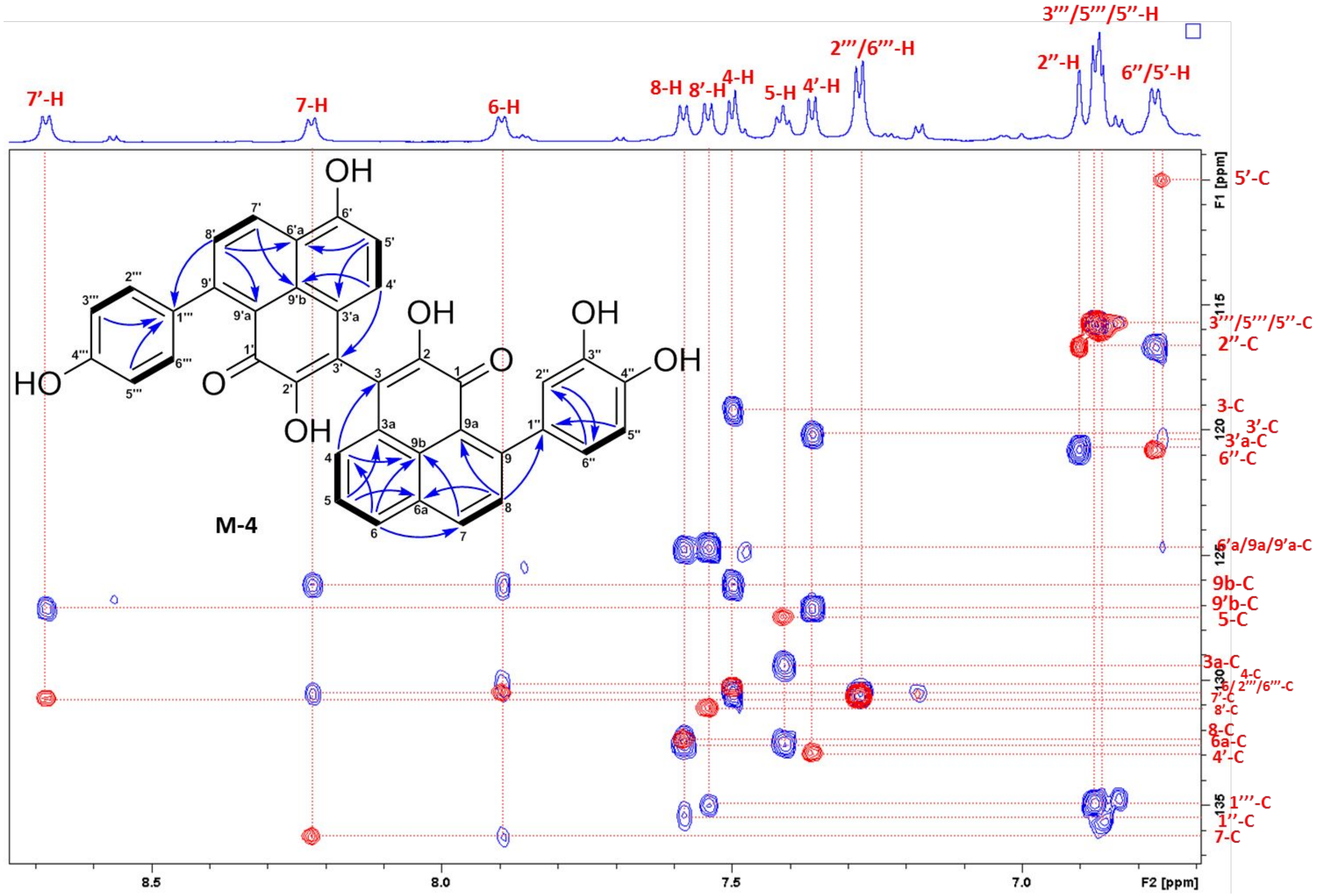

Figure S91. Superimposed HSQC and HMBC spectra of compound **M-4** in CD_3OH (part-1).

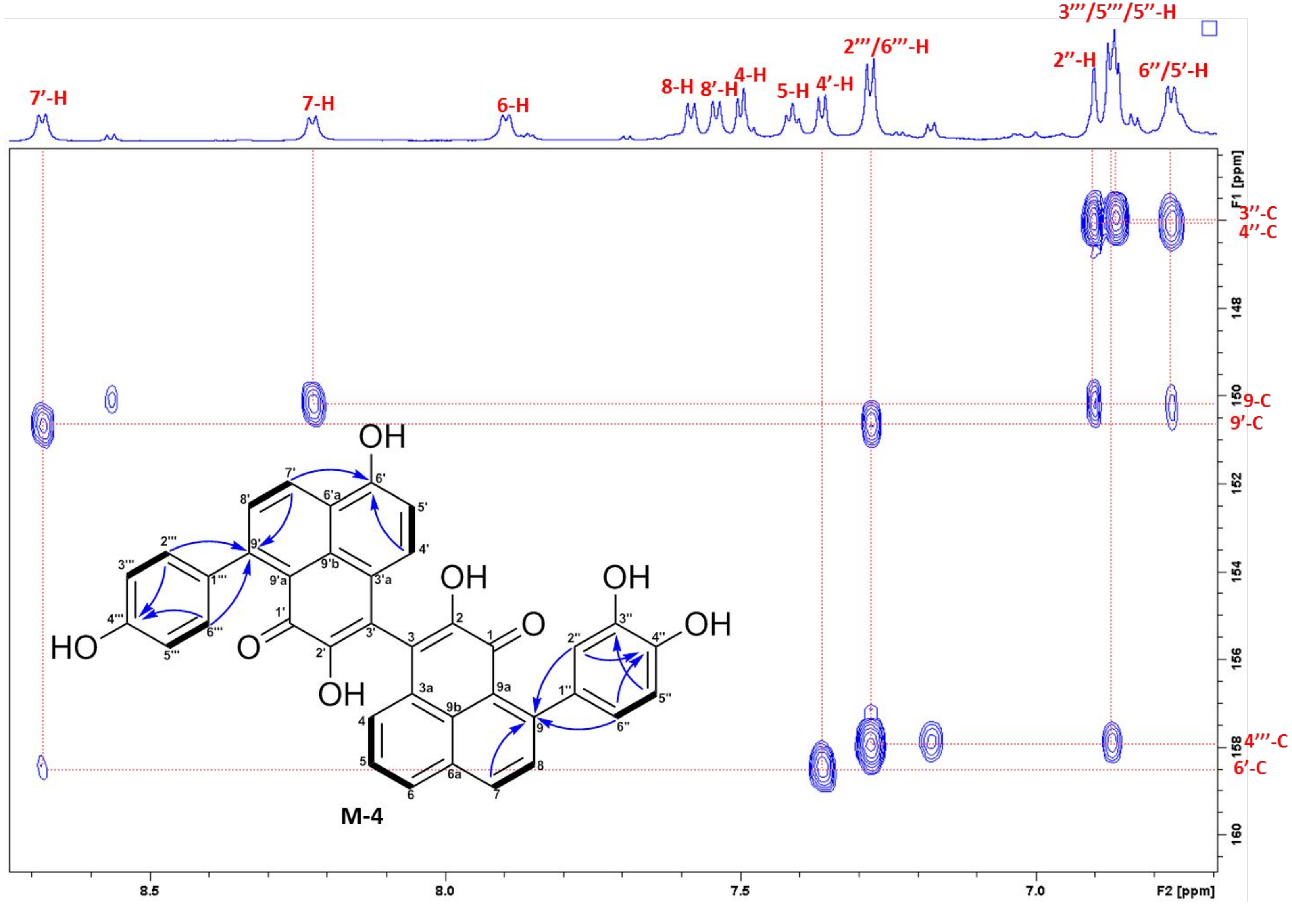

Figure S92. Superimposed HSQC and HMBC spectra of compound **M-4** in CD_3OH (part-2).

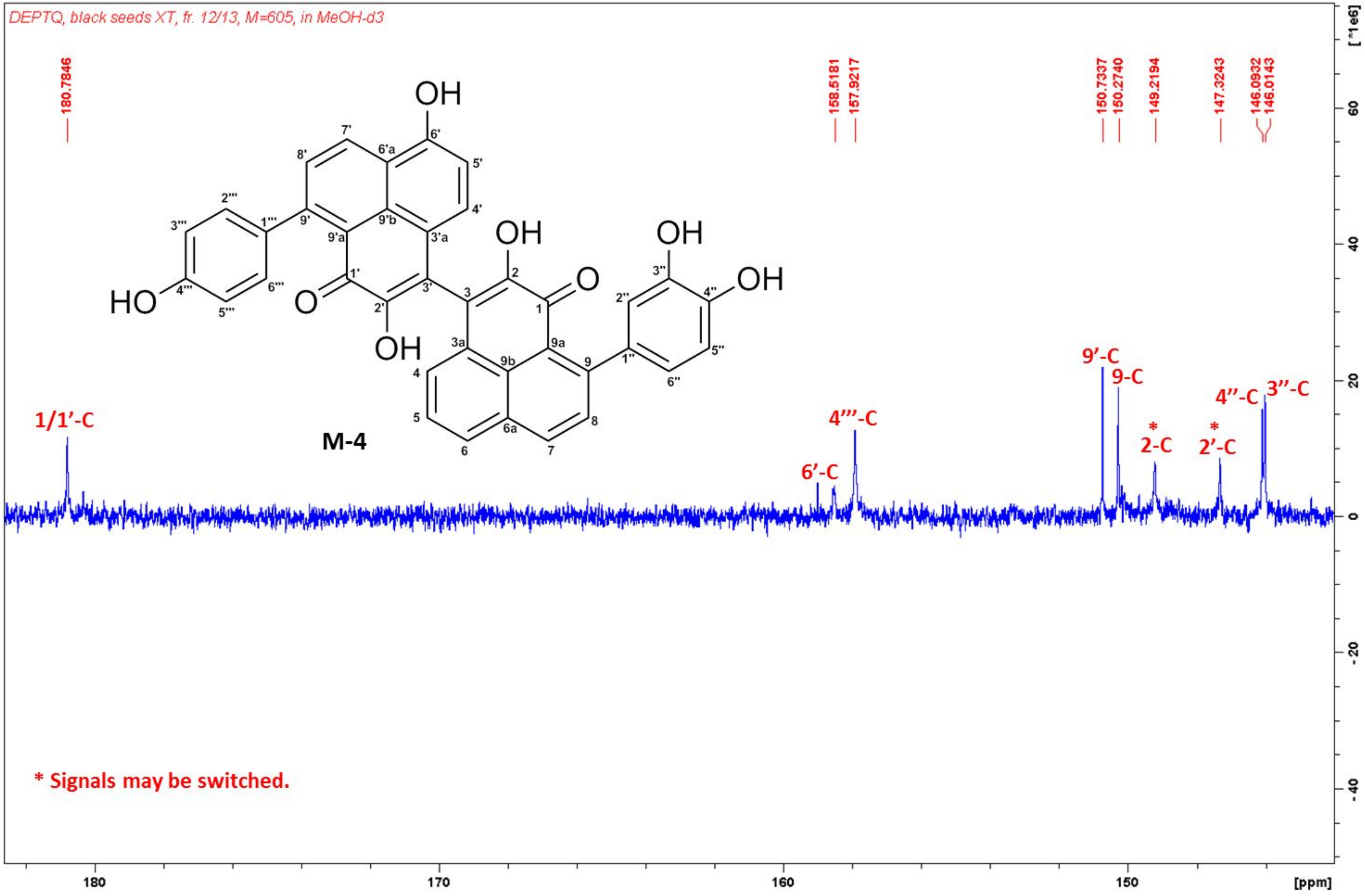

Figure S93. DEPTQ spectrum (175 MHz, CD_3OH) of compound **M-4** (part-1).

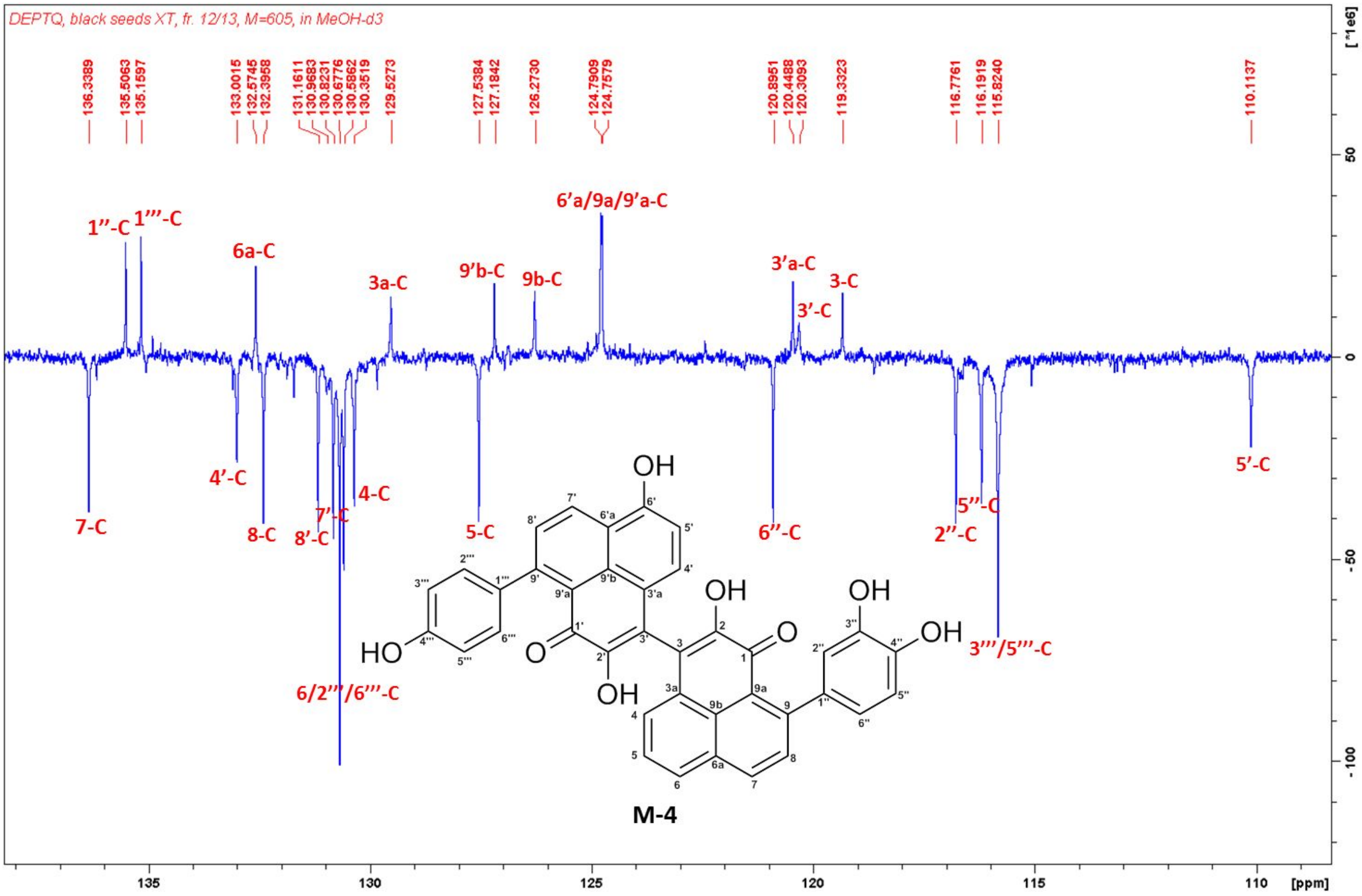

Figure S94. DEPTQ spectrum (175 MHz, CD_3OH) of compound **M-4** (part-2).
